# Supplementary material for: Evaluation of the uptake and delivery of the NHS Health Check programme in England, using primary care data from 9.5 million people: a cross-sectional study
Source: BMJ Open. 2020 Nov 5;10(11):e042963. doi: 10.1136/bmjopen-2020-042963 (PMC7646358; doi:10.1136/bmjopen-2020-042963)
Supplement: Supplementary data [file bmjopen-2020-042963supp001.pdf]

Supplementary Materials

An evaluation of the uptake and delivery of the NHS Health Check Programme in England, using primary care data from 9.5 million people: A cross-sectional study

Contents

Supplementary Methods 2

Supplementary Figures 4

Supplementary Figure 1 - Invitation type for first invitation record by year of invitation among attendees and non-attendees 4

Supplementary Tables 5

Supplementary Table 1: Read codes for NHS Health Check activity codes and prioritisation rules for definition of primary contact with programme 5

Supplementary Table 2: Data extraction rules 6

Supplementary Table 3: Plausible ranges for risk factor measurements 7

Supplementary Table 4: Order of priority for selecting metrics in time window around patient's index date 8

Supplementary Table 5: Derived Ethnic Group Categories 16

Supplementary Table 6: Categories for risk factors - Risk factors by binary cut points 17

Supplementary Table 7: Rules for conflicting risk factors measurements 18

Supplementary Table 8: Intervention risk thresholds for action 18

Supplementary Table 9: Data for attendance by UTLA 19

Supplementary Table 10: Number of invitations recorded for attendees and non-attendees..... 22

Supplementary Table 11: Invitations by financial year..... 23

Supplementary Table 12: Completeness of risk factor measurement 23

Supplementary Table 13: Statin prescription rates 24

Patel R, *et al. BMJ Open* 2020; 10:e042963. doi: 10.1136/bmjopen-2020-042963

Supplementary Methods

Data Management and Cleaning

The data extract was stored within a Structured Query Language (SQL) database and processed using queries within SQL Server Management Studio. Duplicate patient records were removed. Implausible values were re-coded as missing values. Plausible ranges for risk factors, Supplementary Table 3, were defined by DEAC.

Definitions and Study Variables

Individuals were categorised as either NHSHC attendees if they had a Read code for a completed check within the 5-year period, or a non-attendee if they did not. Further details are provided in Supplementary Table 1. Uptake of the programme was defined as the proportion of the total study population who attended.

An index date was generated from the date of an individual's primary NHSHC activity to identify age and the most relevant risk factor measurements for each patient. Risk factor and clinical measurements were selected for analysis if they occurred on the index date, otherwise we took the closest recording within pre-defined time windows set by the DEAC. A full list of variables, Read codes used to define variables, time windows and coding algorithms is available in Supplementary Table 4.

An individual's age in years was estimated based on year of birth and index date and presented in five-year intervals. We derived an ethnic group variable with the aim of generating fewer categories while still representing important ethnic groups for CVD (Supplementary Table 5). We also included Index of Multiple Deprivation (IMD) (2015) national deciles matched at Lower Super Output Area (LSOA) level based on the patient's postcode of residence at the time of data extraction.[1] ONS April 2019 upper tier local authority (UTLA) boundaries were used.[2] Gender was reported as coded in the extract (Male; Female). Learning difficulty, serious mental illness (SMI), blindness, deafness, rheumatoid arthritis and dementia (present/absent) are reported as binary variables.

We present the following risk factors as binary variables, using cut-points defined in consultation with DEAC, Supplementary Table 6; obesity (BMI>30kg/m^2), blood pressure (derived from systolic (>=140mmHg) or diastolic blood pressure (>=90mmHg), cholesterol (total cholesterol >5mmol/L or cholesterol ratio >4), blood glucose (fasting plasma glucose >=7mmol/L or HbA1C>=48mmol/mol), smoking (current), physical activity (general practice physical activity questionnaire = moderately

inactive or inactive), alcohol intake and behaviour (Audit C score >=8), CVD risk score (10 year risk >=10%) and family history of CVD before 60 years. Rules for conflicting measures for the same patient on the same day are available in Supplementary Table 7.

Among attendees, we considered invitations in the 365 days prior to the index date. Time to attendance was derived from the number of days between first recorded invitation and the index date. Invitation type for attendees was grouped into three categories: advanced invitation (invitation recorded prior to date of NHSHC), opportunistic invitation (invitation recorded same date as NHSHC) and missing invitation (invitation not recorded but NHSHC completed). Among non-attendees for whom the primary contact was an invitation, we considered invitations in the 365 days after the index date. The provider delivering the NHSHC (GP staff; third party) was reported as a binary variable.

Among attendees, we present data for delivery of advice, information or referral for diet, alcohol, physical activity, smoking, weight loss and general lifestyle, referrals for diabetes prevention and prescriptions for statins (present/absent) as binary variables. Statin prescribing data was made available by three out of four GP clinical IT system providers, and subsequently a Read code was attached to 60.4% of attendees in the dataset. We present data for any statin prescription on or after the date of NHSHC activity, as individuals with current statin prescriptions would not be eligible for an invitation to the NHSHC. We also present these data among attendees with a risk profile indicating that intervention was appropriate. We defined appropriate thresholds for action of intervention through consultation with the DEAC advisory board. These are available in Supplementary Table 8.

REFERENCES

1. Office for National Statistics. English indices of deprivation 2015 2015 [Available from: https://www.gov.uk/government/statistics/english-indices-of-deprivation-2015.
2. Office for National Statistics. Counties and Unitary Authorities (April 2019) Boundaries EW BFC 2019 [updated November 2019. Available from: https://geoportal.statistics.gov.uk/datasets/counties-and-unitary-authorities-april-2019-boundaries-ew-bfc accessed December 2019.

Supplementary Figures

Supplementary Figure 1 - Invitation type for first invitation record by year of invitation among attendees and non-attendees

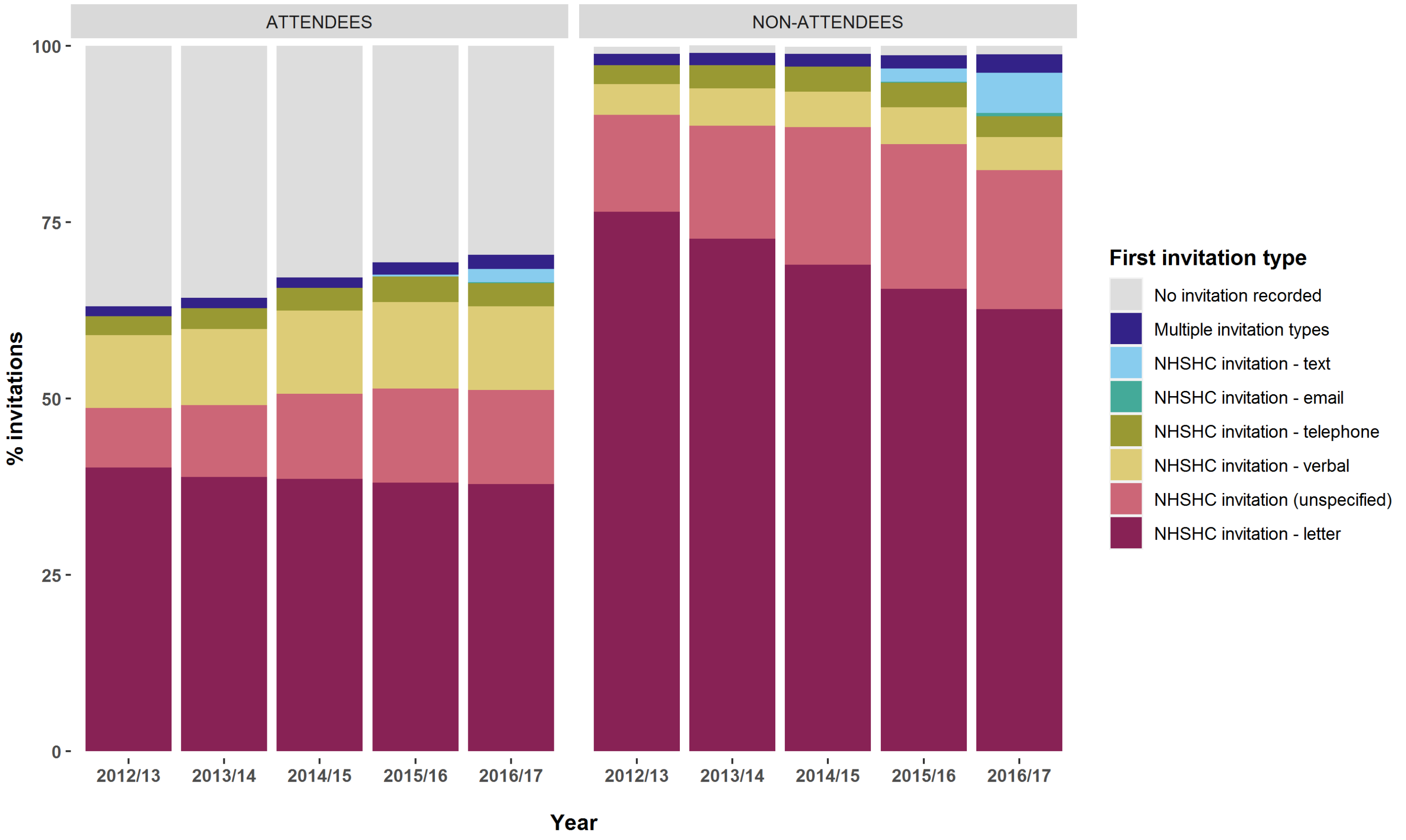

Patel R, *et al. BMJ Open* 2020; 10:e042963. doi: 10.1136/bmjopen-2020-042963

Supplementary Tables

Supplementary Table 1: Read codes for NHS Health Check activity codes and prioritisation rules for definition of primary contact with programme

Order	Clinical NHSHC activity code	Read V2 clinical codes (date introduced)	CTV3 clinical codes (date introduced)	Reported grouping	Criteria
1	Inappropriate	9NSH. (01/10/2013)	Xaaac (01/10/2013)	Excluded from study	Patient has a code recorded as being inappropriate for an NHS Health Check in the data extract
2	Completed	8BAg. (01/04/2010)<br><br>8BAg0 (01/10/2012)	XaRBQ (01/04/2010)<br><br>XaZPq (01/10/2012)	Attendee	Patient has a completed NHS Health Check code recorded in the 5-year period<br><br>**Index date**: date of patient's first completed check code
3	Declined	8IAx. (01/04/2011)	XaX8h (01/04/2011)	Non-attendee	Patient has a declined NHS Health Check code recorded in the 5-year period<br><br>**Index date:** date of patient's first declined code
4	Did not attend	9NiS. (01/04/2010)	XaRAA (01/04/2010)	Non-attendee	Patient has an NHS Health Check not attended code recorded in the 5-year period<br><br>**Index date:** date of patient's first non-attendance code
5	Commenced	8CV9. (01/04/2016)	Xaeab (01/04/2016)	Non-attendee	Patient has a commenced NHS Health Check code recorded in the 5-year period (and no completed/did not attend/declined code recorded in the following 8 weeks)<br><br>**Index date:** date of patient's first commenced code
6	Invitation	9mC.., 9mC0., 9mC1., 9mC2., 9mC3., 9mC4., (01/04/2010)<br><br>9mC5., 9mC6. (01/10/2015)	XaRBR, XaR9z, XaRBS, XaRBT, XaRBU, XaRBV (01/04/2010)<br><br>Xad0C, Xad0D, (01/10/2015)	Non-attendee	Patient has an invitation to attend an NHS Health Check code recorded in the 5-year period (and no follow up (non-invitation) code recorded within the following 6 months)<br><br>**Index date:** date of patient's first invitation code

Supplementary Table 2: Data extraction rules

Event of interest (NHS Health check offer, completed, DNA, declined …) — **GPES EXTRACT**

Category data item	Details data item	before event	1 month	after event
Patients demographics & equity measures	Carer status	latest code before event	1 month	
	Learning disabilities, serious mental illness, blindness, deafness, dementia	include only first recorded code (if not 'resolved')		
	Deprivation (calculated), age (calculated), gender, ehtnicity, first language	latest available code		
CVD risk factor	Smoking status, height, weight, BMI, waist circum., alcohol use, physical activity, CVD risk score	all codes 2 years before event		all codes after event
Tests results	Blood pressure, pulse, cholesterol & HDL, glucose/ HbA1c, eGFR, ACR	all codes 2 years before event		all codes after event
CVD diagnosis (**)	Family history CVD, CHD, CKD, diabetes type 2, TIA, stroke, AF, Heart failure, hypertension, familial hypercholesterolemia, hypercholesterolemia, hyperlipidaemia, PAD. (+ non-diabetic hyperglycaemia, impaired glucose regulation, impaired fasting glycaemia, impaired glucose tolerance) (+ rheumatoid arthritis)	all codes (if not resolved)		
Referrals	Referral to NHS DPP (start 2016)	all codes		
	Advice, signposting, referral to lifestyle services, referral for other CVD assessment (diabetes, hypertension, …)	latest code before event		all codes within 1 year of event
Prescribed CVD medication	Lipid regulation, anti-hypertensive, anti-coagulant, diabetes treatment, AF treatment	all codes 2 years before event		all codes within 1 year of event

Supplementary Table 3: Plausible ranges for risk factor measurements

**Risk factor**	**Plausible measurement range (inclusive unless stated)**
Alcohol risk score<br>(AUDIT; AUDITC; FAST)	0 – 40
Blood pressure - systolic	70 – 300 mmHg
Blood pressure - diastolic	20 – 150 mmHg
BMI	12 – 90 kg/m^2
Cholesterol – total	1 – 40 (exclusive)
Cholesterol – HDL	0.5 – 5
Cholesterol – ratio	0.2 – 80
Fasting Plasma Glucose (FPG)	0 (exclusive) – 100
HbA1c	20 – 195 mmol/mol
Height	100 – 230 cm
CVD risk score	0 – 100
Weight	20 – 250 kg

Patel R, *et al. BMJ Open* 2020; 10:e042963. doi: 10.1136/bmjopen-2020-042963

Supplementary Table 4: Order of priority for selecting metrics in time window around patient's index date

Metric	First priority	Second priority	Third priority	Derivation / other prioritisation rules	Clinical codes (Read V2)	Clinical codes (CTV3)
**Patient characteristics**						
Ethnic group	Ethnic group recorded in patient's GPES profile at time of data extraction (31/3/2018)	Most recent ethnic group recorded via a clinical code (looking over whole data extract)	n/a	n/a	9S...% , 9T...% , 9t...% , 9i...%	XaBEN%
Blindness	On index date	Anytime before index date (most proximal to index date used)	n/a	n/a	6689. , 6688. , 668D. , 668C.	6689.% , XaW0l , XaCGX% , XaLMz
Deafness	On index date	Anytime before index date (most proximal to index date used)	n/a	n/a	F599. , F591B , F591E , F59A. , F5919	XaRE4 , XaZuB , XaZuE , XaaLf , XaRE5 , Xa0PN
Dementia	On index date	Anytime before index date (most proximal to index date used)	n/a	n/a	Eu02.% , E00..% , Eu01.% , E02y1 , E012.% , Eu00.% , E041. , Eu041 , F110.- F112. , F116. , F118. , F21y2 , A410. , A411.%	X002w% (excluding X003E , X003F , X001T) , Eu02.% , XE1Xt , E00z. , E02y1
Learning Disability	On index date	Anytime before index date (most proximal to index date used)	n/a	n/a	E3...% , Eu7..% , Eu814 , Eu815 , Eu816 , Eu817 , Eu81z , 918e. , Eu818	E3...% , XaQZ4 , XaQZ3 , XaKYb , XaREt , XaREu , Eu81z , XaaiS , Xabk1
Severe Mental Illness	On index date	Anytime before index date (most proximal to index date used)	n/a	n/a	E10..% , E110.% , E111.% , E1124 , E1134 , E114.- E117z , E11y.% (excluding E11y2) , E11z. , E11z0 , E11zz , E12..% , E13..% (excluding E135.) , E2122 , Eu2..% , Eu30.%	X00S6% (excluding Xa9B0% , E14..%) , X00SL , X00SM% , X00SJ% , XSGon , E11z. , E11z0 , E11zz , XE1ZZ , XE1Ze , XaX54 , XaX53 , E130. , E1124 , E1134

					, Eu31.% , Eu323 , Eu328 , Eu333 , Eu32A , Eu329	
**CVD risk factors**						
Family history of CVD	On index date	Anytime before index date (most proximal to index date used)	Anytime after index date (most proximal to index date used)	n/a	12C.. , 12C2. , 12C3. , 12C4. , 12C5. , 12CA. , 12CB. , 12CC. , 12CD. , 12CE. , 12CF. , 12CG. , 12CH. , 12CI. , 12CL. , 12CM. , 12CN. , 12CP. , 12CV. , 12CW. , 12CZ.	XaP9K , XaP9M , ZV174 , XE24Z , XaLQq , Xa6aj% , XM1Jg , XM1Jw% , XaP9K , XaP9M
Rheumatoid arthritis	On index date	Anytime before index date (most proximal to index date used)	**Attendees:** n/a<br><br>**Non-attendees:** Anytime after index date (most proximal to index date used)	n/a	N040.% , N041. , N042.% (excluding N0420) , N047. , N04X. , N04y0 , N04y2 , Nyu11 , Nyu12 , Nyu1G , Nyu10 , G5yA. , G5y8.	N040.% , XE1DU , X705I , G5y8.
Alcohol AUDIT/AUDIT-C/FAST	On index date	Most proximal score to index date for each of AUDIT, AUDIT-C and FAST used.<br><br>**Attendees:** Up to 365 days before index date<br><br>**Non-attendees:** Anytime before index date	Most proximal score to index date for each of AUDIT, AUDIT-C and FAST used.<br><br>**Attendees:** Up to 90 days after index date<br><br>**Non-attendees:** Anytime after index date	No AUDIT-C/FAST/AUDIT score available: risk factor is **missing**<br><br>AUDIT-C or FAST assessment is positive, but no AUDIT score available: risk factor is **missing**<br><br>AUDIT-C (and/or) FAST assessment is negative: risk factor is **low risk**<br><br>AUDIT score available and greater than or equal to 8: risk factor is **high risk**	38D4. (AUDIT-C),<br>388u. (FAST),<br>38D3. (AUDIT)	XaORP (AUDIT-C),<br>XaNO9 (FAST),<br>XM0aD (AUDIT)

Blood pressure	On index date	Systolic and diastolic BP recordings recorded most proximal to index date used.<br><br>**Attendees:** Up to 365 days before index date<br><br>**Non-attendees:** Anytime before index date	Systolic and diastolic BP recordings recorded most proximal to index date used.<br><br>**Attendees:** Up to 90 days after index date<br><br>**Non-attendees:** Anytime after index date	On examination (O/E) readings considered only.<br><br>Systolic BP or Diastolic BP is unavailable: risk factor is **missing**	246..% (excluding 2460. , 2468. , 246H. , 246I. , 246K. , 246L. , 246M. , 246h. , 246i. , 246j. , 246k. , 246n.% , 246o.%)	X773t% (excluding XaI9f , XaI9g , XaZvo , XaZxj , X779b , X779R , X779T , X779W , XaYai , XaYg8 , XaYg9 , Xabhx , Xac5K , Xac5L , Xaedn%) , 246..% (excluding 2460. , 2468. , XaCFN , XaCFO)
Blood glucose	On index date	HbA1c and Fasting Plasma Glucose recorded most proximal to index date considered.<br><br>**Attendees:** Up to 365 days before index date<br><br>**Non-attendees:** Anytime before index date	HbA1c and Fasting Plasma Glucose recorded most proximal to index date considered.<br><br>**Attendees:** Up to 90 days after index date<br><br>**Non-attendees:** Anytime after index date		HbA1c:<br>42W5. , 42W50 , 42W51<br><br>Fasting Plasma Glucose:<br>44g1.	HbA1c:<br>XaPbt , Xaezd , Xaeze<br><br>Fasting Plasma Glucose:<br>44g1.
Body mass index	On index date	Most proximal to index date used.<br><br>**Attendees:** Up to 365 days before index date<br><br>**Non-attendees:** Anytime before index date	Most proximal to index date used.<br><br>**Attendees:** Up to 90 days after index date<br><br>**Non-attendees:** Anytime after index date	If BMI is unavailable but height and weight are, BMI is calculated (BMI = kg/m^2)<br><br>Height and weight are not used if BMI is available	BMI:<br>22K..% (excluding 22K9.% , 22KA.)<br><br>Weight:<br>22A..% (excluding 22A7.-22A9.) , 9NSa. , 8IAH.<br><br>Height:<br>229..% (excluding 2296.) , 9NSZ. , 8IHM.	BMI:<br>22K..% (excluding XaVwA% , X76CN , XaZMj) , Xa7wG%<br><br>Weight:<br>22A..% , 22AA. , X76C3 , XaesG , XaQ7T<br><br>Height:

						229..% (excluding 2296.) , XaesF , Xaef4
Cholesterol (ratio)	On index date	Most proximal to index date used.<br><br>**Attendees:** Up to 365 days before index date<br><br>**Non-attendees:** Anytime before index date	Most proximal to index date used.<br><br>**Attendees:** Up to 90 days after index date<br><br>**Non-attendees:** Anytime after index date	If cholesterol ratio is unavailable but total and HDL cholesterol are, the cholesterol ratio is calculated (ratio = total/HDL)<br><br>Total and HDL cholesterol are not used if cholesterol ratio is available	Cholesterol:<br>44O5. , 44PH. , 44P5. , 44PF. , 44PJ. , 44P.. , 44OE. , 44P1. , 44P2. , 44P3. , 44P4. , 44PK. , 44PZ. , 44l2. , 44lF. , 44lG. , 662a.<br><br>HDL cholesterol:<br>44P5. , 44PB. , 44PC. , 44d3. , 44d2.	Cholesterol:<br>XaFs9 , XSK14 , 44P5. , 44PF , 44PJ. , XalRd , XE2eD% , 44P1. , 44P2. , 44P3. , 44P4. , 44PH. , XaERR , XaEUq , XaEUr , X772L<br><br>HDL cholesterol:<br>X772M , 44P5. , 44PB. , 44PC. , XaEVr , 44d3. , 44d2.
Physical activity (GPPAQ)	On index date	Most proximal to index date used.<br><br>**Attendees:** Up to 365 days before index date<br><br>**Non-attendees:** Anytime before index date	Most proximal to index date used.<br><br>**Attendees:** Up to 90 days after index date<br><br>**Non-attendees:** Anytime after index date	n/a	138b. , 138a. , 138Y. , 138X. , 38Dh.	XaPPE , XaPPD , XaPPB , XaPP8 , XaXX5
CVD risk score	On index date	QRISK/QRISK2 and Framingham risk score recorded most proximal to index date used.<br><br>**Attendees:** Up to 365 days before index date	QRISK/QRISK2 and Framingham risk score recorded most proximal to index date used.<br><br>**Attendees:** Up to 90 days after index date	QRISK or QRISK2 score recorded most proximal to index date is used if available.<br><br>If QRISK and QRISK2 unavailable, Framingham score is used.	QRISK/QRISK2:<br>8IEL., 8IEV., 38DF., 38DP.<br><br>Framingham:<br>38DR.	QRISK/QRISK2:<br>XaYzy, XaZdA, XaPBq, XaQVY<br><br>Framingham:<br>XaQaG

		**Non-attendees:** Anytime before index date	**Non-attendees:** Anytime after index date			
Smoking status	On index date	Most proximal to index date used.<br><br>**Attendees:** Up to 365 days before index date<br><br>**Non-attendees:** Anytime before index date	Most proximal to index date used.<br><br>**Attendees:** Up to 90 days after index date<br><br>**Non-attendees:** Anytime after index date	Lookup used to map smoking status to binary categories: Non-smoker; Current smoker	Non-smoker:<br>1371, 137A., 137l., 137N., 137O., 137S.,<br><br>Current smoker:<br>137.., 137C., 137e., 137h., 137m., 137P., 137Q., 137R., 137V., 137X., 137Y.,	Non-smoker:<br>1371, 1377, 1378, 1379, 137B., 137F., 137K., 137T., Ub0p1, Ub1na, Xa1bv, XaQ8V, XE0oj, XE0ok, XE0ol, XE0om, XE0on, XE0op, XE0oh<br><br>Current smoker:<br>1372, 1373, 1374, 1375, 1376, 137D., 137G., 137J., 137Z., Ub1tI, Ub1tJ, Ub1tK, Ub1tR, Ub1tS, Ub1tU, Ub1tW, XaIIu, XaIkW, XaIkX, XaIkY, XaItg, XaJX2, XaLQh, XaWNE, XaZIE, XE0oq, XE0or
**Interventions – attendees only**						
Advice, informatio n, referral – ALCOHOL	On index date	Up to 365 days after index date	n/a	n/a	Advice, information and any brief intervention given on alcohol usage:<br>67H0. , 67A5. , 8CAM. , 8CAM0 , 8CAv. , 8CE1. , 9k1A. , 8IAF. , 8IAt. , 9k11. , 9k14. , ZV6D6 , 6792. , 8CdK.<br><br>Referral regarding alcohol usage:	Advice, information and any brief intervention given on alcohol usage:<br>XaJIr , Xa1dA , 67A5. , XaFvp , XaXan , XaPmB , 8CE1. , XaPPv , XaPty , XaX4S , XaKAC , XaKAo , ZV6D6 , 6792. , Xac6H<br><br>Referral regarding alcohol usage:

					8HkG. , 8H7p. , 8HHe.	XaYWV , XaIPn , XaKUg , XaPna , XaORR
Advice, informatio n, referral – DIET	On index date	Up to 365 days after index date	n/a	n/a	Advice, signposting or information on diet: 67H7. , 8CA4. , 8CA40 , 6799.<br><br>Referral regarding diet: 8H76. , 8H760 , 8HHE.	Advice, signposting or information on diet: XaQaU , 8CA4. , XaXTD , Xa2jQ , XE0i1 , Xa2hD , 6799.<br><br>Referral regarding diet: XaBSz , XaAhZ , XaAha , XaJSp , XaAdX , XaAdY , XaAdZ
Advice, informatio n, referral – LIFESTYLE	On index date	Up to 365 days after index date	n/a	n/a	67H..% , 8Hlu.	XaEFY% , Xaam2
Advice, informatio n, referral – PHYSICAL ACTIVITY	On index date	Up to 365 days after index date	n/a	n/a	Advice, signposting or information on physical activity: 67H2. , 8CA5. , 9Oq3. , 6798. , 8CA52 , 8Cd4. , 8IAv. , 8HBN.<br><br>Referral regarding physical activity: 8H7q. , 8H7q0 , 8HHc. , 8HkX. , 8BAH.	Advice, signposting or information on physical activity: XaJIt , Xa1dN , 8CA5. , XM18T , XaPjx , 6798. , XabFV , XaREx , XaX5H , XaREy<br><br>Referral regarding physical activity: XaIPu , XaR5C , XaKRq , XaREh , XaCmH
Advice, informatio n, referral	On index date	Up to 365 days after index date	n/a	n/a	Support and refer Stop Smoking Service/Advisor:	Support and refer Stop Smoking Service/Advisor:

– SMOKING					8CAL. , 8HTK. , 8HkQ. , 8H7i. , 8IAj. , 8IEK. , 9N2k. , 13p50 , 9Ndf. , 9Ndg. , 8T08. , 8IEo.<br><br>Advice, signposting or information on smoking: 67H1. , 8CAL. , 67A3. , 8CAg. , 6791. , 8IAj. , 8CdB.	Ua1Nz , XaFw9 , XaQT5 , XaItC , XaIye , XaW0h , XaX5W , XaX5X , XaRFh , XaREz , XaaDy , XaaDx<br><br>Advice, signposting or information on smoking: XaJIs , Ua1Nz , 67A3. , Ua1O0 , XaLD4 , 6791. , XaRFh , XaXnG
Advice, information, referral – WEIGHT	On index date	Up to 365 days after index date	n/a	n/a	Advice, signposting or information on weight management: 67I9. , 8CA40 , 8Cd7. , 66CQ. , 679P. , 8CdC. , 8IAu.<br><br>Referral regarding weight management: 8HHH. , 8HHH1 , 8HHH0 , 8H4n.	Advice, signposting or information on weight management: XaADJ , Xa1dF , XaX5F , XaX5k , XaKHd , XaXnI , XaX5G<br><br>Referral regarding weight management: XaJSu , XaZKe , XaXZ9 , XaZKi
Diabetes Prevention Programme referral	On index date	Up to 365 days after index date	n/a	n/a	679m4, 679m0, 679m1, 679m2	XaeDH, XaeCw, XaeCz, XaeD0
Statin prescriptions	On index date	Up to 365 days after index date	n/a	n/a	bxi..% , bxg..% , bxe..% , bxk..% , bxd..%<br><br><u>DM+D codes</u> (EMIS): 134489001, 319996000, 319997009, 320000009,	bxi..% , x01R2% , x01R3% , bxk..% , bxd..%

					320006003,<br>320012008,<br>320013003,<br>320014009,<br>320029006,<br>320030001,<br>320031002,<br>408036003,<br>408037007,<br>409108001,<br>4896711000001108	

Patel R, *et al. BMJ Open* 2020; 10:e042963. doi: 10.1136/bmjopen-2020-042963

Supplementary Table 5: Derived Ethnic Group Categories

**Ethnic group**	**Subgroups (with ONS codes)**
White	A = White British
	B = Irish
	C = Any other White background
	T = White: Gypsy or Irish Traveller
Indian	H = Indian
Pakistani	J = Pakistani
Bangladeshi	K = Bangladeshi
Black African	N = African
Black Caribbean	M = Caribbean
Chinese	R = Chinese
Other Asian	L = Any other Asian background
Other Ethnic Group	D = White and Black Caribbean
	E = White and Black African
	F = White and Asian
	G = Any other mixed background
	P = Any other Black background
	S = Any other ethnic group
	W = Other ethnic group: Arab
Unknown	X = Unknown/No information
	Z = Not stated

Supplementary Table 6: Categories for risk factors - Risk factors by binary cut points

Risk factors by binary risk cut-offs

Risk factor	High risk threshold/ cutpoint	Risk category	Attendees n (%)	Non-attendees n(%)	Total
Alcohol > Low Risk	Full AUDIT score 8 or more	Missing	3,150,667 (61.7)	3,823,634 (83.3)	6,974,301
		Low risk	1,830,799 (35.9)	714,947 (15.6)	2,545,746
		High risk	121,292 (2.4)	53,640 (1.2)	174,932
Possible Diabetes	HbA1C ≥ 48 or FPG ≥ 7	Missing	2,558,719 (50.1)	2,590,405 (56.4)	5,149,124
		Low risk	2,460,489 (48.2)	1,885,332 (41.1)	4,345,821
		High risk	83,550 (1.6)	116,484 (2.5)	200,034
High Blood Pressure	Systolic BP ≥ 140 or Diastolic BP ≥ 90	Missing	217,714 (4.3)	1,086,797 (23.7)	1,304,511
		Low risk	3,636,511 (71.3)	2,404,097 (52.4)	6,040,608
		High risk	1,248,533 (24.5)	1,101,327 (24)	2,349,860
Obesity	BMI ≥ 30	Missing	187,402 (3.7)	2,064,936 (45)	2,252,338
		Low risk	3,700,522 (72.5)	1,755,019 (38.2)	5,455,541
		High risk	1,214,834 (23.8)	772,266 (16.8)	1,987,100
High Cholesterol	Total cholesterol >5mmol/L or Ratio > 4	Missing	282,100 (5.5)	2,286,595 (49.8)	2,568,695
		Low risk	1,519,485 (29.8)	696,458 (15.2)	2,215,943
		High risk	3,301,173 (64.7)	1,609,168 (35.0)	4,910,341
CVD risk score	10 or more	Missing	1,036,820 (20.3)	3,197,683 (69.6)	4,234,503
		Low risk	3,014,556 (59.1)	979,685 (21.3)	3,994,241
		High risk	1,051,382 (20.6)	414,853 (9)	1,466,235
Family history of CVD	Clinical code present for a CVD event before 60 years old in a first degree relative	No	4,910,543 (96.2)	4,561,766 (99.3)	9,472,309
		Yes	192,215 (3.8)	30,455 (0.7)	222,670
Physical Activity	GPPAQ “moderately inactive” or “inactive”	Missing	1,812,161 (35.5)	3,952,015 (86.1)	5,764,176
		Low risk	2,184,515 (42.8)	392,263 (8.5)	2,576,778
		High risk	1,106,082 (21.7)	247,943 (5.4)	1,354,025
Smoking	Current smoker	Missing	221,351 (4.3)	1,296,474 (28.2)	1,517,825
		Low risk	4,066,412 (79.7)	2,325,196 (50.6)	6,391,608
		High risk	814,995 (16)	970,551 (21.1)	1,785,546

Patel R, *et al. BMJ Open* 2020; 10:e042963. doi: 10.1136/bmjopen-2020-042963

Supplementary Table 7: Rules for conflicting risk factors measurements

Rules for processing conflicting risk factor measurements for the same patient on the same day

Risk factor	Rule applied
Smoking status; Physical activity status (from GPPAQ)	Records deleted if descriptive statuses are conflicting (e.g. “smoker” and “non-smoker” recorded on the same day)
Blood pressure	Record with lowest systolic measurement taken
BMI; height; weight; QRISK/QRISK2 score; Framingham score; total cholesterol; HDL cholesterol; Cholesterol ratio; HbA1c; FPG	Measurements recoded as missing (unclear which is correct)

Supplementary Table 8: Intervention risk thresholds for action

Intervention type	Advice or Information given	High risk threshold for action
**Advice, information or referral**	Alcohol usage	Alcohol: FULL AUDIT 8 or more
	Diet	Overweight (BMI ≥ 25)
	Physical activity	GPPAQ “moderately inactive” or “inactive”
	Lifestyle/Counselling	CVD risk score 10 or more
	Smoking cessation	Current smoker
	Weight management	Overweight (BMI ≥ 25)
**Diabetes referral**	Diabetes Prevention Programme (DPP) referral	Blood glucose: RAISED risk HbA1C ≥ 42 and < 48 or FPG ≥ 5.5 and < 7
**Statin prescription**	Statins prescribed	CVD risk score 10 or more

Patel R, *et al. BMJ Open* 2020; 10:e042963. doi: 10.1136/bmjopen-2020-042963

Supplementary Table 9: Data for attendance by UTLA

Number of NHS Health Check invitees and attendees with attendance rate by Upper Tier Local Authority of patient's residence

UTLA Code	UTLA	Invitees	Attendees	Attendance rate	Lower 95% CI	Upper 95% CI
E10000014	Hampshire	179,937	152,318	84.7	84.5	84.8
E09000030	Tower Hamlets	42,098	34,660	82.3	82.0	82.7
E09000028	Southwark	41,938	33,536	80.0	79.6	80.3
E09000025	Newham	51,556	40,706	79.0	78.6	79.3
E09000012	Hackney	37,636	29,713	78.9	78.5	79.4
E08000001	Bolton	64,013	49,792	77.8	77.5	78.1
E09000001	City of London	1,176	910	77.4	74.9	79.7
E08000017	Doncaster	19,869	14,736	74.2	73.6	74.8
E06000053	Isles of Scilly	482	353	73.2	69.1	77.0
E09000022	Lambeth	35,757	26,172	73.2	72.7	73.7
E09000010	Enfield	38,337	27,370	71.4	70.9	71.8
E09000005	Brent	68,977	48,573	70.4	70.1	70.8
E08000002	Bury	31,309	21,979	70.2	69.7	70.7
E09000002	Barking and Dagenham	36,578	25,402	69.4	69.0	69.9
E09000026	Redbridge	51,865	35,942	69.3	68.9	69.7
E06000021	Stoke-on-Trent	55,178	37,866	68.6	68.2	69.0
E06000008	Blackburn with Darwen	17,852	12,192	68.3	67.6	69.0
E08000030	Walsall	49,943	33,947	68.0	67.6	68.4
E09000023	Lewisham	26,396	17,838	67.6	67.0	68.1
E08000016	Barnsley	51,420	34,550	67.2	66.8	67.6
E09000009	Ealing	61,109	40,012	65.5	65.1	65.9
E06000039	Slough	16,191	10,600	65.5	64.7	66.2
E09000017	Hillingdon	45,539	29,447	64.7	64.2	65.1
E08000007	Stockport	44,540	28,763	64.6	64.1	65.0
E08000005	Rochdale	36,853	22,967	62.3	61.8	62.8
E09000015	Harrow	29,691	18,476	62.2	61.7	62.8
E06000047	County Durham	120,544	73,877	61.3	61.0	61.6
E09000019	Islington	38,209	23,415	61.3	60.8	61.8
E08000033	Calderdale	41,631	25,247	60.6	60.2	61.1
E09000031	Waltham Forest	50,680	30,720	60.6	60.2	61.0
E08000034	Kirklees	97,779	59,189	60.5	60.2	60.8
E10000029	Suffolk	147,142	89,051	60.5	60.3	60.8
E09000032	Wandsworth	57,469	34,442	59.9	59.5	60.3
E08000025	Birmingham	178,771	106,909	59.8	59.6	60.0
E06000036	Bracknell Forest	19,697	11,778	59.8	59.1	60.5
E10000019	Lincolnshire	200,192	119,037	59.5	59.2	59.7
E06000046	Isle of Wight	24,068	14,251	59.2	58.6	59.8
E08000004	Oldham	34,227	20,184	59.0	58.4	59.5
E06000031	Peterborough	44,281	26,027	58.8	58.3	59.2
E06000025	South Gloucestershire	59,350	34,683	58.4	58.0	58.8

E09000014	Haringey	29,867	17,448	58.4	57.9	59.0
E08000022	North Tyneside	40,154	23,434	58.4	57.9	58.8
E06000013	North Lincolnshire	24,121	13,870	57.5	56.9	58.1
E10000017	Lancashire	218,451	125,262	57.3	57.1	57.5
E06000005	Darlington	27,163	15,546	57.2	56.6	57.8
E06000011	East Riding of Yorkshire	12,161	6,894	56.7	55.8	57.6
E10000003	Cambridgeshire	116,035	65,679	56.6	56.3	56.9
E08000018	Rotherham	7,953	4,476	56.3	55.2	57.4
E06000016	Leicester	40,169	22,547	56.1	55.6	56.6
E06000034	Thurrock	32,083	17,982	56.0	55.5	56.6
E09000018	Hounslow	44,165	24,579	55.7	55.2	56.1
E10000006	Cumbria	120,237	65,183	54.2	53.9	54.5
E06000040	Windsor and Maidenhead	21,114	11,418	54.1	53.4	54.7
E06000057	Northumberland	75,940	40,859	53.8	53.4	54.2
E10000034	Worcestershire	141,667	76,000	53.6	53.4	53.9
E10000012	Essex	331,942	178,015	53.6	53.5	53.8
E10000024	Nottinghamshire	198,187	106,221	53.6	53.4	53.8
E09000024	Merton	43,144	23,114	53.6	53.1	54.0
E06000022	Bath and North East Somerset	44,466	23,810	53.5	53.1	54.0
E06000004	Stockton-on-Tees	35,341	18,857	53.4	52.8	53.9
E08000014	Sefton	48,044	25,630	53.3	52.9	53.8
E08000026	Coventry	64,356	34,306	53.3	52.9	53.7
E06000002	Middlesbrough	23,037	12,243	53.1	52.5	53.8
E08000019	Sheffield	80,302	42,628	53.1	52.7	53.4
E10000007	Derbyshire	197,165	104,520	53.0	52.8	53.2
E08000035	Leeds	174,645	92,288	52.8	52.6	53.1
E06000003	Redcar and Cleveland	25,185	13,304	52.8	52.2	53.4
E08000015	Wirral	80,558	42,456	52.7	52.4	53.0
E10000027	Somerset	75,851	39,814	52.5	52.1	52.8
E10000015	Hertfordshire	200,153	104,948	52.4	52.2	52.7
E09000016	Havering	42,627	22,305	52.3	51.9	52.8
E06000012	North East Lincolnshire	38,004	19,816	52.1	51.6	52.6
E08000029	Solihull	32,476	16,930	52.1	51.6	52.7
E10000013	Gloucestershire	137,245	71,077	51.8	51.5	52.1
E06000045	Southampton	33,058	17,102	51.7	51.2	52.3
E06000038	Reading	8,400	4,338	51.6	50.6	52.7
E06000027	Torbay	31,524	16,268	51.6	51.1	52.2
E06000024	North Somerset	40,162	20,498	51.0	50.5	51.5
E06000001	Hartlepool	12,989	6,616	50.9	50.1	51.8
E09000027	Richmond upon Thames	33,597	17,021	50.7	50.1	51.2
E06000033	Southend-on-Sea	48,006	24,182	50.4	49.9	50.8
E06000054	Wiltshire	114,656	57,526	50.2	49.9	50.5
E10000031	Warwickshire	102,623	51,428	50.1	49.8	50.4
E09000029	Sutton	24,049	11,959	49.7	49.1	50.4

Patel R, *et al. BMJ Open* 2020; 10:e042963. doi: 10.1136/bmjopen-2020-042963

E10000025	Oxfordshire	175,246	87,139	49.7	49.5	50.0
E06000056	Central Bedfordshire	73,732	36,607	49.6	49.3	50.0
E08000021	Newcastle upon Tyne	32,888	16,287	49.5	49.0	50.1
E10000021	Northamptonshire	155,686	76,979	49.4	49.2	49.7
E09000003	Barnet	52,312	25,849	49.4	49.0	49.8
E08000006	Salford	34,274	16,934	49.4	48.9	49.9
E06000019	Herefordshire, County of	37,499	18,421	49.1	48.6	49.6
E06000018	Nottingham	52,693	25,880	49.1	48.7	49.5
E06000043	Brighton and Hove	33,275	16,336	49.1	48.6	49.6
E06000030	Swindon	18,496	9,078	49.1	48.4	49.8
E06000023	Bristol, City of	58,017	28,467	49.1	48.7	49.5
E09000033	Westminster	48,724	23,723	48.7	48.2	49.1
E06000051	Shropshire	67,337	32,700	48.6	48.2	48.9
E08000028	Sandwell	39,552	19,164	48.5	48.0	48.9
E06000042	Milton Keynes	63,247	30,510	48.2	47.9	48.6
E08000036	Wakefield	61,543	29,680	48.2	47.8	48.6
E06000010	Kingston upon Hull, City of	17,074	8,219	48.1	47.4	48.9
E06000055	Bedford	31,728	15,205	47.9	47.4	48.5
E06000049	Cheshire East	52,794	25,264	47.9	47.4	48.3
E10000011	East Sussex	118,596	56,747	47.8	47.6	48.1
E08000009	Trafford	38,971	18,629	47.8	47.3	48.3
E06000044	Portsmouth	25,966	12,359	47.6	47.0	48.2
E06000059	Dorset	51,066	24,250	47.5	47.1	47.9
E08000023	South Tyneside	33,636	15,962	47.5	46.9	48.0
E10000030	Surrey	74,960	35,532	47.4	47.0	47.8
E06000015	Derby	62,407	29,315	47.0	46.6	47.4
E06000032	Luton	48,454	22,742	46.9	46.5	47.4
E08000008	Tameside	42,845	20,077	46.9	46.4	47.3
E10000008	Devon	105,836	49,495	46.8	46.5	47.1
E09000013	Hammersmith and Fulham	43,237	20,205	46.7	46.3	47.2
E09000007	Camden	44,662	20,798	46.6	46.1	47.0
E10000023	North Yorkshire	160,704	74,128	46.1	45.9	46.4
E09000004	Bexley	41,045	18,789	45.8	45.3	46.3
E08000003	Manchester	36,987	16,930	45.8	45.3	46.3
E10000028	Staffordshire	99,238	45,042	45.4	45.1	45.7
E08000013	St. Helens	35,045	15,868	45.3	44.8	45.8
E08000011	Knowsley	31,100	14,066	45.2	44.7	45.8
E06000058	Bournemouth, Christchurch and Poole	43,888	19,839	45.2	44.7	45.7
E06000020	Telford and Wrekin	34,384	15,444	44.9	44.4	45.4
E06000009	Blackpool	28,193	12,621	44.8	44.2	45.3
Unknown	Unknown	7,197	3,217	44.7	43.6	45.9
E10000002	Buckinghamshire	136,674	61,016	44.6	44.4	44.9

Patel R, *et al. BMJ Open* 2020; 10:e042963. doi: 10.1136/bmjopen-2020-042963

E10000032	West Sussex	90,033	40,022	44.5	44.1	44.8
E06000006	Halton	26,863	11,753	43.8	43.2	44.3
E06000052	Cornwall	48,099	20,877	43.4	43.0	43.8
E06000050	Cheshire West and Chester	40,408	17,537	43.4	42.9	43.9
E06000035	Medway	60,300	26,064	43.2	42.8	43.6
E10000020	Norfolk	161,582	69,173	42.8	42.6	43.1
E06000017	Rutland	6,741	2,862	42.5	41.3	43.6
E09000006	Bromley	75,672	31,841	42.1	41.7	42.4
E10000016	Kent	347,229	145,984	42.0	41.9	42.2
E09000008	Croydon	29,612	12,399	41.9	41.3	42.4
E09000011	Greenwich	32,488	13,547	41.7	41.2	42.2
E06000014	York	20,330	8,385	41.2	40.6	41.9
E08000027	Dudley	78,489	32,316	41.2	40.8	41.5
E06000026	Plymouth	28,855	11,707	40.6	40.0	41.1
E08000012	Liverpool	99,029	40,074	40.5	40.2	40.8
E10000018	Leicestershire	172,437	69,666	40.4	40.2	40.6
E08000024	Sunderland	47,131	18,370	39.0	38.5	39.4
E09000020	Kensington and Chelsea	35,607	13,811	38.8	38.3	39.3
E06000007	Warrington	48,004	18,287	38.1	37.7	38.5
E08000031	Wolverhampton	32,226	12,091	37.5	37.0	38.0
E08000010	Wigan	53,620	19,638	36.6	36.2	37.0
E09000021	Kingston upon Thames	32,087	11,529	35.9	35.4	36.5
E06000041	Wokingham	5,010	1,621	32.4	31.1	33.7
E08000037	Gateshead	49,663	14,497	29.2	28.8	29.6
E06000037	West Berkshire	16,235	4,376	27.0	26.3	27.6
E08000032	Bradford	82,669	20,791	25.1	24.9	25.4

Supplementary Table 10: Number of invitations recorded for attendees and non-attendees

Number of invitations	Attendees n(%)	Non-attendees n(%)
0	1,672,844 (32.8)	51,739 (1.1)
1	2,577,581 (50.5)	3,369,517 (73.4)
2	677,783 (13.3)	783,472 (17.1)
> 2	174,550 (3.4)	387,493 (8.4)
**TOTAL**	**5,102,758 (100.0)**	**4,592,221 (100.0)**

Patel R, *et al. BMJ Open* 2020; 10:e042963. doi: 10.1136/bmjopen-2020-042963

Supplementary Table 11: Invitations by financial year

Proportion of attendees and non-attendees with an invitation recorded

Year	Attendees with invitation	% attendees	Non-attendees with invitation	% non-attendees
2012/13	468,766	63.1	718,527	99.0
2013/14	619,559	64.3	824,429	98.9
2014/15	763,444	67.2	1,016,155	99.0
2015/16	790,731	69.2	999,178	98.7
2016/17	787,414	70.4	982,193	98.8
**TOTAL**	**3,429,914**	**67.2**	**4,540,482**	**98.9**

Supplementary Table 12: Completeness of risk factor measurement

Percentage of NHSHC attendees and non-attendees with recorded risk factor measurements (restricted to 15-month window around index date for attendees and unrestricted for non-attendees)

Group	CVD risk score	Body Mass Index	Physical Activity (GPPAQ)	Alcohol (Audit C)	Fasting glucose	HbA1C	Smoking Status	Cholesterol (HDL)	Cholesterol (total)	Diastolic BP	Systolic BP
Attendees	79.7%	96.3%	64.5%	38.3%	18.2%	36.6%	95.7%	87.2%	93.6%	95.7%	95.8%
Non-attendees	30.4%	55.0%	13.9%	16.7%	15.1%	37.5%	71.8%	47.3%	50.0%	76.3%	76.3%

Patel R, *et al. BMJ Open* 2020; 10:e042963. doi: 10.1136/bmjopen-2020-042963

Supplementary Table 13: Statin prescription rates

New statin (any dose) prescriptions among the subset (60.4%) of NHSHC attendees in whom medication data was available

Group	Attendees (n)	Prescribed a statin (n)	Proportion (%)
CVD score <10%	1,910,919	63,227	3.3
10-19.9%	532,046	83,279	15.7
≥20%	132,366	51,691	39.1
No CVD score	504,374	55,630	11.0
**Overall total**	**3,079,705**	**253,827**	**8.2**

Patel R, *et al. BMJ Open* 2020; 10:e042963. doi: 10.1136/bmjopen-2020-042963
