# Supplementary material for: Evaluation of the uptake and delivery of the NHS Health Check programme in England, using primary care data from 9.5 million people: a cross-sectional study
Source: BMJ Open. 2020 Nov 5;10(11):e042963. doi: 10.1136/bmjopen-2020-042963 (PMC7646358; doi:10.1136/bmjopen-2020-042963)
Supplement: Author's manuscript [file bmjopen-2020-042963.draft_revisions.pdf]

BMJ Open is committed to open peer review. As part of this commitment we make the peer review history of every article we publish publicly available.

When an article is published we post the peer reviewers' comments and the authors' responses online. We also post the versions of the paper that were used during peer review. These are the versions that the peer review comments apply to.

The versions of the paper that follow are the versions that were submitted during the peer review process. They are not the versions of record or the final published versions. They should not be cited or distributed as the published version of this manuscript.

BMJ Open is an open access journal and the full, final, typeset and author-corrected version of record of the manuscript is available on our site with no access controls, subscription charges or pay-per-view fees (http://bmjopen.bmj.com).

If you have any questions on BMJ Open's open peer review process please email info.bmjopen@bmj.com

BMJ Open

An evaluation of the uptake and delivery of the NHS Health Check Programme in England, using primary care data from 9.5 million people: A cross-sectional study

Journal:	*BMJ Open*
Manuscript ID	bmjopen-2020-042963
Article Type:	Original research
Date Submitted by the Author:	21-Jul-2020

Complete List of Authors:	Patel, Riyaz; University College London, Institute of Cardiovascular Science<br>Barnard, Sharmani; Public Health England<br>Thompson, Katherine; Public Health England<br>Lagord, Catherine; Public Health England<br>Clegg, Emma; Public Health England<br>Worrall, Robert; NHS Digital<br>Evans, Tim; Public Health England<br>Carter, Slade; Public Health England<br>Flowers, Julian; Public Health England<br>Roberts, Dave; NHS Digital<br>Nuttall, Michaela; Public Health England<br>Samani, Nilesh; University of Leicester, Department of Cardiovascular Sciences<br>Robson, John; Queen Mary University of London, Centre for Primary Care and Public Health<br>Kearney, Matt; UCL Partners<br>Deanfield, John; University College London, Institute of Cardiovascular Science<br>Waterall, Jamie; Public Health England

Keywords:	VASCULAR MEDICINE, PUBLIC HEALTH, PREVENTIVE MEDICINE

SCHOLARONE™
Manuscripts

I, the Submitting Author has the right to grant and does grant on behalf of all authors of the Work (as defined in the below author licence), an exclusive licence and/or a non-exclusive licence for contributions from authors who are: i) UK Crown employees; ii) where BMJ has agreed a CC-BY licence shall apply, and/or iii) in accordance with the terms applicable for US Federal Government officers or employees acting as part of their official duties; on a worldwide, perpetual, irrevocable, royalty-free basis to BMJ Publishing Group Ltd ("BMJ") its licensees and where the relevant Journal is co-owned by BMJ to the co-owners of the Journal, to publish the Work in this journal and any other BMJ products and to exploit all rights, as set out in our licence.

The Submitting Author accepts and understands that any supply made under these terms is made by BMJ to the Submitting Author unless you are acting as an employee on behalf of your employer or a postgraduate student of an affiliated institution which is paying any applicable article publishing charge ("APC") for Open Access articles. Where the Submitting Author wishes to make the Work available on an Open Access basis (and intends to pay the relevant APC), the terms of reuse of such Open Access shall be governed by a Creative Commons licence – details of these licences and which Creative Commons licence will apply to this Work are set out in our licence referred to above.

Other than as permitted in any relevant BMJ Author's Self Archiving Policies, I confirm this Work has not been accepted for publication elsewhere, is not being considered for publication elsewhere and does not duplicate material already published. I confirm all authors consent to publication of this Work and authorise the granting of this licence.

For peer review only

Title: An evaluation of the uptake and delivery of the NHS Health Check Programme in England, using primary care data from 9.5 million people: A cross-sectional study

Running Title: NHS Health Check Programme

Author Block:

Riyaz S. Patel,[1, 2,]* BHF Clinical Intermediate Fellow, Sharmani Barnard,[3,]* Statistician, Katherine Thompson,[3] Head of CVD Prevention programme, Catherine Lagord,[3] Analyst, Emma Clegg,[3] Analyst, Rob Worrall, [4] Analyst, Tim Evans,[3] Analyst, Slade Carter,[3] Programme Manager, Julian Flowers,[3] Head of Public Health Data Science, Dave Roberts,[4] Head of Primary Care Information, NHS Digital, Data Extract Advisory Committee (DEAC),[#] Michaela Nuttall,[3] Clinical Adviser, Nilesh J Samani,[5] Professor of Cardiology, John Robson,[6]Reader in Primary Care , Matt Kearney,[7] GP and Deputy Managing Director UCL Partners Academic Health Science Network, John Deanfield,[1,3,]* Professor of Cardiology, Jamie Waterall [3,]* Deputy Chief Nurse

*These authors contributed equally; [#]DEAC membership included at the end of the paper

Affiliations:

1. Institute of Cardiovascular Sciences, University College London, London, UK,
2. Bart's Heart Centre, St Bartholomew's Hospital, West Smithfield, London, UK
3. Public Health England, Wellington House, 133-155 Waterloo Road, London, UK
4. NHS Digital, 1 Trevelyan Square, Boar Lane, Leeds, UK
5. Department of Cardiovascular Sciences University of Leicester and NIHR Leicester Biomedical Research Centre, Glenfield Hospital, Leicester, UK
6. Centre for Primary Care and Public Health, Queen Mary University of London, London
7. UCL Partners, 3rd Floor, 170 Tottenham Court Road, London, UK

Corresponding Author:

Dr Riyaz Patel, https://orcid.org/0000-0003-4603-2393

222 Euston Rd, Institute of Cardiovascular Sciences, University College London, London, NW1 2DA

Email: Riyaz.patel@ucl.ac.uk Telephone: +44 (0) 20 3549 5332

Word Count:

Abstract: 292; Main Text: 4096

For peer review only - http://bmjopen.bmj.com/site/about/guidelines.xhtml

Abstract:

Objectives: To describe the uptake and outputs of the NHS Health Check (NHSHC) programme in England.

Design: Observational study

Setting: National primary care data extracted directly by NHS Digital from 90% of General Practices (GP) in England.

Participants: Individuals aged 40-74 years, invited to or completing a NHSHC between 2012 and 2017, defined using primary care Read codes.

Intervention: The NHSHC, a structured assessment of non-communicable disease risk factors and 10-year cardiovascular disease (CVD) risk, with recommendations for behavioural change support and therapeutic interventions.

Results: During the 5-year cycle, 9,694,979 individuals were offered an NHSHC and 52.6% took up the offer. There was geographical variation in uptake between local authorities across England ranging from 25.1% to 84.7%. Invitation methods changed over time to incorporate greater digitalisation, opportunistic delivery and delivery by third party providers.

The population offered an NHSHC resembled the English population in ethnicity and deprivation characteristics. Attendees were more likely to be older and female, but were similar in terms of ethnicity or deprivation, compared to non-attendees. Among attendees, risk factor prevalence reflected population survey estimates for England, with 20.6% having a 10-year CVD risk ≥10%, of which 20.3% were prescribed a statin. Advice, information and referrals were coded as delivered to over 2.5 million individuals identified to have risk factors.

Conclusion: This national analysis of the NHSHC programme using primary care data from over 9.5M individuals offered a check, reveals an uptake rate of over 50% and no significant evidence of inequity by ethnicity or deprivation. To maximise the anticipated value of the NHSHC, we suggest continued action is needed to invite more eligible people for a check, reduce geographical variation in uptake, prioritise engagement with non-attendees, and promote greater use of evidence-based interventions especially where risk is identified.

Keywords: Cardiovascular Disease Prevention; NHS Health Checks; Cardiovascular Risk; Public Health

For peer review only - http://bmjopen.bmj.com/site/about/guidelines.xhtml

Strengths and Limitations:

- A comprehensive national level snapshot of NHS Health Check (NHSHC) programme, derived from primary care records, and which underpins the recently released NHSHC data dashboard
- Academic and public health collaboration with full access to half a billion records for over 9.5M people offered an NHSHC between 2012-2017
- This first data analysis reports on elements relating to uptake, implementation, process and delivery of NHSHCs, the sociodemographic and risk factor profile of both those who did and did not attend a check and subsequent use of risk modifying interventions
- The study examines individuals who were coded as being invited or having received an NHSHC through Read codes, and as such does not include those who may have been eligible but not yet invited
- Future planned analyses will report on the detailed information collected on risk factors, opportunities for CVD prevention and the impact of interventions made during NHSHC encounters

For peer review only - http://bmjopen.bmj.com/site/about/guidelines.xhtml

Introduction

Cardiovascular disease (CVD) remains a major public health priority in England.[1] To address this the Government introduced an ambitious programme of vascular checks in 2009, for people aged 40-74, delivered by England's National Health Service (NHS).[2] NHS Health Checks (NHSHC) sought to address the key risk factors driving the health and economic burden from vascular disease,[3] with early modelling suggesting that each year NHSHCs would prevent 9,500 heart attacks and strokes, 4,000 new cases of diabetes and identify at least 25,000 people with existing undiagnosed diabetes or kidney disease before they developed complications.[2 4] Furthermore, with the same vascular risk factors increasingly recognised as contributing to other conditions like dementia, preventable cancers, and liver disease,[3] the programme has assumed an even greater importance in the prevention of non-communicable diseases.[5 6 7]

Over a decade on, the NHSHC, is now an embedded systematic and nationwide detailed risk assessment, awareness and management programme in England. Since 2013, following legislation, local authorities have a statutory obligation to make provision for all eligible people to have an NHSHC every five years.[8] However, concerns have been raised that delivery and practical implementation of such a programme presents a paradoxical risk of increasing health inequality if implemented in a way which does not systematically prioritise equity of access, outputs and outcomes. Furthermore, the absence of convincing randomised clinical trial evidence about the effectiveness of such programmes, has further prompted ongoing scrutiny and questions around its delivery, uptake, impact and cost-effectiveness.[9]

In response, the number of studies evaluating the delivery and impact of the NHSHC continue to grow but have shown variable results.[10] This may be a result of heterogeneity in programme delivery, small sample sizes, use of national data before NHSHCs were passed into law, or variation in local coding practices. In addition, some studies have drawn conclusions from analyses of the Clinical Practice Research Datalink (CPRD), or QResearch databases,[11] which although a representative and important primary care research resource, are limited by being restricted to volunteer practices utilising specific electronic health record systems with some under-representation in Northern England.[11 12]

To overcome some of these difficulties and provide a contemporaneous overview of the NHSHC programme in England, we sought to analyse the largest NHSHC national primary care dataset to be extracted to date, drawing on data for almost ten million individuals and half a billion records, specifically extracted for this purpose and one which underpins the recently released NHSHC data dashboard.[13] A series of reports will examine the delivery of the programme, prevention opportunities

For peer review only - http://bmjopen.bmj.com/site/about/guidelines.xhtml

identified and the impact of the NHSHC. In this first paper, we use these data to describe the uptake and outputs of the programme, elements relating to its implementation, process and delivery as well as the sociodemographic and risk factor profile of those who were offered a check and subsequently did or did not attend for one.

Methods

Study Setting

Public Health England (PHE) is responsible for national oversight and implementation support of the NHSHC programme. PHE worked with NHS Digital (NHSD) to develop business rules for a data extract of all NHSHC coding activity to allow England wide monitoring of the NHSHC.[14] A data extract advisory committee (DEAC) was set up to guide use of the data extract. Full details of the scope and composition of the committee are available online.[15]

Study Design

We conducted a retrospective descriptive cross-sectional study of all individuals who were offered an NHSHC, using individual-level participant data. We describe the data extraction before defining the study population. The study design and report conform to RECORD recommendations for reporting of observational studies using routinely collected data.[16]

Data Extraction & Criteria

Data was extracted from 6,524 (90%) of the 7,216 General Practices participating in the General Practice Data Extraction Service (GPES),[17] after excluding individuals who had opted out of their data being used for purposes other than direct patient care. [18]

The inclusion criteria for the data extract, was a primary care Read code for any one of the following NHSHC activities: invitation, completion, non-attendance, inappropriate, commenced or declined (prior to 1st April 2018). Full details of the Read codes used for defining NHSHC activity is available in **Supplementary Table 1**.

The data extracted for each individual included socio-demographic characteristics, risk factors for cardiovascular disease, diagnostic tests, and interventions including advice and referrals. CVD diagnoses and medication data were also extracted from three out of the four GP clinical IT systems providers, corresponding to 60% of practices. Data extraction for all variables were restricted to time

For peer review only - http://bmjopen.bmj.com/site/about/guidelines.xhtml

For peer review only

windows around the individual's contact with the NHSHC programme as specified in the business rules for extraction, listed in **Supplementary Table 2**.

At the time of extraction in 2018, the business rules limited the upper age limit to 75 years for each year. As a result, due to the rolling nature of the programme, this resulted in missing data for the 70-74 age group, most of whom turned 75 during the 5-year cycle. Thus, the maximum age of patients in the extract is 69 for the financial year 2012/13, compared to 73 in 2016/17. The final extraction consisted of 12,151,896 patient records with NHSHC activity coding recorded up until 31st March 2018. Data management and data cleaning details are provided in **Supplementary Methods** and **Supplementary Table 3**.

Study Population

NHSHCs are offered to individuals aged 40-74 years and without any of the following conditions: hypertension, diabetes mellitus, familial hypercholesterolaemia, coronary heart disease, heart failure, atrial fibrillation, stroke or transient ischaemic attack, peripheral arterial disease, chronic kidney disease and those already on statins or known to have a 10-year CVD risk of ≥ 20%.[5]

The study population for this analysis was derived from the data extract described above for any NHSHC coded activity. From this group, individuals (1) with NHSHC activity coded outside the study window, (2) aged <40 years at the time of activity, and (3) coded by the GP as inappropriate for an NHSHC were then additionally excluded. The final study population thus included only those people offered an NHSHC (invited or completed). **Figure 1** presents the study extract and population flow chart.

Definitions and Study Variables

Individuals were categorised as either NHSHC attendees if they had a Read code for a completed check within the 5-year period, or a non-attendee if they did not. Uptake of the programme was defined as the proportion of the total study population who attended.

An index date was generated from the date of an individual's primary NHSHC activity to identify age and the most relevant risk factor measurements for each patient. Risk factor and clinical measurements were selected for analysis if they occurred on the index date. Otherwise we took the closest recording within pre-defined time windows set by the DEAC. A full list of variables, Read codes used to define variables, time windows and coding algorithms is available in **Supplementary Table 4**.

For peer review only - http://bmjopen.bmj.com/site/about/guidelines.xhtml

Further details on study variable definitions and thresholds are provided in **Supplementary Methods** and **Supplementary Tables 4-8.**

Data Presentation

Statistical tests were not used for comparison because the amount of missing data between groups varies, thereby preventing meaningful comparisons and the large size of the study population permits the identification of very small differences between groups. Instead, we highlighted the size of differences between groups and interpreted it in relation to the missing data. Where appropriate, we presented data for attendees and non-attendees. Data for uptake, invitation type and third-party provider is presented by financial year, to describe changes over time. Data on uptake is also presented by local authority for geographical comparisons. To minimise bias, we include missing data details in all tables and figures.

Patient and Public Involvement

PHE developed an information notice for patients, including an easy read version, explaining how their personal data would be used and the purpose of the research project. Membership of the Data Extract Advisory Committee overseeing the use of the NHS Health Check dataset, including the development of this study, its design and outcomes, includes a patient representative. Study results will not be disseminated to individuals whose data is used but the collective analysis presented here will be shared publicly once published.

Ethical Approval

A Direction from the Secretary of State for Health and Social Care instructed NHS Digital with the legal requirement to carry out the NHSHC data extract.[19] This study was subject to an internal review by the Research Support and Governance Office in PHE to ensure that it was fully compliant with the UK Policy Framework for Health and Social Care Research (2017) and with all other current regulatory requirements. The review also covered all ethical considerations. No ethical issues were identified and thus review by an ethics committee was not required (Personal communication between Katherine Thomson & PHE Research Support Governance Office, 2019).

For peer review only - http://bmjopen.bmj.com/site/about/guidelines.xhtml

Results

NHSHC Uptake

Overall Uptake by Year

Between 1st April 2012 and 31st March 2017, 9,694,979 individuals aged 40 to 74 years were offered an NHSHC in England. Of these 5,102,758 (52.6%) completed a check. Uptake by financial year is presented in **Table 1**. Uptake remained > 50% throughout the five years of programme delivery. The number of individuals offered a NHSHC increased from just under 1.5M in 2012/13, to 1.8M the year after, plateauing thereafter at approximately 2.1M each year after that, **Table 1**.

Geographical variation in uptake of offers

Across England, uptake rates varied by region, as presented in **Figure 2A.** The highest uptake of offers over the five-year cycle was in Hampshire (84.7%) and the lowest in Bradford (25.1%). Data for uptake by upper tier local authority (UTLA) is available in **Supplementary Table 9**. Variation in uptake in London is shown in **Figure 2B.** Central and north London local authorities had higher rates of uptake, with lower rates in the south east.

Process and Delivery

Invitation Frequency

Of the 9,694,979 individuals in the study population with codes for NHSHC activity, 7,970,396 (82.2%) had a record of at least one NHSHC invitation. **Supplementary Table 10** presents the number of recorded invitations for attendees and non-attendees (recording by each financial year is available in **Supplementary Table 11**).

Among the 5,102,758 attendees, almost a third (32.8%), had no invitation code recorded but still had a completed NHSHC recorded. The remaining two thirds (3,429,914) had an invitation recorded, with 50.5% having one invitation, and 16.7% two or more. Among these attendees coded as invited, 590,869 (17.2%) received an invitation on the same date as the NHSHC and were thus assumed to be opportunistic rather than planned. Among those with an invitation in advance of the NHSHC (82.8%; n= 2,839,045), the median number of days between recording of their first invitation and a completed NHSHC was 42 (IQR 21, 90) days.

Among non-attendees, 98.9% had a formal invitation record, with a quarter (25.5%) having two or more invitations. The remaining 1.1% of non-attendees had Read codes for declining or not attending a check, **Supplementary Table 1**.

For peer review only - http://bmjopen.bmj.com/site/about/guidelines.xhtml

Invitation Type

Among both attendees and non-attendees, the most common invitation type was a letter, however other forms of invitations, including text messaging, increased with each year of the programme. **Supplementary Figure 1** presents the type of invitation by financial year among attendees and non-attendees.

Delivery

Among all attendees within the five-year timeframe, 3.0% had a clinical code to indicate that their NHSHC was completed by a third party. This increased gradually from 1.2% in the first year to 4.1% in the final year.

<u>Characteristics of Invitees</u>

Socio-Demographic Characteristics

Table 2 presents the socio-demographic characteristics of the study population and the characteristics of the general population according to ONS modelled estimates. The population offered an NHSHC was representative of the general population of people aged 40-74 years in terms of sex and deprivation index although they were younger relative to the age distribution of the general population (age <55: 62.2% v 49.7%). Those who were offered an NHSHC also closely resembled the ethnic makeup of the general population for most ethnicities, except for people self-reporting as white or black Caribbean who appeared underrepresented, although 16.7% of data for ethnicity was missing.

Attendees differed from non-attendees. More attendees were female (54.7%) compared to non-attendees (47.5%; general population 50.9%). There were also notable differences by age. Most attendees were < 55 years as they constituted the largest group of eligible people, but individuals ≥55 years had higher rates of attendance after invitation. For ethnic group comparisons, a large proportion of missing data for non-attendees (27.8%) compared to attendees (6.8%) limits interpretation, but where data were available and compared to the general population, ethnic minority groups appeared to be better represented among attendees than non-attendees, **Table 2**.

Deprivation indices indicate few differences between attendees and non-attendees, except at the extreme ends of the index of multiple deprivation (IMD) spectrum, where there were slightly more attendees from the most affluent areas (Decile 10: 11.0% v 10.0%) and slightly less attendees from the most deprived areas (Decile 1: 8.2% vs 9.4%). Finally, although the numbers were small, there was

For peer review only - http://bmjopen.bmj.com/site/about/guidelines.xhtml

no evidence to indicate that people with severe mental illness, physical or cognitive disability were under-represented among attendees, **Table 2**.

Risk Factors

Overall, completeness of data for common risk factors measurements including systolic blood pressure (BP) (95.7%), smoking (95.7%), BMI (96.3%) and total cholesterol (93.6%) was high in attendees, in contrast to recording of physical activity (64.5%), blood glucose (49.9%) and (38.3%). A CVD risk score was formally recorded for 79.7% of attendees (**Figure 3** and **Supplementary Table 12**). Family history data was only recorded where a positive finding was present, making it difficult to estimate how much data was missing or was assessed and was negative. Completeness of all risk factors was lower among non-attendees.

Figure 4 shows the proportion of individuals identified as having each CVD risk factor among attendees and non-attendees. Among attendees, where missingness was low, we identified 24.5% with hypertension, while 23.8% were obese and 16% were current smokers. Among the 80% in whom a 10-year CVD risk score had been estimated, 20.4% were found to be at high risk with a score of ≥ 10%.

Advice, Referrals and Interventions

Advice, information and referral for an intervention following an NHSHC was recorded almost six million times for all attendees, and more than 2.5 million times for individuals with elevated CVD risk factors, **Table 3.** Among all attendees, 16.0% were coded to have received general lifestyle and behavioural advice, just over a fifth were given formal advice on diet, and almost a third on physical activity. Among those whose alcohol use puts them above low risk, more than a third were directed to alcohol treatment services. Almost half of all current smokers were directed to smoking cessation services and 19.6% of those who had a BMI ≥ 30 were directed to weight loss and obesity services.

Statin Prescriptions

Information on a new statin prescription, occurring on or after NHSHC completion, was available for 60.4% of all attendees (n=3,079,705, see Methods). Overall a statin was prescribed for 8.2% of these attendees. Dividing this group by CVD risk, revealed that a statin was prescribed in 20.3% of those with a 10-year CVD risk score ≥ 10% and in 39.1% of those with a CVD risk score of ≥20%. Among the 1,910,919 individuals with a CVD risk score <10%, 3.3% received a new statin prescription, while in the remaining 504,374 with no CVD risk score recorded, 11.0% were prescribed a statin. **Supplementary Table 13.**

For peer review only - http://bmjopen.bmj.com/site/about/guidelines.xhtml

Assuming similar rates of statin prescription nationally, we estimate that of the 5,102,785 attendees in this study, up to 418,000 may have received a new statin prescription, with over half of these (n~213,000) prescribed to those identified at the NHSHC visit as being at >10% risk of CVD events.

Discussion

In the largest nationwide study of the NHS Health Check programme, using primary care data, we find that the checks been offered to over 9.5M people during a 5-year cycle up to 2017, with 52% of people taking up the offer. While we noted geographical variation in uptake rates, and an age and sex bias for attendance, we found little evidence of inequality in who was offered or who received an NHSHC by ethnicity or deprivation indices. Where an NHSHC was delivered, risk factors were identified at a similar rate to population estimates, with advice and referrals offered over 2.5M times to those with risk factors, along with 20% of those at highest risk receiving a new statin prescription as per guidelines. These insights into the evolving process and delivery of the NHSHC programme will support efforts to further enhance the value of the programme, especially for improving uptake rates, targeting those at greatest risk and maximising the use of available NCD & CVD risk reduction interventions.

Our key finding of a 52% uptake rate is slightly higher than previous studies, reporting around 48%.[10] This may be due to the larger, more nationally representative and contemporary data to which we had access, supported by the finding that uptake rates have steadily increased since 2012. Furthermore, we also found wide geographical variation, across the country and in London, possibly due to differing coding practices or invitation methods, which could skew findings from smaller studies or explain discordance with other reports of NHSHC activity.[20] However, an important difference that precludes direct comparison with other studies reporting on NHSHC reach is that our study was restricted to people who had an NHSHC code in their GP records, indicating either an invitation or completion of a check. As such we were unable to quantify coverage of the programme, i.e. how many eligible people were offered a check. Estimates from PHE, based on Office for National Statistics data minus the estimated number of people on existing disease registers suggests an eligible population of ~15.5 million.[20] Using this number and based on 5.1M having had a check we estimate that a further 6.5M in the same 5 year cycle would need to complete an NHSHC to achieve the original programme aspiration of 75% coverage.[4 8]

Some NHSHC providers have raised concerns that the programme may paradoxically increase health inequality by only attracting the worried well with more affluent and white people.[21] Reassuringly the

For peer review only - http://bmjopen.bmj.com/site/about/guidelines.xhtml

data do not show gross differences in the offering or uptake of the programme. Firstly, those who were offered a NHSHC closely resemble the population of England, as measured through census data, with no differences by sex, ethnicity or deprivation indices. They were slightly younger overall, but this is likely because eligibility for an NHSHC falls with comorbidities which are frequently age related.[5] Secondly, although missing data on ethnicity limits definitive conclusions, ethnic minorities such as those from South Asia were equally if not more represented as reported by others.[22 23] Furthermore, while there were small differences at the extremes of deprivation deciles, overall there was no gross bias towards greater attendance by increasing affluence and previous mixed findings are likely due to regional variation, [22-24] while the similar uptake rates in those with physical disability or serious mental illness also indicates the programme is equitably delivered. There was however a notable bias towards more females and older people attending for a NHSHC compared to non-attendees, a finding also observed by others.[10 11 22 23]

Of note, despite older people being more likely to attend than not attend after having an offer of a NHSHC, proportionally 57% of all attendees were <55 years, higher than reports from other national evaluations of the programme.[11] This could be because our data was limited for the age 70-74 group or that more older people are excluded having been identified with comorbidities earlier in the programme cycle when these other studies reported. However, it may also indicate that younger people are motivated to understand their CVD risk and engage with care providers to address their longer term and lifetime risk, a finding we previously observed with the use of digital risk assessment tool.[25] The potential benefits of this earlier engagement with CVD risk, will need to be evaluated over the longer term.

An important benefit of the NHSHC programme has been improvements in risk factor and behaviour data recording, which can guide patient interventions and inform regional resource priorities. For core items such as smoking, data completeness was as high as 96%, while for alcohol and physical activity (measures which are contractually required as part of the NHSHC but not needed to calculate a person's 10-year CVD risk) was close to 65%. This contrasts with the high degree of missing data among non-attendees. Where risk factors, were recorded, they reveal that prevalence in attendees is close to those in the wider UK population.[3 26] Overall, a fifth of all attendees were calculated to have a 10-year CVD risk score of ≥10%, the current threshold set by NICE to consider preventative interventions such as statin prescription.[27] Indeed, we found 20% of this population was initiated on a statin following the NHSHC. This figure was even higher at nearly 40% for those with a 10-year CVD risk score of ≥20%, an older NICE threshold for statin prescription. This is an encouraging finding, being higher than in earlier studies and approaching the national ambition of 45% for statin use in this very

For peer review only - http://bmjopen.bmj.com/site/about/guidelines.xhtml

high risk group.[11 28] Our data also suggest that the NHSHC encounter prompted relevant non-statin interventions with over 2.5M people with risk factors being coded as having received advice, information or referrals. We note however that these figures may be an underestimate being entirely dependent on coding practices and availability of services by region.

Limitations:

Despite being the largest national evaluation of the NHSHC programme, our study has some important limitations. Firstly, our data was restricted to people with an NHSHC activity code, and thus we were unable to quantify the full eligible population to determine coverage and the gap in programme reach. Although this is an aspiration for future analyses, it will require access to GP records for much of the population, raising important data governance and handling challenges. Secondly, we had substantial missing data, especially for the non-attendees, limiting our ability to make robust conclusions about differences in characteristics and risk between these groups. Thirdly, important information on those >70 years was limited due to a business rule that led to loss of older people once they turned 75 for each year of the data extract. However, the proportionally smaller number of older people eligible for an NHSHC means our results are unlikely to have been impacted significantly. Fourthly, prescription data was only available from 60% of practices. The estimate for statin prescriptions derived from the available data however is likely valid and representative. Finally, we used a Read code to identify if an NHSHC took place. This, of course does not provide any indication as to the extent or quality of the conversations around risk or the suitability of information given, upon which the full impact and value of an NHSHC is likely to depend.

Clinical Implications:

This analysis provides a national level overview of the NHSHC programme, against which local authorities and health care providers can benchmark local achievements. Used with the NHS Digital dashboard, this will enable local CVD risk strategies to be developed, to increase the invitation of eligible individuals not yet invited for an NHSHC, as well as targeting those who still do not attend even after invitation.[13] Importantly, we show that a national prevention programme to tackle NCDs is possible and population health can be targeted through routine health care. It represents a systematic approach to switching the conversation from illness to preventing disease and appears to have good engagement from the public so far. From the data, we observe that in England there remains a major challenge for reducing risk factors that impact multiple long-term chronic conditions. The programme appears to have been successful at promoting advice and guideline-based interventions. The extent

For peer review only - http://bmjopen.bmj.com/site/about/guidelines.xhtml

of how well and broadly this has been achieved, along with the impact of such interventions will follow with further analysis of this large NHSHC dataset.

Conclusion:

In this large-scale analysis of the NHSHC programme using national primary care data, we found that in recent years over half of all people offered a check have completed one. Although there was substantial variation between local authorities in uptake rates, we found little or no evidence of inequity in invitation processes or uptake. Furthermore, the programme has identified a high burden of risk among attendees, with correspondingly encouraging levels of guideline driven advice, referrals and statin prescriptions for the primary prevention of CVD. However, to achieve fully the anticipated benefits of the NHSHC programme, we highlight a need for continued efforts to invite more of the eligible population for an NHSHC, reduce geographical variation in uptake of offers, prioritise those who are not attending and to maximise the use of evidence-based interventions to support risk reduction.

For peer review only

Statements

Funding

RSP (FS/14/76/30933) and JD (BHF chair) were funded by the BHF. Data extraction and analysis were funded by PHE.

Acknowledgments

We would like to thank colleagues from PHE and NHS Digital who supported this work. We would also like to thank the patient and public representatives involved with this work, for their input.

Disclosures/ Competing Interests

All authors have completed the ICMJE uniform disclosure form at www.icmje.org/coi_disclosure.pdf and declare: no support from any organisation for the submitted work; RSP has received speaker fees and honoraria from Amgen, Sanofi and Bayer and research grant funding from Regeneron for CVD prevention and cholesterol management; no other relationships or activities that could appear to have influenced the submitted work.

Copyright:

The Corresponding Author has the right to grant on behalf of all authors and does grant on behalf of all authors, a worldwide licence to the Publishers and its licensees in perpetuity, in all forms, formats and media (whether known now or created in the future), to i) publish, reproduce, distribute, display and store the Contribution, ii) translate the Contribution into other languages, create adaptations, reprints, include within collections and create summaries, extracts and/or, abstracts of the Contribution, iii) create any other derivative work(s) based on the Contribution, iv) to exploit all subsidiary rights in the Contribution, v) the inclusion of electronic links from the Contribution to third party material where-ever it may be located; and, vi) licence any third party to do any or all of the above.

Transparency Declaration:

The guarantors (RP, SB, KT and CL) affirm that this manuscript is an honest, accurate, and transparent account of the study being reported; that no important aspects of the study have been omitted; and that any discrepancies from the study as planned have been explained.

Data Sharing Statement

For peer review only - http://bmjopen.bmj.com/site/about/guidelines.xhtml

The legal basis for the data extract was a Secretary of State for Health and Social Care Direction. With DEAC approval PHE and NHS Digital have set up a process for dealing with information requests relating to the pseudonymised primary care data used in this paper. The purpose for using this data must be for the scope of work relating to the evaluation of the NHS Health Check in line with the requirements of the Direction.

Author Contributions

All authors contributed to conception of the study, study design, overall analysis plan and critically reviewed the final manuscript. Specifically in addition, RSP and KT contributed to the statistical analysis plan, review of results and drafted and revised the final paper; SB, CL, EC, TE and RW obtained and analysed all data and contributed to drafting of the final manuscript; SC, JF and DR supported data extraction for the analysis and review of the final manuscript; MN, NS, JR critically reviewed and edited the paper; MK, JED, JW conceived the study; contributed to the analysis plan and critically reviewed the final manuscript.

For peer review only - http://bmjopen.bmj.com/site/about/guidelines.xhtml

Data Extract Advisory Committee for NHS Health Check data extraction (DEAC): membership as of April 2020

John Deanfield (Co-Chair), Senior advisor to Public Health England on cardiovascular disease prevention & UCL professor of cardiology; Matt Kearney (Co-Chair), Programme Director, UCL Partners Academic Health Science Network, GP; Andrew Hughes, Heart Intelligence, National Cardiovascular Intelligence Network, PHE; Bob Ruane, Patient representative; Catherine Lagord (secretariat), Analyst, CVD prevention team, PHE; Dave Roberts, Head of primary care information, NHS Digital; Emma Brezan, Senior Public Health Manager, Royal Borough of Greenwich; Ifeoma Onyia, Consultant in public health, Halton Borough Council; Jamie Waterall, National Lead for Cardiovascular Disease Prevention, PHE; John Robson, Clinical Reader in Primary Care Research & Development Queen Mary University of London; Julian Flowers, Head of public health data science, PHE; John Newton, Director of Health Improvement, PHE; Kate Cheema, Director of Health Intelligence, British Heart Foundation; Katherine Thompson, Head of Cardiovascular Disease Prevention, PHE; Kathryn Salt, Principal data manager, primary care domain, NHS Digital, Lorraine Oldridge, National lead, National Cardiovascular Intelligence Network, PHE; Michaela Nuttall, Deputy National Lead, CVD prevention team, PHE; Mohammed Vaqar, Health and Wellbeing Officer, CVD, PHE West Midlands; Nick Wareham, Director of the MRC Epidemiology Unit, University of Cambridge; Nilesh Samani, Professor of cardiology at University of Leicester, medical director, British Heart Foundation; Paul Cundy, GP and chair of the GPC IT subcommittee; Peter Green, Clinical Chair NHS Medway Clinical Commissioning Group; Peter Kelly, Centre director, PHE North East; Phil Koczan, Royal College of General Practitioners representative, Riyaz Patel, BHF Senior Lecturer at UCL and Consultant Cardiologist at UCLH and Barts Health NHS Trusts; Rob Aldridge, Associate Professor, Institute of Health Informatics, UCL; Robert Danks, Principal Information Analyst, Primary Care Domain, NHS Digital; Rob Worrall, Senior information analyst, primary care domain, NHS Digital; Sharmani Barnard, Statistician, PHE; Tim Evans, Stroke Intelligence, National Cardiovascular Intelligence Network, PHE; Zain Chaudhry, NHS England and NHS Improvement;

For peer review only - http://bmjopen.bmj.com/site/about/guidelines.xhtml

Figure Legends

Figure 1: Study extract and study population flow chart. The study population inclusion dates (1st April 2012 to 31st March 2017) reflect a snapshot of the five-year rolling programme from April 2012, when all trusts commissioning primary care in England had implemented the programme.

*NHS Health Check activity refers to any interaction that a patient may have had with the NHS Health Check programme. This includes if a patient was invited to, commenced, completed, declined, did not attend, or was inappropriate for, the NHS Health Check. More details are provided in Supplementary Table 1

Figure 2: Variation in NHSHC uptake across (A) England and (B) London. Uptake rates shown as % of people taking up an offer of a check, between 2012/3 to 2016/17, by Upper Tier Local Authority of the individuals' usual residence

Figure 3: Completion of risk factor measurements for attendees and non-attendees (2012/13 - 2016/17). Proportion of available and missing data for each risk factor related measurements are shown here. Note these are available measurements within the time frame of the data extract (see Supplementary Methods). Family history not shown as coded only as yes with unknown negative/missing data.

Figure 4: Proportion of attendees and non-attendees with common CVD risk factors. Definitions as per Supplementary Table 6 and include: High cholesterol = total cholesterol >5mmol/L or cholesterol ratio >4; High blood pressure = systolic ≥140 or diastolic pressure ≥90mmHg; Obesity = BMI≥30kg/m^2; Alcohol > low risk = AUDIT C score ≥8; Low physical activity = GPPAQ moderate inactive or inactive; Possible Diabetes = HbA1c ≥48mmol/mol or FBG>7mmol/L; Current Smoker = current smoking; High CVD Risk score = 10 year CVD risk score ≥10%. *Family history is predominantly only recorded if present so accurate information on its absence is unavailable.

For peer review only - http://bmjopen.bmj.com/site/about/guidelines.xhtml

REFERENCES

1. Global Burden of Diseases Causes of Death Collaborators. Global, regional, and national age-sex-specific mortality for 282 causes of death in 195 countries and territories, 1980-2017: a systematic analysis for the Global Burden of Disease Study 2017. *Lancet* 2018;392(10159):1736-88. doi: 10.1016/S0140-6736(18)32203-7
2. Department of Health. Putting Prevention First: Vascular checks, risk assessment and management 2008 [Available from: https://www.healthcheck.nhs.uk/seecmsfile/?id=1302 accessed December 2019.
3. Global Burden of Diseases Risk Factor Collaborators. Global, regional, and national comparative risk assessment of 84 behavioural, environmental and occupational, and metabolic risks or clusters of risks for 195 countries and territories, 1990-2017: a systematic analysis for the Global Burden of Disease Study 2017. *Lancet* 2018;392(10159):1923-94. doi: 10.1016/S0140-6736(18)32225-6
4. Department of Health. Economic Modelling for Vascular Checks 2009 [Available from: www.healthcheck.nhs.uk/document.php?o=225 accessed February 2020.
5. Public Health England. NHS Health Check Best Practice Guidance 2019 [updated October 2019. Available from: https://www.healthcheck.nhs.uk/seecmsfile/?id=1474 accessed February 2020.
6. NHS. NHS Long Term Plan 2018 [updated August 2019. Available from: https://www.england.nhs.uk/long-term-plan/ accessed February 2020.
7. Department for Health and Social Care. Advancing Our Health: Prevention in the 2020's Online2019 [Available from: https://www.gov.uk/government/consultations/advancing-our-health-prevention-in-the-2020s/advancing-our-health-prevention-in-the-2020s-consultation-document accessed March 2020.
8. Department of Health and Social Care. The Local Authorities (Public Health Functions and Entry to Premises by Local Healthwatch Representatives) Regulations London2013 [Available from: http://www.legislation.gov.uk/uksi/2013/351/regulation/4/made accessed December 2019.
9. Capewell S, McCartney M, Holland W. NHS Health Checks--a naked emperor? *J Public Health (Oxf)* 2015;37(2):187-92. doi: 10.1093/pubmed/fdv063
10. Martin A, Saunders CL, Harte E, et al. Delivery and impact of the NHS Health Check in the first 8 years: a systematic review. *Br J Gen Pract* 2018;68(672):e449-e59. doi: 10.3399/bjgp18X697649
11. Robson J, Dostal I, Sheikh A, et al. The NHS Health Check in England: an evaluation of the first 4 years. *BMJ Open* 2016;6(1):e008840. doi: 10.1136/bmjopen-2015-008840
12. Herrett E, Gallagher AM, Bhaskaran K, et al. Data Resource Profile: Clinical Practice Research Datalink (CPRD). *Int J Epidemiol* 2015;44(3):827-36. doi: 10.1093/ije/dyv098
13. NHS Digital. NHS Health Check Programme: Interactive Dashboard 2019 [updated October 2019. Available from: https://digital.nhs.uk/data-and-information/data-tools-and-services/data-services/general-practice-data-hub/nhs-health-check-programme accessed February 2020.
14. NHS Digital. NHS Health Checks business rules NHS Digital 2018 [Available from: https://digital.nhs.uk/services/general-practice-gp-collections/service-information/nhs-health-checks-business-rules accessed February 2020.
15. NHS Digital. Data Extract Advisory Committee to the NHS Health Check data extract 2018 [Available from: https://www.healthcheck.nhs.uk/commissioners-and-providers/governance/data-extract-advisory-committe-deac/ accessed February 2020.
16. Benchimol EI, Smeeth L, Guttmann A, et al. The REporting of studies Conducted using Observational Routinely-collected health Data (RECORD) Statement. *PLOS Medicine* 2015;12(10):e1001885. doi: 10.1371/journal.pmed.1001885

For peer review only - http://bmjopen.bmj.com/site/about/guidelines.xhtml

17. NHS Digital. Privacy Notice: NHS Health Check for adults aged 40-74 years: NHS Digital 2019 [updated February 2019. Available from: https://digital.nhs.uk/services/general-practice-gp-collections/service-information/nhs-health-checks accessed February 2020.
18. NHS Digital. General Practice Extraction Service (GPES) 2019 [updated December 2019. Available from: https://digital.nhs.uk/services/general-practice-extraction-service accessed February 2020.
19. NHS Digital. Direction for the NHS health check for adults aged 40-74 years data extraction 2018 [updated October 2019. Available from: https://digital.nhs.uk/about-nhs-digital/corporate-information-and-documents/directions-and-data-provision-notices/public-health-england-directions/direction-for-the-nhs-health-check-for-adults-aged-40--74-years-data-extraction accessed February 2020.
20. Public Health England. Public Health Outcome Framework: NHS Health Check indicators: Public Health England; 2019 [updated December 2019. Available from: https://fingertips.phe.org.uk/profile/nhs-health-check-detailed accessed February 2020.
21. Usher-Smith. J MA, Harte. E, MacLure. C, Meads. C, Saunders. C, Griffin. S, Walter. F, Lawrence. K, Robertson. C, Mant. J,. NHS Health Check programme rapid evidence synthesis 2017 [Available from: www.healthcheck.nhs.uk/commissioners_and_providers/evidence/ accessed February 2020.
22. Attwood S, Morton K, Sutton S. Exploring equity in uptake of the NHS Health Check and a nested physical activity intervention trial. *J Public Health (Oxf)* 2016;38(3):560-68. doi: 10.1093/pubmed/fdv070
23. Dalton AR, Bottle A, Okoro C, et al. Uptake of the NHS Health Checks programme in a deprived, culturally diverse setting: cross-sectional study. *J Public Health (Oxf)* 2011;33(3):422-9. doi: 10.1093/pubmed/fdr034
24. Cochrane T, Gidlow CJ, Kumar J, et al. Cross-sectional review of the response and treatment uptake from the NHS Health Checks programme in Stoke on Trent. *J Public Health (Oxf)* 2013;35(1):92-8. doi: 10.1093/pubmed/fds088
25. Patel RS, Lagord C, Waterall J, et al. Online self-assessment of cardiovascular risk using the Joint British Societies (JBS3)-derived heart age tool: a descriptive study. *BMJ Open* 2016;6(9):e011511. doi: 10.1136/bmjopen-2016-011511
26. NHS Digital. Health Survey for England 2018 [updated December 2019. Available from: https://digital.nhs.uk/data-and-information/publications/statistical/health-survey-for-england/2018 accessed February 2020.
27. National Institute for Health and Care Excellence. Cardiovascular disease: risk assessment and reduction, including lipid modification 2014 [updated September 2016. Available from: https://www.nice.org.uk/guidance/cg181 accessed February 2020.
28. Public Health England. Health matters: preventing cardiovascular disease 2019 [Available from: https://www.gov.uk/government/publications/health-matters-preventing-cardiovascular-disease/health-matters-preventing-cardiovascular-disease#cvd-ambitions-and-secondary-prevention accessed May 2020.

For peer review only - http://bmjopen.bmj.com/site/about/guidelines.xhtml

Table 1- Attendance to an NHS Health Check by financial year among individuals aged 40 - 74 years in England between April 2012 and March 2017 (N=9,694,979)

**Financial Year**	**Individuals offered an NHS health check**	**Individuals attending an NHS health check**	**Uptake of offers rate %**
2012/2013	1,469,031	742,935	50.6
2013/2014	1,796,483	962,831	53.6
2014/2015	2,162,454	1,135,746	52.5
2015/2016	2,154,129	1,142,151	53.0
2016/2017	2,112,882	1,119,095	53.0
**Total**	**9,694,979**	**5,102,758**	**52.6**

For peer review only

For peer review only - http://bmjopen.bmj.com/site/about/guidelines.xhtml

Table 2: Socio-demographic characteristics of NHSHC invitees April 2012 - March 2017 compared with ONS estimated English population aged 40-74 at mid-2015

Socio-demographic characteristic	ONS mid-2015 England resident population (aged 40-74 years)	NHSHC Invitees (%)	Attendees n (%)	Non-attendees n (%)
**Sex**				
Male	11,200,690 (49.1)	4,724,015 (48.7)	2,311,604 (45.3)	2,412,411 (52.5)
Female	11,604,922 (50.9)	4,970,906 (51.3)	2,791,130 (54.7)	2,179,776 (47.5)
Unknown	-	58 (0.0)	24 (0.0)	34 (0.0)
**Age group (years)**				
40-44	3,636,454 (15.9)	2,208,213 (22.8)	984,908 (19.3)	1,223,305 (26.6)
45-49	3,889,360 (17.1)	1,986,966 (20.5)	966,356 (18.9)	1,020,610 (22.2)
50-54	3,811,000 (16.7)	1,833,267 (18.9)	958,263 (18.8)	875,004 (19.1)
55-59	3,278,322 (14.4)	1,414,091 (14.6)	783,740 (15.4)	630,351 (13.7)
60-64	2,904,721 (12.7)	1,105,914 (11.4)	669,503 (13.1)	436,411 (9.5)
65-69	3,017,135 (13.2)	910,089 (9.4)	585,653 (11.5)	324,436 (7.1)
70-74	2,268,620 (9.9)	236,439 (2.4)	154,335 (3.0)	82,104 (1.8)
**Ethnic Group**				
White	20,383,677 (89.4)	6,946,824 (71.7)	4,067,864 (79.7)	2,878,960 (62.7)
Indian	524,313 (2.3)	202,004 (2.1)	136,598 (2.7)	65,406 (1.4)
Pakistani	291,546 (1.3)	137,222 (1.4)	89,970 (1.8)	47,252 (1)
Bangladeshi	101,926 (0.4)	46,802 (0.5)	34,863 (0.7)	11,939 (0.3)
Black African	314,107 (1.4)	147,462 (1.5)	94,539 (1.9)	52,923 (1.2)
Black Caribbean	271,649 (1.2)	79,987 (0.8)	53,621 (1.1)	26,366 (0.6)
Chinese	121,129 (0.5)	44,730 (0.5)	27,360 (0.5)	17,370 (0.4)
Other Asian	302,667 (1.3)	125,853 (1.3)	79,354 (1.6)	46,499 (1)
Other Group	494,599 (2.2)	239,024 (2.5)	142,621 (2.8)	96,403 (2.1)
Not Stated		104,136 (1.1)	31,319 (0.6)	72,817 (1.6)
Missing		1,620,935 (16.7)	344,649 (6.8)	1,276,286 (27.8)
**Deprivation Index (IMD Decile)**				
Most deprived	1,914,356 (8.4)	853,547 (8.8)	420,547 (8.2)	433,000 (9.4)

For peer review only

For peer review only - http://bmjopen.bmj.com/site/about/guidelines.xhtml

2	1,999,183 (8.8)	896,809 (9.3)	472,647 (9.3)	424,162 (9.2)
3	2,083,743 (9.1)	904,131 (9.3)	477,140 (9.4)	426,991 (9.3)
4	2,202,902 (9.7)	921,244 (9.5)	477,516 (9.4)	443,728 (9.7)
5	2,304,663 (10.1)	974,023 (10)	509,715 (10.0)	464,308 (10.1)
6	2,402,719 (10.5)	991,135 (10.2)	517,381 (10.1)	473,754 (10.3)
7	2,443,073 (10.7)	1,044,505 (10.8)	547,909 (10.7)	496,596 (10.8)
8	2,458,761 (10.8)	1,034,751 (10.7)	547,016 (10.7)	487,735 (10.6)
9	2,491,679 (10.9)	1,045,098 (10.8)	565,872 (11.1)	479,226 (10.4)
Least deprived	2,504,533 (11.0)	1,022,539 (10.5)	563,798 (11.0)	458,741 (10.0)
Missing		7,197 (0.1)	3,217 (0.1)	3,980 (0.1)
**Patient characteristics**				
Deaf	n/a	321 (0.0)	171 (0.0)	150 (0.0)
Blind	n/a	13,405 (0.1)	7,224 (0.1)	6,181 (0.1)
Severe Mental Illness	n/a	111,878 (1.2)	59,351 (1.2)	52,527 (1.1)
Learning Disability	n/a	39,612 (0.4)	21,535 (0.4)	18,077 (0.4)
Dementia	n/a	7,521 (0.1)	3,060 (0.1)	4,461 (0.1)
Rheumatoid Arthritis	n/a	74,281 (0.8)	38,104 (0.7)	36,177 (0.8)
Total	22,805,612	9,694,979	5,102,758	4,592,221

ONS= Office for National Statistics, NHSHC = NHS Health Check, IMD = Index of multiple deprivation

For peer review only

For peer review only - http://bmjopen.bmj.com/site/about/guidelines.xhtml

Table 3 Number and proportion of attendees that were coded as received advice, information or a referral following their NHSHC among all attendees and attendees with CVD risk factors

**Intervention type**	**All Attendees n (%)**	**Attendees with the CVD risk factor above threshold for intervention n (%)**
Alcohol Consumption	792,761 (15.5)	46,611 (38.4)
Diet	1,189,986 (23.3)	766,521 (25.1)
Physical Activity	1,501,103 (29.4)	434,326 (39.3)
General Lifestyle/ Behaviours	814,611 (16.0)	211,571 (20.1)
Smoking Cessation	865,913 (17)	467,119 (57.3)
Weight Loss and Obesity	821,414 (16.1)	599,380 (19.6)
Diabetes Prevention Programme (DPP)	4,551 (0.1)	3,348 (0.9)
Total	*2,501,565 (49.0)*	*565,047 (53.7)*

Thresholds defined in Supplementary Table 8, DPP = diabetes prevention programme

For peer review only

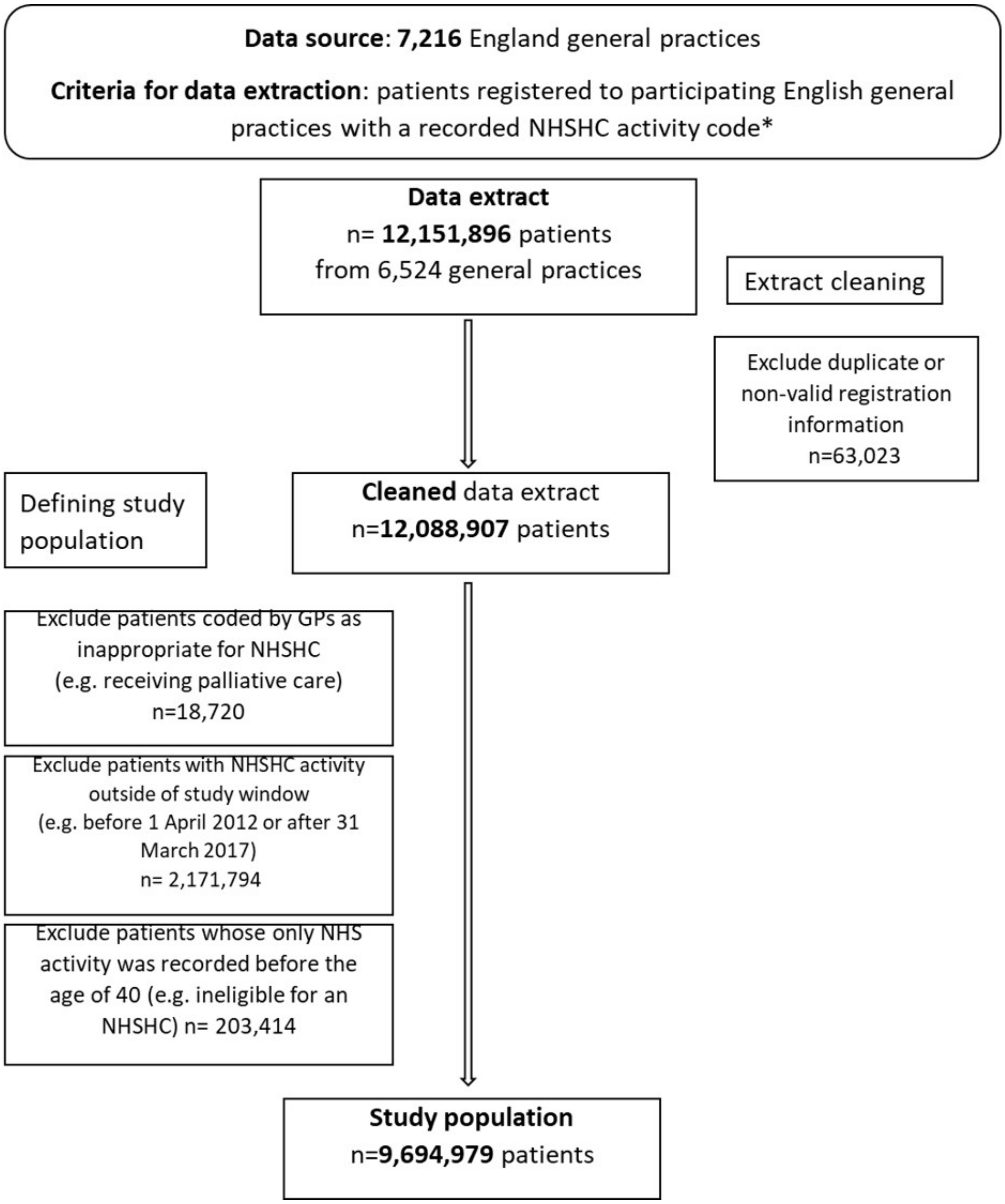

Figure 1: Study extract and study population flow chart

159x190mm (149 x 149 DPI)

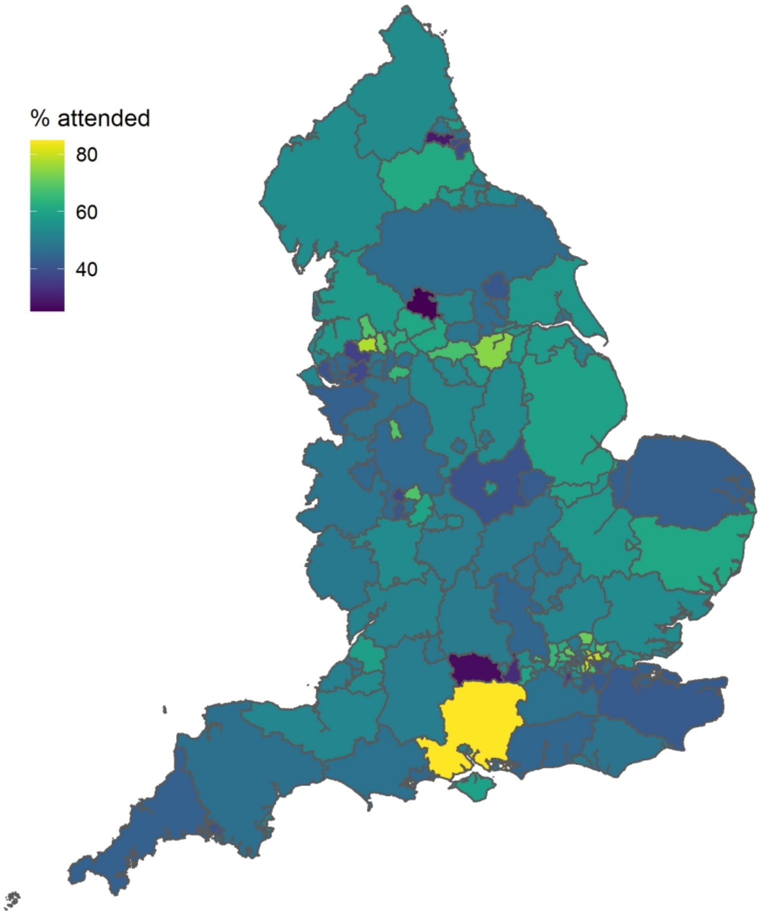

Figure 2: Variation in NHSHC uptake across (A) England

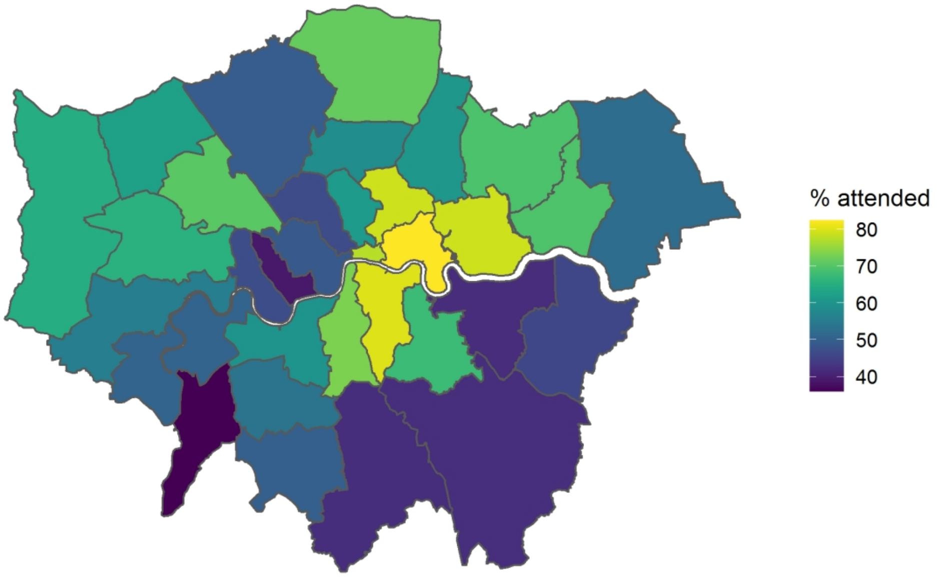

Figure 2: Variation in NHSHC uptake across (B) London

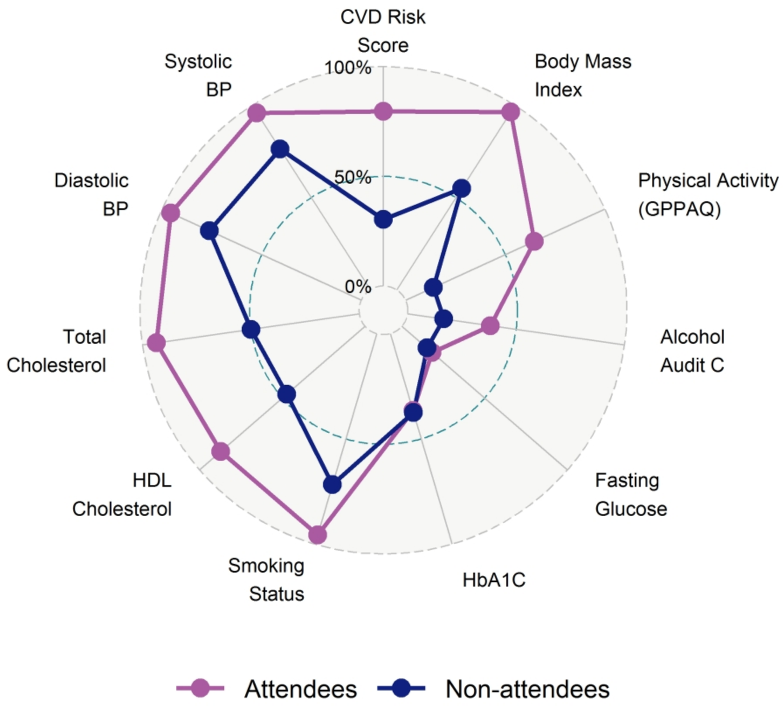

Figure 3: Completion of risk factor measurements for attendees and non-attendees (2012/13 - 2016/17)

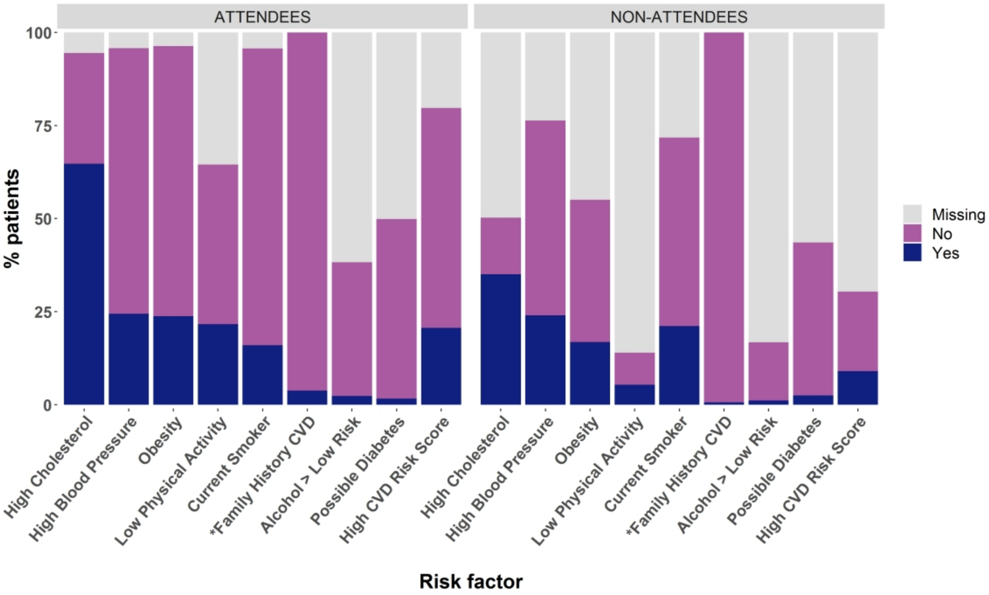

Figure 4: Proportion of attendees and non-attendees with common CVD risk factors

Supplementary Materials

An evaluation of the uptake and delivery of the NHS Health Check Programme in England, using primary care data from 9.5 million people: A cross-sectional study

Contents

Supplementary Methods 2
Supplementary Figures 4
Supplementary Figure 1 - Invitation type for first invitation record by year of invitation among attendees and non-attendees 4
Supplementary Tables 5
Supplementary Table 1: Read codes for NHS Health Check activity codes and prioritisation rules for definition of primary contact with programme 5
Supplementary Table 2: Data extraction rules 6
Supplementary Table 3: Plausible ranges for risk factor measurements 7
Supplementary Table 4: Order of priority for selecting metrics in time window around patient's index date 8
Supplementary Table 5: Derived Ethnic Group Categories 16
Supplementary Table 6: Categories for risk factors - Risk factors by binary cut points 17
Supplementary Table 7: Rules for conflicting risk factors measurements 18
Supplementary Table 8: Intervention risk thresholds for action 18
Supplementary Table 9: Data for attendance by UTLA 19
Supplementary Table 10: Number of invitations recorded for attendees and non-attendees 22
Supplementary Table 11: Invitations by financial year 23
Supplementary Table 12: Completeness of risk factor measurement 23
Supplementary Table 13: Statin prescription rates 24

For peer review only - http://bmjopen.bmj.com/site/about/guidelines.xhtml

Supplementary Methods

Data Management and Cleaning

The data extract was stored within a Structured Query Language (SQL) database and processed using queries within SQL Server Management Studio. Duplicate patient records were removed. Implausible values were re-coded as missing values. Plausible ranges for risk factors, Supplementary Table 3, were defined by DEAC.

Definitions and Study Variables

Individuals were categorised as either NHSHC attendees if they had a Read code for a completed check within the 5-year period, or a non-attendee if they did not. Further details are provided in Supplementary Table 1. Uptake of the programme was defined as the proportion of the total study population who attended.

An index date was generated from the date of an individual's primary NHSHC activity to identify age and the most relevant risk factor measurements for each patient. Risk factor and clinical measurements were selected for analysis if they occurred on the index date, otherwise we took the closest recording within pre-defined time windows set by the DEAC. A full list of variables, Read codes used to define variables, time windows and coding algorithms is available in Supplementary Table 4.

An individual's age in years was estimated based on year of birth and index date and presented in five-year intervals. We derived an ethnic group variable with the aim of generating fewer categories while still representing important ethnic groups for CVD (Supplementary Table 5). We also included Index of Multiple Deprivation (IMD) (2015) national deciles matched at Lower Super Output Area (LSOA) level based on the patient's postcode of residence at the time of data extraction.[1] ONS April 2019 upper tier local authority (UTLA) boundaries were used.[2] Gender was reported as coded in the extract (Male; Female). Learning difficulty, serious mental illness (SMI), blindness, deafness, rheumatoid arthritis and dementia (present/absent) are reported as binary variables.

We present the following risk factors as binary variables, using cut-points defined in consultation with DEAC, Supplementary Table 6; obesity (BMI>30kg/m^2), blood pressure (derived from systolic (>=140mmHg) or diastolic blood pressure (>=90mmHg), cholesterol (total cholesterol >5mmol/L or cholesterol ratio >4), blood glucose (fasting plasma glucose >=7mmol/L or HbA1C>=48mmol/mol), smoking (current), physical activity (general practice physical activity questionnaire = moderately

For peer review only - http://bmjopen.bmj.com/site/about/guidelines.xhtml

inactive or inactive), alcohol intake and behaviour (Audit C score >=8), CVD risk score (10 year risk >=10%) and family history of CVD before 60 years. Rules for conflicting measures for the same patient on the same day are available in Supplementary Table 7.

Among attendees, we considered invitations in the 365 days prior to the index date. Time to attendance was derived from the number of days between first recorded invitation and the index date. Invitation type for attendees was grouped into three categories: advanced invitation (invitation recorded prior to date of NHSHC), opportunistic invitation (invitation recorded same date as NHSHC) and missing invitation (invitation not recorded but NHSHC completed). Among non-attendees for whom the primary contact was an invitation, we considered invitations in the 365 days after the index date. The provider delivering the NHSHC (GP staff; third party) was reported as a binary variable.

Among attendees, we present data for delivery of advice, information or referral for diet, alcohol, physical activity, smoking, weight loss and general lifestyle, referrals for diabetes prevention and prescriptions for statins (present/absent) as binary variables. Statin prescribing data was made available by three out of four GP clinical IT system providers, and subsequently a Read code was attached to 60.4% of attendees in the dataset. We present data for any statin prescription on or after the date of NHSHC activity, as individuals with current statin prescriptions would not be eligible for an invitation to the NHSHC. We also present these data among attendees with a risk profile indicating that intervention was appropriate. We defined appropriate thresholds for action of intervention through consultation with the DEAC advisory board. These are available in Supplementary Table 8.

REFERENCES

1. Office for National Statistics. English indices of deprivation 2015 2015 [Available from: https://www.gov.uk/government/statistics/english-indices-of-deprivation-2015.
2. Office for National Statistics. Counties and Unitary Authorities (April 2019) Boundaries EW BFC 2019 [updated November 2019. Available from: https://geoportal.statistics.gov.uk/datasets/counties-and-unitary-authorities-april-2019-boundaries-ew-bfc accessed December 2019.

For peer review only - http://bmjopen.bmj.com/site/about/guidelines.xhtml

Supplementary Figures

Supplementary Figure 1 - Invitation type for first invitation record by year of invitation among attendees and non-attendees

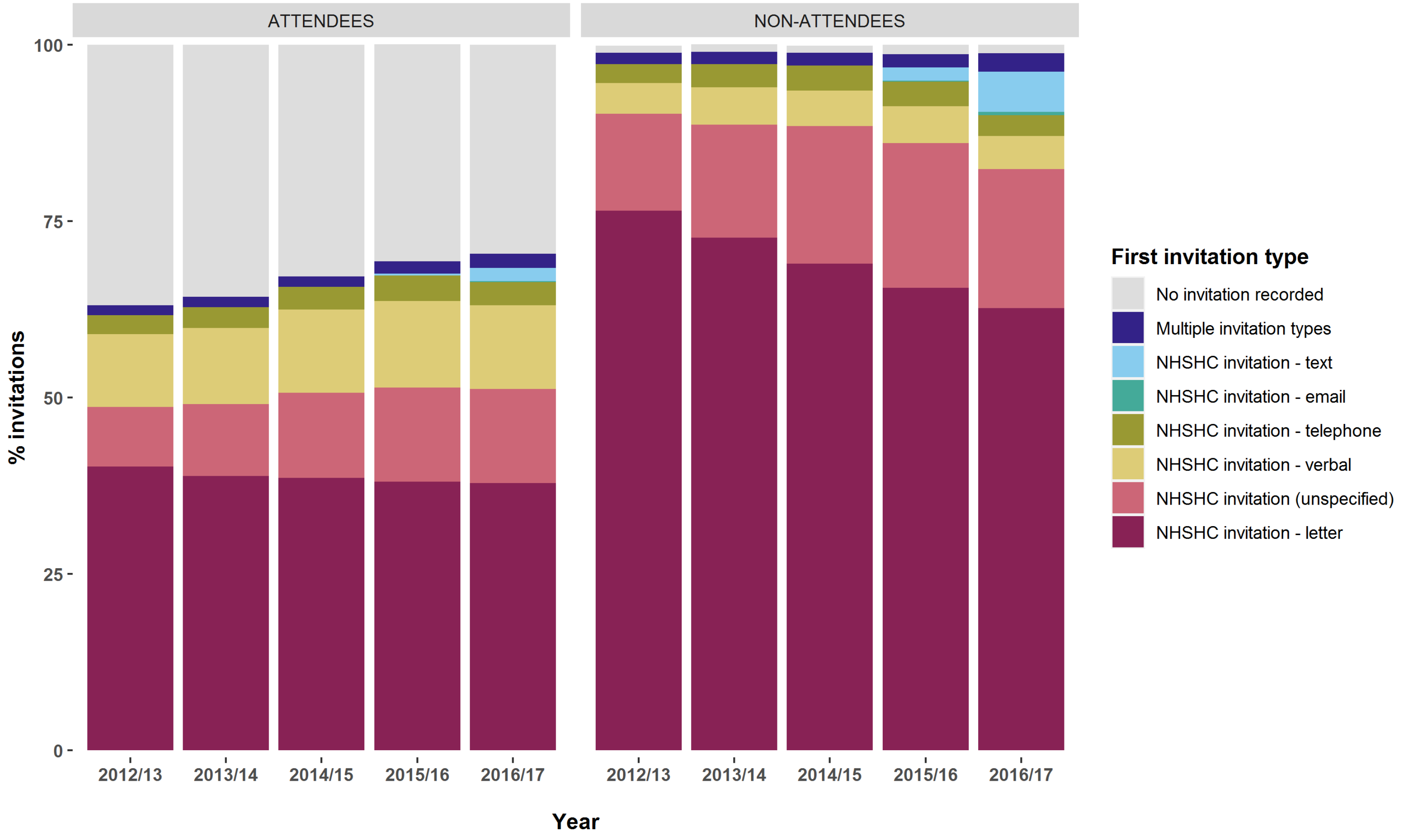

For peer review only - http://bmjopen.bmj.com/site/about/guidelines.xhtml

Supplementary Tables

Supplementary Table 1: Read codes for NHS Health Check activity codes and prioritisation rules for definition of primary contact with programme

Order	Clinical NHSHC activity code	Read V2 clinical codes (date introduced)	CTV3 clinical codes (date introduced)	Reported grouping	Criteria
1	Inappropriate	9NSH. (01/10/2013)	Xaaac (01/10/2013)	Excluded from study	Patient has a code recorded as being inappropriate for an NHS Health Check in the data extract
2	Completed	8BAg. (01/04/2010)<br><br>8BAg0 (01/10/2012)	XaRBQ (01/04/2010)<br><br>XaZPq (01/10/2012)	Attendee	Patient has a completed NHS Health Check code recorded in the 5-year period<br><br>**Index date**: date of patient's first completed check code
3	Declined	8IAx. (01/04/2011)	XaX8h (01/04/2011)	Non-attendee	Patient has a declined NHS Health Check code recorded in the 5-year period<br><br>**Index date:** date of patient's first declined code
4	Did not attend	9NiS. (01/04/2010)	XaRAA (01/04/2010)	Non-attendee	Patient has an NHS Health Check not attended code recorded in the 5-year period<br><br>**Index date:** date of patient's first non-attendance code
5	Commenced	8CV9. (01/04/2016)	Xaeab (01/04/2016)	Non-attendee	Patient has a commenced NHS Health Check code recorded in the 5-year period (and no completed/did not attend/declined code recorded in the following 8 weeks)<br><br>**Index date:** date of patient's first commenced code
6	Invitation	9mC.., 9mC0., 9mC1., 9mC2., 9mC3., 9mC4., (01/04/2010)<br><br>9mC5., 9mC6. (01/10/2015)	XaRBR, XaR9z, XaRBS, XaRBT, XaRBU, XaRBV (01/04/2010)<br><br>Xad0C, Xad0D, (01/10/2015)	Non-attendee	Patient has an invitation to attend an NHS Health Check code recorded in the 5-year period (and no follow up (non-invitation) code recorded within the following 6 months)<br><br>**Index date:** date of patient's first invitation code

For peer review only - http://bmjopen.bmj.com/site/about/guidelines.xhtml

Supplementary Table 2: Data extraction rules

Event of interest
(NHS Health check offer, completed, DNA, declined …)

GPES EXTRACT

Category data item	Details data item	before event	after event
Patients demographics & equity measures	Carer status	latest code before event	1 month
	Learning disabilities, serious mental illness, blindness, deafness, dementia	include only first recorded code (if not 'resolved')	
	Deprivation (calculated), age (calculated), gender, ehtnicity, first language	latest available code	
CVD risk factor	Smoking status, height, weight, BMI, waist circum., alcohol use, physical activity, CVD risk score	all codes 2 years before event	all codes after event
Tests results	Blood pressure, pulse, cholesterol & HDL, glucose/ HbA1c, eGFR, ACR	all codes 2 years before event	all codes after event
CVD diagnosis (**)	Family history CVD, CHD, CKD, diabetes type 2, TIA, stroke, AF, Heart failure, hypertension, familial hypercholesterolemia, hypercholesterolemia, hyperlipidaemia, PAD. (+ non-diabetic hyperglycaemia, impaired glucose regulation, impaired fasting glycaemia, impaired glucose tolerance) (+ rheumatoid arthritis)	all codes (if not resolved)	
Referrals	Referral to NHS DPP (start 2016)	all codes	
	Advice, signposting, referral to lifestyle services, referral for other CVD assessment (diabetes, hypertension, …)	latest code before event	all codes within 1 year of event
Prescribed CVD medication	Lipid regulation, anti-hypertensive, anti-coagulant, diabetes treatment, AF treatment	all codes 2 years before event	all codes within 1 year of event

Supplementary Table 3: Plausible ranges for risk factor measurements

Risk factor	Plausible measurement range (inclusive unless stated)
Alcohol risk score (AUDIT; AUDITC; FAST)	0 – 40
Blood pressure - systolic	70 – 300 mmHg
Blood pressure - diastolic	20 – 150 mmHg
BMI	12 – 90 kg/m^2
Cholesterol – total	1 – 40 (exclusive)
Cholesterol – HDL	0.5 – 5
Cholesterol – ratio	0.2 – 80
Fasting Plasma Glucose (FPG)	0 (exclusive) – 100
HbA1c	20 – 195 mmol/mol
Height	100 – 230 cm
CVD risk score	0 – 100
Weight	20 – 250 kg

For peer review only

For peer review only - http://bmjopen.bmj.com/site/about/guidelines.xhtml

Supplementary Table 4: Order of priority for selecting metrics in time window around patient's index date

Metric	First priority	Second priority	Third priority	Derivation / other prioritisation rules	Clinical codes (Read V2)	Clinical codes (CTV3)
**Patient characteristics**						
Ethnic group	Ethnic group recorded in patient's GPES profile at time of data extraction (31/3/2018)	Most recent ethnic group recorded via a clinical code (looking over whole data extract)	n/a	n/a	9S...% , 9T...% , 9t...% , 9i...%	XaBEN%
Blindness	On index date	Anytime before index date (most proximal to index date used)	n/a	n/a	6689. , 6688. , 668D. , 668C.	6689.% , XaW0l , XaCGX% , XaLMz
Deafness	On index date	Anytime before index date (most proximal to index date used)	n/a	n/a	F599. , F591B , F591E , F59A. , F5919	XaRE4 , XaZuB , XaZuE , XaaLf , XaRE5 , Xa0PN
Dementia	On index date	Anytime before index date (most proximal to index date used)	n/a	n/a	Eu02.% , E00..% , Eu01.% , E02y1 , E012.% , Eu00.% , E041. , Eu041 , F110.- F112. , F116. , F118. , F21y2 , A410. , A411.%	X002w% (excluding X003E , X003F , X001T) , Eu02.% , XE1Xt , E00z. , E02y1
Learning Disability	On index date	Anytime before index date (most proximal to index date used)	n/a	n/a	E3...% , Eu7..% , Eu814 , Eu815 , Eu816 , Eu817 , Eu81z , 918e. , Eu818	E3...% , XaQZ4 , XaQZ3 , XaKYb , XaREt , XaREu , Eu81z , XaaiS , Xabk1
Severe Mental Illness	On index date	Anytime before index date (most proximal to index date used)	n/a	n/a	E10..% , E110.% , E111.% , E1124 , E1134 , E114.- E117z , E11y.% (excluding E11y2) , E11z. , E11z0 , E11zz , E12..% , E13..% (excluding E135.) , E2122 , Eu2..% , Eu30.%	X00S6% (excluding Xa9B0% , E14..%) , X00SL , X00SM% , X00SJ% , XSGon , E11z. , E11z0 , E11zz , XE1ZZ , XE1Ze , XaX54 , XaX53 , E130. , E1124 , E1134

For peer review only - http://bmjopen.bmj.com/site/about/guidelines.xhtml

					, Eu31.% , Eu323 , Eu328 , Eu333 , Eu32A , Eu329	
**CVD risk factors**						
Family history of CVD	On index date	Anytime before index date (most proximal to index date used)	Anytime after index date (most proximal to index date used)	n/a	12C.. , 12C2. , 12C3. , 12C4. , 12C5. , 12CA. , 12CB. , 12CC. , 12CD. , 12CE. , 12CF. , 12CG. , 12CH. , 12CI. , 12CL. , 12CM. , 12CN. , 12CP. , 12CV. , 12CW. , 12CZ.	XaP9K , XaP9M , ZV174 , XE24Z , XaLQq , Xa6aj% , XM1Jg , XM1Jw% , XaP9K , XaP9M
Rheumatoid arthritis	On index date	Anytime before index date (most proximal to index date used)	**Attendees:** n/a<br><br>**Non-attendees:** Anytime after index date (most proximal to index date used)	n/a	N040.% , N041. , N042.% (excluding N0420) , N047. , N04X. , N04y0 , N04y2 , Nyu11 , Nyu12 , Nyu1G , Nyu10 , G5yA. , G5y8.	N040.% , XE1DU , X705I , G5y8.
Alcohol AUDIT/AUDIT-C/FAST	On index date	Most proximal score to index date for each of AUDIT, AUDIT-C and FAST used.<br><br>**Attendees:** Up to 365 days before index date<br><br>**Non-attendees:** Anytime before index date	Most proximal score to index date for each of AUDIT, AUDIT-C and FAST used.<br><br>**Attendees:** Up to 90 days after index date<br><br>**Non-attendees:** Anytime after index date	No AUDIT-C/FAST/AUDIT score available: risk factor is **missing**<br><br>AUDIT-C or FAST assessment is positive, but no AUDIT score available: risk factor is **missing**<br><br>AUDIT-C (and/or) FAST assessment is negative: risk factor is **low risk**<br><br>AUDIT score available and greater than or equal to 8: risk factor is **high risk**	38D4. (AUDIT-C), 388u. (FAST), 38D3. (AUDIT)	XaORP (AUDIT-C), XaNO9 (FAST), XM0aD (AUDIT)

For peer review only - http://bmjopen.bmj.com/site/about/guidelines.xhtml

Blood pressure	On index date	Systolic and diastolic BP recordings recorded most proximal to index date used.<br><br>**Attendees:** Up to 365 days before index date<br><br>**Non-attendees:** Anytime before index date	Systolic and diastolic BP recordings recorded most proximal to index date used.<br><br>**Attendees:** Up to 90 days after index date<br><br>**Non-attendees:** Anytime after index date	On examination (O/E) readings considered only.<br><br>Systolic BP or Diastolic BP is unavailable: risk factor is **missing**	246..% (excluding 2460. , 2468. , 246H. , 246I. , 246K. , 246L. , 246M. , 246h. , 246i. , 246j. , 246k. , 246n.% , 246o.%)	X773t% (excluding XaI9f , XaI9g , XaZvo , XaZxj , X779b , X779R , X779T , X779W , XaYai , XaYg8 , XaYg9 , Xabhx , Xac5K , Xac5L , Xaedn%) , 246..% (excluding 2460. , 2468. , XaCFN , XaCFO)
Blood glucose	On index date	HbA1c and Fasting Plasma Glucose recorded most proximal to index date considered.<br><br>**Attendees:** Up to 365 days before index date<br><br>**Non-attendees:** Anytime before index date	HbA1c and Fasting Plasma Glucose recorded most proximal to index date considered.<br><br>**Attendees:** Up to 90 days after index date<br><br>**Non-attendees:** Anytime after index date		HbA1c:<br>42W5. , 42W50 , 42W51<br><br>Fasting Plasma Glucose:<br>44g1.	HbA1c:<br>XaPbt , Xaezd , Xaeze<br><br>Fasting Plasma Glucose:<br>44g1.
Body mass index	On index date	Most proximal to index date used.<br><br>**Attendees:** Up to 365 days before index date<br><br>**Non-attendees:** Anytime before index date	Most proximal to index date used.<br><br>**Attendees:** Up to 90 days after index date<br><br>**Non-attendees:** Anytime after index date	If BMI is unavailable but height and weight are, BMI is calculated (BMI = kg/m^2)<br><br>Height and weight are not used if BMI is available	BMI:<br>22K..% (excluding 22K9.% , 22KA.)<br><br>Weight:<br>22A..% (excluding 22A7.-22A9.) , 9NSa. , 8IAH.<br><br>Height:<br>229..% (excluding 2296.) , 9NSZ. , 8IHM.	BMI:<br>22K..% (excluding XaVwA% , X76CN , XaZMj) , Xa7wG%<br><br>Weight:<br>22A..% , 22AA. , X76C3 , XaesG , XaQ7T<br><br>Height:

For peer review only - http://bmjopen.bmj.com/site/about/guidelines.xhtml

						229..% (excluding 2296.) , XaesF , Xaef4
Cholesterol (ratio)	On index date	Most proximal to index date used.<br><br>**Attendees:** Up to 365 days before index date<br><br>**Non-attendees:** Anytime before index date	Most proximal to index date used.<br><br>**Attendees:** Up to 90 days after index date<br><br>**Non-attendees:** Anytime after index date	If cholesterol ratio is unavailable but total and HDL cholesterol are, the cholesterol ratio is calculated (ratio = total/HDL)<br><br>Total and HDL cholesterol are not used if cholesterol ratio is available	Cholesterol:<br>44O5. , 44PH. , 44P5. , 44PF. , 44PJ. , 44P.. , 44OE. , 44P1. , 44P2. , 44P3. , 44P4. , 44PK. , 44PZ. , 44l2. , 44lF. , 44lG. , 662a.<br><br>HDL cholesterol:<br>44P5. , 44PB. , 44PC. , 44d3. , 44d2.	Cholesterol:<br>XaFs9 , XSK14 , 44P5. , 44PF , 44PJ. , XalRd , XE2eD% , 44P1. , 44P2. , 44P3. , 44P4. , 44PH. , XaERR , XaEUq , XaEUr , X772L<br><br>HDL cholesterol:<br>X772M , 44P5. , 44PB. , 44PC. , XaEVr , 44d3. , 44d2.
Physical activity (GPPAQ)	On index date	Most proximal to index date used.<br><br>**Attendees:** Up to 365 days before index date<br><br>**Non-attendees:** Anytime before index date	Most proximal to index date used.<br><br>**Attendees:** Up to 90 days after index date<br><br>**Non-attendees:** Anytime after index date	n/a	138b. , 138a. , 138Y. , 138X. , 38Dh.	XaPPE , XaPPD , XaPPB , XaPP8 , XaXX5
CVD risk score	On index date	QRISK/QRISK2 and Framingham risk score recorded most proximal to index date used.<br><br>**Attendees:** Up to 365 days before index date	QRISK/QRISK2 and Framingham risk score recorded most proximal to index date used.<br><br>**Attendees:** Up to 90 days after index date	QRISK or QRISK2 score recorded most proximal to index date is used if available.<br><br>If QRISK and QRISK2 unavailable, Framingham score is used.	QRISK/QRISK2:<br>8IEL., 8IEV., 38DF., 38DP.<br><br>Framingham:<br>38DR.	QRISK/QRISK2:<br>XaYzy, XaZdA, XaPBq, XaQVY<br><br>Framingham:<br>XaQaG

For peer review only - http://bmjopen.bmj.com/site/about/guidelines.xhtml

		**Non-attendees:** Anytime before index date	**Non-attendees:** Anytime after index date			
Smoking status	On index date	Most proximal to index date used.<br><br>**Attendees:** Up to 365 days before index date<br><br>**Non-attendees:** Anytime before index date	Most proximal to index date used.<br><br>**Attendees:** Up to 90 days after index date<br><br>**Non-attendees:** Anytime after index date	Lookup used to map smoking status to binary categories: Non-smoker; Current smoker	Non-smoker: 1371, 137A., 137l., 137N., 137O., 137S.,<br><br>Current smoker: 137.., 137C., 137e., 137h., 137m., 137P., 137Q., 137R., 137V., 137X., 137Y.,	Non-smoker: 1371, 1377, 1378, 1379, 137B., 137F., 137K., 137T., Ub0p1, Ub1na, Xa1bv, XaQ8V, XE0oj, XE0ok, XE0ol, XE0om, XE0on, XE0op, XE0oh<br><br>Current smoker: 1372, 1373, 1374, 1375, 1376, 137D., 137G., 137J., 137Z., Ub1tI, Ub1tJ, Ub1tK, Ub1tR, Ub1tS, Ub1tU, Ub1tW, XaIlu, XaIkW, XaIkX, XaIkY, XaItg, XaJX2, XaLQh, XaWNE, XaZIE, XE0oq, XE0or
**Interventions – attendees only**						
Advice, informatio n, referral – ALCOHOL	On index date	Up to 365 days after index date	n/a	n/a	Advice, information and any brief intervention given on alcohol usage: 67H0. , 67A5. , 8CAM. , 8CAM0 , 8CAv. , 8CE1. , 9k1A. , 8IAF. , 8IAt. , 9k11. , 9k14. , ZV6D6 , 6792. , 8CdK.<br><br>Referral regarding alcohol usage:	Advice, information and any brief intervention given on alcohol usage: XaJlr , Xa1dA , 67A5. , XaFvp , XaXan , XaPmB , 8CE1. , XaPPv , XaPty , XaX4S , XaKAC , XaKAo , ZV6D6 , 6792. , Xac6H<br><br>Referral regarding alcohol usage:

For peer review only - http://bmjopen.bmj.com/site/about/guidelines.xhtml

					8HkG. , 8H7p. , 8HHe.	XaYWV , XaIPn , XaKUg , XaPna , XaORR
Advice, informatio n, referral – DIET	On index date	Up to 365 days after index date	n/a	n/a	Advice, signposting or information on diet: 67H7. , 8CA4. , 8CA40 , 6799.<br><br>Referral regarding diet: 8H76. , 8H760 , 8HHE.	Advice, signposting or information on diet: XaQaU , 8CA4. , XaXTD , Xa2jQ , XE0i1 , Xa2hD , 6799.<br><br>Referral regarding diet: XaBSz , XaAhZ , XaAha , XaJSp , XaAdX , XaAdY , XaAdZ
Advice, informatio n, referral – LIFESTYLE	On index date	Up to 365 days after index date	n/a	n/a	67H..% , 8Hlu.	XaEFY% , Xaam2
Advice, informatio n, referral – PHYSICAL ACTIVITY	On index date	Up to 365 days after index date	n/a	n/a	Advice, signposting or information on physical activity: 67H2. , 8CA5. , 9Oq3. , 6798. , 8CA52 , 8Cd4. , 8IAv. , 8HBN.<br><br>Referral regarding physical activity: 8H7q. , 8H7q0 , 8HHc. , 8HkX. , 8BAH.	Advice, signposting or information on physical activity: XaJIt , Xa1dN , 8CA5. , XM18T , XaPjx , 6798. , XabFV , XaREx , XaX5H , XaREy<br><br>Referral regarding physical activity: XaIPu , XaR5C , XaKRq , XaREh , XaCmH
Advice, informatio n, referral	On index date	Up to 365 days after index date	n/a	n/a	Support and refer Stop Smoking Service/Advisor:	Support and refer Stop Smoking Service/Advisor:

For peer review only - http://bmjopen.bmj.com/site/about/guidelines.xhtml

– SMOKING					8CAL. , 8HTK. , 8HkQ. , 8H7i. , 8IAj. , 8IEK. , 9N2k. , 13p50 , 9Ndf. , 9Ndg. , 8T08. , 8IEo.<br><br>Advice, signposting or information on smoking: 67H1. , 8CAL. , 67A3. , 8CAg. , 6791. , 8IAj. , 8CdB.	Ua1Nz , XaFw9 , XaQT5 , XaItC , XaIye , XaW0h , XaX5W , XaX5X , XaRFh , XaREz , XaaDy , XaaDx<br><br>Advice, signposting or information on smoking: XaJIs , Ua1Nz , 67A3. , Ua1O0 , XaLD4 , 6791. , XaRFh , XaXnG
Advice, information, referral – WEIGHT	On index date	Up to 365 days after index date	n/a	n/a	Advice, signposting or information on weight management: 67I9. , 8CA40 , 8Cd7. , 66CQ. , 679P. , 8CdC. , 8IAu.<br><br>Referral regarding weight management: 8HHH. , 8HHH1 , 8HHH0 , 8H4n.	Advice, signposting or information on weight management: XaADJ , Xa1dF , XaX5F , XaX5k , XaKHd , XaXnI , XaX5G<br><br>Referral regarding weight management: XaJSu , XaZKe , XaXZ9 , XaZKi
Diabetes Prevention Programme referral	On index date	Up to 365 days after index date	n/a	n/a	679m4, 679m0, 679m1, 679m2	XaeDH, XaeCw, XaeCz, XaeD0
Statin prescriptions	On index date	Up to 365 days after index date	n/a	n/a	bxi..% , bxg..% , bxe..% , bxk..% , bxd..%<br><br>DM+D codes (EMIS): 134489001, 319996000, 319997009, 320000009,	bxi..% , x01R2% , x01R3% , bxk..% , bxd..%

For peer review only - http://bmjopen.bmj.com/site/about/guidelines.xhtml

					320006003,<br>320012008,<br>320013003,<br>320014009,<br>320029006,<br>320030001,<br>320031002,<br>408036003,<br>408037007,<br>409108001,<br>4896711000001108	

For peer review only

For peer review only - http://bmjopen.bmj.com/site/about/guidelines.xhtml

Supplementary Table 5: Derived Ethnic Group Categories

**Ethnic group**	**Subgroups (with ONS codes)**
White	A = White British
	B = Irish
	C = Any other White background
	T = White: Gypsy or Irish Traveller
Indian	H = Indian
Pakistani	J = Pakistani
Bangladeshi	K = Bangladeshi
Black African	N = African
Black Caribbean	M = Caribbean
Chinese	R = Chinese
Other Asian	L = Any other Asian background
Other Ethnic Group	D = White and Black Caribbean
	E = White and Black African
	F = White and Asian
	G = Any other mixed background
	P = Any other Black background
	S = Any other ethnic group
	W = Other ethnic group: Arab
Unknown	X = Unknown/No information
	Z = Not stated

For peer review only - http://bmjopen.bmj.com/site/about/guidelines.xhtml

Supplementary Table 6: Categories for risk factors - Risk factors by binary cut points

Risk factors by binary risk cut-offs

Risk factor	High risk threshold/ cutpoint	Risk category	Attendees n (%)	Non-attendees n(%)	Total
Alcohol > Low Risk	Full AUDIT score 8 or more	Missing	3,150,667 (61.7)	3,823,634 (83.3)	6,974,301
		Low risk	1,830,799 (35.9)	714,947 (15.6)	2,545,746
		High risk	121,292 (2.4)	53,640 (1.2)	174,932
Possible Diabetes	HbA1C ≥ 48 or FPG ≥ 7	Missing	2,558,719 (50.1)	2,590,405 (56.4)	5,149,124
		Low risk	2,460,489 (48.2)	1,885,332 (41.1)	4,345,821
		High risk	83,550 (1.6)	116,484 (2.5)	200,034
High Blood Pressure	Systolic BP ≥ 140 or Diastolic BP ≥ 90	Missing	217,714 (4.3)	1,086,797 (23.7)	1,304,511
		Low risk	3,636,511 (71.3)	2,404,097 (52.4)	6,040,608
		High risk	1,248,533 (24.5)	1,101,327 (24)	2,349,860
Obesity	BMI ≥ 30	Missing	187,402 (3.7)	2,064,936 (45)	2,252,338
		Low risk	3,700,522 (72.5)	1,755,019 (38.2)	5,455,541
		High risk	1,214,834 (23.8)	772,266 (16.8)	1,987,100
High Cholesterol	Total cholesterol >5mmol/L or Ratio > 4	Missing	282,100 (5.5)	2,286,595 (49.8)	2,568,695
		Low risk	1,519,485 (29.8)	696,458 (15.2)	2,215,943
		High risk	3,301,173 (64.7)	1,609,168 (35.0)	4,910,341
CVD risk score	10 or more	Missing	1,036,820 (20.3)	3,197,683 (69.6)	4,234,503
		Low risk	3,014,556 (59.1)	979,685 (21.3)	3,994,241
		High risk	1,051,382 (20.6)	414,853 (9)	1,466,235
Family history of CVD	Clinical code present for a CVD event before 60 years old in a first degree relative	No	4,910,543 (96.2)	4,561,766 (99.3)	9,472,309
		Yes	192,215 (3.8)	30,455 (0.7)	222,670
Physical Activity	GPPAQ “moderately inactive” or “inactive”	Missing	1,812,161 (35.5)	3,952,015 (86.1)	5,764,176
		Low risk	2,184,515 (42.8)	392,263 (8.5)	2,576,778
		High risk	1,106,082 (21.7)	247,943 (5.4)	1,354,025
Smoking	Current smoker	Missing	221,351 (4.3)	1,296,474 (28.2)	1,517,825
		Low risk	4,066,412 (79.7)	2,325,196 (50.6)	6,391,608
		High risk	814,995 (16)	970,551 (21.1)	1,785,546

For peer review only - http://bmjopen.bmj.com/site/about/guidelines.xhtml

Supplementary Table 7: Rules for conflicting risk factors measurements

Rules for processing conflicting risk factor measurements for the same patient on the same day

Risk factor	Rule applied
Smoking status; Physical activity status (from GPPAQ)	Records deleted if descriptive statuses are conflicting (e.g. “smoker” and “non-smoker” recorded on the same day)
Blood pressure	Record with lowest systolic measurement taken
BMI; height; weight; QRISK/QRISK2 score; Framingham score; total cholesterol; HDL cholesterol; Cholesterol ratio; HbA1c; FPG	Measurements recoded as missing (unclear which is correct)

Supplementary Table 8: Intervention risk thresholds for action

Intervention type	Advice or Information given	High risk threshold for action
**Advice, information or referral**	Alcohol usage	Alcohol: FULL AUDIT 8 or more
	Diet	Overweight (BMI ≥ 25)
	Physical activity	GPPAQ “moderately inactive” or “inactive”
	Lifestyle/Counselling	CVD risk score 10 or more
	Smoking cessation	Current smoker
	Weight management	Overweight (BMI ≥ 25)
**Diabetes referral**	Diabetes Prevention Programme (DPP) referral	Blood glucose: RAISED risk HbA1C ≥ 42 and < 48 or FPG ≥ 5.5 and < 7
**Statin prescription**	Statins prescribed	CVD risk score 10 or more

For peer review only - http://bmjopen.bmj.com/site/about/guidelines.xhtml

Supplementary Table 9: Data for attendance by UTLA

Number of NHS Health Check invitees and attendees with attendance rate by Upper Tier Local Authority of patient's residence

UTLA Code	UTLA	Invitees	Attendees	Attendance rate	Lower 95% CI	Upper 95% CI
E10000014	Hampshire	179,937	152,318	84.7	84.5	84.8
E09000030	Tower Hamlets	42,098	34,660	82.3	82.0	82.7
E09000028	Southwark	41,938	33,536	80.0	79.6	80.3
E09000025	Newham	51,556	40,706	79.0	78.6	79.3
E09000012	Hackney	37,636	29,713	78.9	78.5	79.4
E08000001	Bolton	64,013	49,792	77.8	77.5	78.1
E09000001	City of London	1,176	910	77.4	74.9	79.7
E08000017	Doncaster	19,869	14,736	74.2	73.6	74.8
E06000053	Isles of Scilly	482	353	73.2	69.1	77.0
E09000022	Lambeth	35,757	26,172	73.2	72.7	73.7
E09000010	Enfield	38,337	27,370	71.4	70.9	71.8
E09000005	Brent	68,977	48,573	70.4	70.1	70.8
E08000002	Bury	31,309	21,979	70.2	69.7	70.7
E09000002	Barking and Dagenham	36,578	25,402	69.4	69.0	69.9
E09000026	Redbridge	51,865	35,942	69.3	68.9	69.7
E06000021	Stoke-on-Trent	55,178	37,866	68.6	68.2	69.0
E06000008	Blackburn with Darwen	17,852	12,192	68.3	67.6	69.0
E08000030	Walsall	49,943	33,947	68.0	67.6	68.4
E09000023	Lewisham	26,396	17,838	67.6	67.0	68.1
E08000016	Barnsley	51,420	34,550	67.2	66.8	67.6
E09000009	Ealing	61,109	40,012	65.5	65.1	65.9
E06000039	Slough	16,191	10,600	65.5	64.7	66.2
E09000017	Hillingdon	45,539	29,447	64.7	64.2	65.1
E08000007	Stockport	44,540	28,763	64.6	64.1	65.0
E08000005	Rochdale	36,853	22,967	62.3	61.8	62.8
E09000015	Harrow	29,691	18,476	62.2	61.7	62.8
E06000047	County Durham	120,544	73,877	61.3	61.0	61.6
E09000019	Islington	38,209	23,415	61.3	60.8	61.8
E08000033	Calderdale	41,631	25,247	60.6	60.2	61.1
E09000031	Waltham Forest	50,680	30,720	60.6	60.2	61.0
E08000034	Kirklees	97,779	59,189	60.5	60.2	60.8
E10000029	Suffolk	147,142	89,051	60.5	60.3	60.8
E09000032	Wandsworth	57,469	34,442	59.9	59.5	60.3
E08000025	Birmingham	178,771	106,909	59.8	59.6	60.0
E06000036	Bracknell Forest	19,697	11,778	59.8	59.1	60.5
E10000019	Lincolnshire	200,192	119,037	59.5	59.2	59.7
E06000046	Isle of Wight	24,068	14,251	59.2	58.6	59.8
E08000004	Oldham	34,227	20,184	59.0	58.4	59.5
E06000031	Peterborough	44,281	26,027	58.8	58.3	59.2
E06000025	South Gloucestershire	59,350	34,683	58.4	58.0	58.8

For peer review only - http://bmjopen.bmj.com/site/about/guidelines.xhtml

E09000014	Haringey	29,867	17,448	58.4	57.9	59.0
E08000022	North Tyneside	40,154	23,434	58.4	57.9	58.8
E06000013	North Lincolnshire	24,121	13,870	57.5	56.9	58.1
E10000017	Lancashire	218,451	125,262	57.3	57.1	57.5
E06000005	Darlington	27,163	15,546	57.2	56.6	57.8
E06000011	East Riding of Yorkshire	12,161	6,894	56.7	55.8	57.6
E10000003	Cambridgeshire	116,035	65,679	56.6	56.3	56.9
E08000018	Rotherham	7,953	4,476	56.3	55.2	57.4
E06000016	Leicester	40,169	22,547	56.1	55.6	56.6
E06000034	Thurrock	32,083	17,982	56.0	55.5	56.6
E09000018	Hounslow	44,165	24,579	55.7	55.2	56.1
E10000006	Cumbria	120,237	65,183	54.2	53.9	54.5
E06000040	Windsor and Maidenhead	21,114	11,418	54.1	53.4	54.7
E06000057	Northumberland	75,940	40,859	53.8	53.4	54.2
E10000034	Worcestershire	141,667	76,000	53.6	53.4	53.9
E10000012	Essex	331,942	178,015	53.6	53.5	53.8
E10000024	Nottinghamshire	198,187	106,221	53.6	53.4	53.8
E09000024	Merton	43,144	23,114	53.6	53.1	54.0
E06000022	Bath and North East Somerset	44,466	23,810	53.5	53.1	54.0
E06000004	Stockton-on-Tees	35,341	18,857	53.4	52.8	53.9
E08000014	Sefton	48,044	25,630	53.3	52.9	53.8
E08000026	Coventry	64,356	34,306	53.3	52.9	53.7
E06000002	Middlesbrough	23,037	12,243	53.1	52.5	53.8
E08000019	Sheffield	80,302	42,628	53.1	52.7	53.4
E10000007	Derbyshire	197,165	104,520	53.0	52.8	53.2
E08000035	Leeds	174,645	92,288	52.8	52.6	53.1
E06000003	Redcar and Cleveland	25,185	13,304	52.8	52.2	53.4
E08000015	Wirral	80,558	42,456	52.7	52.4	53.0
E10000027	Somerset	75,851	39,814	52.5	52.1	52.8
E10000015	Hertfordshire	200,153	104,948	52.4	52.2	52.7
E09000016	Havering	42,627	22,305	52.3	51.9	52.8
E06000012	North East Lincolnshire	38,004	19,816	52.1	51.6	52.6
E08000029	Solihull	32,476	16,930	52.1	51.6	52.7
E10000013	Gloucestershire	137,245	71,077	51.8	51.5	52.1
E06000045	Southampton	33,058	17,102	51.7	51.2	52.3
E06000038	Reading	8,400	4,338	51.6	50.6	52.7
E06000027	Torbay	31,524	16,268	51.6	51.1	52.2
E06000024	North Somerset	40,162	20,498	51.0	50.5	51.5
E06000001	Hartlepool	12,989	6,616	50.9	50.1	51.8
E09000027	Richmond upon Thames	33,597	17,021	50.7	50.1	51.2
E06000033	Southend-on-Sea	48,006	24,182	50.4	49.9	50.8
E06000054	Wiltshire	114,656	57,526	50.2	49.9	50.5
E10000031	Warwickshire	102,623	51,428	50.1	49.8	50.4
E09000029	Sutton	24,049	11,959	49.7	49.1	50.4

For peer review only - http://bmjopen.bmj.com/site/about/guidelines.xhtml

E10000025	Oxfordshire	175,246	87,139	49.7	49.5	50.0
E06000056	Central Bedfordshire	73,732	36,607	49.6	49.3	50.0
E08000021	Newcastle upon Tyne	32,888	16,287	49.5	49.0	50.1
E10000021	Northamptonshire	155,686	76,979	49.4	49.2	49.7
E09000003	Barnet	52,312	25,849	49.4	49.0	49.8
E08000006	Salford	34,274	16,934	49.4	48.9	49.9
E06000019	Herefordshire, County of	37,499	18,421	49.1	48.6	49.6
E06000018	Nottingham	52,693	25,880	49.1	48.7	49.5
E06000043	Brighton and Hove	33,275	16,336	49.1	48.6	49.6
E06000030	Swindon	18,496	9,078	49.1	48.4	49.8
E06000023	Bristol, City of	58,017	28,467	49.1	48.7	49.5
E09000033	Westminster	48,724	23,723	48.7	48.2	49.1
E06000051	Shropshire	67,337	32,700	48.6	48.2	48.9
E08000028	Sandwell	39,552	19,164	48.5	48.0	48.9
E06000042	Milton Keynes	63,247	30,510	48.2	47.9	48.6
E08000036	Wakefield	61,543	29,680	48.2	47.8	48.6
E06000010	Kingston upon Hull, City of	17,074	8,219	48.1	47.4	48.9
E06000055	Bedford	31,728	15,205	47.9	47.4	48.5
E06000049	Cheshire East	52,794	25,264	47.9	47.4	48.3
E10000011	East Sussex	118,596	56,747	47.8	47.6	48.1
E08000009	Trafford	38,971	18,629	47.8	47.3	48.3
E06000044	Portsmouth	25,966	12,359	47.6	47.0	48.2
E06000059	Dorset	51,066	24,250	47.5	47.1	47.9
E08000023	South Tyneside	33,636	15,962	47.5	46.9	48.0
E10000030	Surrey	74,960	35,532	47.4	47.0	47.8
E06000015	Derby	62,407	29,315	47.0	46.6	47.4
E06000032	Luton	48,454	22,742	46.9	46.5	47.4
E08000008	Tameside	42,845	20,077	46.9	46.4	47.3
E10000008	Devon	105,836	49,495	46.8	46.5	47.1
E09000013	Hammersmith and Fulham	43,237	20,205	46.7	46.3	47.2
E09000007	Camden	44,662	20,798	46.6	46.1	47.0
E10000023	North Yorkshire	160,704	74,128	46.1	45.9	46.4
E09000004	Bexley	41,045	18,789	45.8	45.3	46.3
E08000003	Manchester	36,987	16,930	45.8	45.3	46.3
E10000028	Staffordshire	99,238	45,042	45.4	45.1	45.7
E08000013	St. Helens	35,045	15,868	45.3	44.8	45.8
E08000011	Knowsley	31,100	14,066	45.2	44.7	45.8
E06000058	Bournemouth, Christchurch and Poole	43,888	19,839	45.2	44.7	45.7
E06000020	Telford and Wrekin	34,384	15,444	44.9	44.4	45.4
E06000009	Blackpool	28,193	12,621	44.8	44.2	45.3
Unknown	Unknown	7,197	3,217	44.7	43.6	45.9
E10000002	Buckinghamshire	136,674	61,016	44.6	44.4	44.9

For peer review only - http://bmjopen.bmj.com/site/about/guidelines.xhtml

E10000032	West Sussex	90,033	40,022	44.5	44.1	44.8
E06000006	Halton	26,863	11,753	43.8	43.2	44.3
E06000052	Cornwall	48,099	20,877	43.4	43.0	43.8
E06000050	Cheshire West and Chester	40,408	17,537	43.4	42.9	43.9
E06000035	Medway	60,300	26,064	43.2	42.8	43.6
E10000020	Norfolk	161,582	69,173	42.8	42.6	43.1
E06000017	Rutland	6,741	2,862	42.5	41.3	43.6
E09000006	Bromley	75,672	31,841	42.1	41.7	42.4
E10000016	Kent	347,229	145,984	42.0	41.9	42.2
E09000008	Croydon	29,612	12,399	41.9	41.3	42.4
E09000011	Greenwich	32,488	13,547	41.7	41.2	42.2
E06000014	York	20,330	8,385	41.2	40.6	41.9
E08000027	Dudley	78,489	32,316	41.2	40.8	41.5
E06000026	Plymouth	28,855	11,707	40.6	40.0	41.1
E08000012	Liverpool	99,029	40,074	40.5	40.2	40.8
E10000018	Leicestershire	172,437	69,666	40.4	40.2	40.6
E08000024	Sunderland	47,131	18,370	39.0	38.5	39.4
E09000020	Kensington and Chelsea	35,607	13,811	38.8	38.3	39.3
E06000007	Warrington	48,004	18,287	38.1	37.7	38.5
E08000031	Wolverhampton	32,226	12,091	37.5	37.0	38.0
E08000010	Wigan	53,620	19,638	36.6	36.2	37.0
E09000021	Kingston upon Thames	32,087	11,529	35.9	35.4	36.5
E06000041	Wokingham	5,010	1,621	32.4	31.1	33.7
E08000037	Gateshead	49,663	14,497	29.2	28.8	29.6
E06000037	West Berkshire	16,235	4,376	27.0	26.3	27.6
E08000032	Bradford	82,669	20,791	25.1	24.9	25.4

Supplementary Table 10: Number of invitations recorded for attendees and non-attendees

Number of invitations	Attendees n(%)	Non-attendees n(%)
0	1,672,844 (32.8)	51,739 (1.1)
1	2,577,581 (50.5)	3,369,517 (73.4)
2	677,783 (13.3)	783,472 (17.1)
> 2	174,550 (3.4)	387,493 (8.4)
**TOTAL**	**5,102,758 (100.0)**	**4,592,221 (100.0)**

For peer review only - http://bmjopen.bmj.com/site/about/guidelines.xhtml

Supplementary Table 11: Invitations by financial year

Proportion of attendees and non-attendees with an invitation recorded

Year	Attendees with invitation	% attendees	Non-attendees with invitation	% non-attendees
2012/13	468,766	63.1	718,527	99.0
2013/14	619,559	64.3	824,429	98.9
2014/15	763,444	67.2	1,016,155	99.0
2015/16	790,731	69.2	999,178	98.7
2016/17	787,414	70.4	982,193	98.8
**TOTAL**	**3,429,914**	**67.2**	**4,540,482**	**98.9**

Supplementary Table 12: Completeness of risk factor measurement

Percentage of NHSHC attendees and non-attendees with recorded risk factor measurements (restricted to 15-month window around index date for attendees and unrestricted for non-attendees)

Group	CVD risk score	Body Mass Index	Physical Activity (GPPAQ)	Alcohol (Audit C)	Fasting glucose	HbA1C	Smoking Status	Cholesterol (HDL)	Cholesterol (total)	Diastolic BP	Systolic BP
Attendees	79.7%	96.3%	64.5%	38.3%	18.2%	36.6%	95.7%	87.2%	93.6%	95.7%	95.8%
Non-attendees	30.4%	55.0%	13.9%	16.7%	15.1%	37.5%	71.8%	47.3%	50.0%	76.3%	76.3%

For peer review only

Supplementary Table 13: Statin prescription rates

New statin (any dose) prescriptions among the subset (60.4%) of NHSHC attendees in whom medication data was available

Group	Attendees (n)	Prescribed a statin (n)	Proportion (%)
CVD score <10%	1,910,919	63,227	3.3
10-19.9%	532,046	83,279	15.7
≥20%	132,366	51,691	39.1
No CVD score	504,374	55,630	11.0
**Overall total**	**3,079,705**	**253,827**	**8.2**

For peer review only

The RECORD statement – checklist of items, extended from the STROBE statement, that should be reported in observational studies using routinely collected health data.

	Item No.	STROBE items	Location in manuscript where items are reported	RECORD items	Location in manuscript where items are reported
**Title and abstract**					
	1	(a) Indicate the study's design with a commonly used term in the title or the abstract (b) Provide in the abstract an informative and balanced summary of what was done and what was found	a) title<br>b) abstract	RECORD 1.1: The type of data used should be specified in the title or abstract. When possible, the name of the databases used should be included.<br><br>RECORD 1.2: If applicable, the geographic region and timeframe within which the study took place should be reported in the title or abstract.<br><br>RECORD 1.3: If linkage between databases was conducted for the study, this should be clearly stated in the title or abstract.	1.1 Title<br>1.2 Title<br>1.3 n/a
**Introduction**					
Background rationale	2	Explain the scientific background and rationale for the investigation being reported	Introduction		
Objectives	3	State specific objectives, including any prespecified hypotheses	Introduction		
**Methods**					
Study Design	4	Present key elements of study design early in the paper	Study design		
Setting	5	Describe the setting, locations, and relevant dates, including periods of recruitment, exposure, follow-up, and data collection	Study setting		

For peer review only

Participants	6	*(a) Cohort study* - Give the eligibility criteria, and the sources and methods of selection of participants. Describe methods of follow-up<br>*Case-control study* - Give the eligibility criteria, and the sources and methods of case ascertainment and control selection. Give the rationale for the choice of cases and controls<br>*Cross-sectional study* - Give the eligibility criteria, and the sources and methods of selection of participants<br><br>*(b) Cohort study* - For matched studies, give matching criteria and number of exposed and unexposed<br>*Case-control study* - For matched studies, give matching criteria and the number of controls per case	Cross-sectional Study population	RECORD 6.1: The methods of study population selection (such as codes or algorithms used to identify subjects) should be listed in detail. If this is not possible, an explanation should be provided.<br><br>RECORD 6.2: Any validation studies of the codes or algorithms used to select the population should be referenced. If validation was conducted for this study and not published elsewhere, detailed methods and results should be provided.<br><br>RECORD 6.3: If the study involved linkage of databases, consider use of a flow diagram or other graphical display to demonstrate the data linkage process, including the number of individuals with linked data at each stage.	6.1 Figure 1 & Supplement<br>6.2 Because the extract consists only of those with NHSHC codes, we are unable to carry out validation studies. Instead we present completeness of data.<br>6.3 N/A
Variables	7	Clearly define all outcomes, exposures, predictors, potential confounders, and effect modifiers. Give diagnostic criteria, if applicable.	Methods. Variables	RECORD 7.1: A complete list of codes and algorithms used to classify exposures, outcomes, confounders, and effect modifiers should be provided. If these cannot be reported, an explanation should be provided.	7.1 Supplement
Data sources/ measurement	8	For each variable of interest, give sources of data and details of methods of assessment (measurement).<br>Describe comparability of assessment methods if there is more than one group	Methods- variables and Supplement		

For peer review only

Bias	9	Describe any efforts to address potential sources of bias	Methods- data presentation		
Study size	10	Explain how the study size was arrived at	Methods Figure 1		
Quantitative variables	11	Explain how quantitative variables were handled in the analyses. If applicable, describe which groupings were chosen, and why	Methods- Variables		
Statistical methods	12	(a) Describe all statistical methods, including those used to control for confounding<br>(b) Describe any methods used to examine subgroups and interactions<br>(c) Explain how missing data were addressed<br>(d) *Cohort study* - If applicable, explain how loss to follow-up was addressed<br>*Case-control study* - If applicable, explain how matching of cases and controls was addressed<br>*Cross-sectional study* - If applicable, describe analytical methods taking account of sampling strategy<br>(e) Describe any sensitivity analyses	Methods- data presentation		
Data access and cleaning methods		..		RECORD 12.1: Authors should describe the extent to which the investigators had access to the database population used to create the study population.	12.1 methods- study setting<br>12.2 methods – data management and cleaning & Figure 1, Supplement

For peer review only

				RECORD 12.2: Authors should provide information on the data cleaning methods used in the study.	
Linkage		..		RECORD 12.3: State whether the study included person-level, institutional-level, or other data linkage across two or more databases. The methods of linkage and methods of linkage quality evaluation should be provided.	12.3 – Methods-Study design individual level data<br>n/a on linkage
**Results**					
Participants	13	(a) Report the numbers of individuals at each stage of the study (*e.g.*, numbers potentially eligible, examined for eligibility, confirmed eligible, included in the study, completing follow-up, and analysed)<br>(b) Give reasons for non-participation at each stage.<br>(c) Consider use of a flow diagram	a) Figure 1<br>& Overall uptake by year<br>b) figure 1<br>c) Figure 1	RECORD 13.1: Describe in detail the selection of the persons included in the study (*i.e.,* study population selection) including filtering based on data quality, data availability and linkage. The selection of included persons can be described in the text and/or by means of the study flow diagram.	Figure 1
Descriptive data	14	(a) Give characteristics of study participants (*e.g.*, demographic, clinical, social) and information on exposures and potential confounders<br>(b) Indicate the number of participants with missing data for each variable of interest<br>(c) *Cohort study* - summarise follow-up time (*e.g.*, average and total amount)	a) Table 1<br>b) Table 1		
Outcome data	15	*Cohort study* - Report numbers of outcome events or summary measures over time<br>*Case-control study* - Report numbers in each exposure	No outcome reported – described data for attendees and non-attendees		

For peer review only

		category, or summary measures of exposure<br>*Cross-sectional study* - Report numbers of outcome events or summary measures			
Main results	16	(a) Give unadjusted estimates and, if applicable, confounder-adjusted estimates and their precision (e.g., 95% confidence interval). Make clear which confounders were adjusted for and why they were included<br>(b) Report category boundaries when continuous variables were categorized<br>(c) If relevant, consider translating estimates of relative risk into absolute risk for a meaningful time period	a) n/a<br>b) Supplement<br>c) n/a		
Other analyses	17	Report other analyses done—e.g., analyses of subgroups and interactions, and sensitivity analyses	n/a		
**Discussion**					
Key results	18	Summarise key results with reference to study objectives	Discussion		
Limitations	19	Discuss limitations of the study, taking into account sources of potential bias or imprecision. Discuss both direction and magnitude of any potential bias	Limitations	RECORD 19.1: Discuss the implications of using data that were not created or collected to answer the specific research question(s). Include discussion of misclassification bias, unmeasured confounding, missing data, and changing eligibility over time, as they pertain to the study being reported.	
Interpretation	20	Give a cautious overall interpretation of results considering objectives,	Discussion, Conclusion		

For peer review only

		limitations, multiplicity of analyses, results from similar studies, and other relevant evidence			
Generalisability	21	Discuss the generalisability (external validity) of the study results	n/a		
**Other Information**					
Funding	22	Give the source of funding and the role of the funders for the present study and, if applicable, for the original study on which the present article is based	Funding		
Accessibility of protocol, raw data, and programming code		..		RECORD 22.1: Authors should provide information on how to access any supplemental information such as the study protocol, raw data, or programming code.	Code on GITHUB

*Reference: Benchimol EI, Smeeth L, Guttmann A, Harron K, Moher D, Petersen I, Sørensen HT, von Elm E, Langan SM, the RECORD Working Committee. The REporting of studies Conducted using Observational Routinely-collected health Data (RECORD) Statement. *PLoS Medicine* 2015; in press.

*Checklist is protected under Creative Commons Attribution (CC BY) license.

For peer review only

BMJ Open

An evaluation of the uptake and delivery of the NHS Health Check Programme in England, using primary care data from 9.5 million people: A cross-sectional study

Journal:	*BMJ Open*
Manuscript ID	bmjopen-2020-042963.R1
Article Type:	Original research
Date Submitted by the Author:	30-Sep-2020
Complete List of Authors:	Patel, Riyaz; University College London, Institute of Cardiovascular Science<br>Barnard, Sharmani; Public Health England<br>Thompson, Katherine; Public Health England<br>Lagord, Catherine; Public Health England<br>Clegg, Emma; Public Health England<br>Worrall, Robert; NHS Digital<br>Evans, Tim; Public Health England<br>Carter, Slade; Public Health England<br>Flowers, Julian; Public Health England<br>Roberts, Dave; NHS Digital<br>Nuttall, Michaela; Public Health England<br>Samani, Nilesh; University of Leicester, Department of Cardiovascular Sciences<br>Robson, John; Queen Mary University of London, Centre for Primary Care and Public Health<br>Kearney, Matt; UCL Partners<br>Deanfield, John; University College London, Institute of Cardiovascular Science<br>Waterall, Jamie; Public Health England
**Primary Subject Heading**:	Public health
Secondary Subject Heading:	Cardiovascular medicine, Public health
Keywords:	VASCULAR MEDICINE, PUBLIC HEALTH, PREVENTIVE MEDICINE

SCHOLARONE™
Manuscripts

I, the Submitting Author has the right to grant and does grant on behalf of all authors of the Work (as defined in the below author licence), an exclusive licence and/or a non-exclusive licence for contributions from authors who are: i) UK Crown employees; ii) where BMJ has agreed a CC-BY licence shall apply, and/or iii) in accordance with the terms applicable for US Federal Government officers or employees acting as part of their official duties; on a worldwide, perpetual, irrevocable, royalty-free basis to BMJ Publishing Group Ltd ("BMJ") its licensees and where the relevant Journal is co-owned by BMJ to the co-owners of the Journal, to publish the Work in this journal and any other BMJ products and to exploit all rights, as set out in our licence.

The Submitting Author accepts and understands that any supply made under these terms is made by BMJ to the Submitting Author unless you are acting as an employee on behalf of your employer or a postgraduate student of an affiliated institution which is paying any applicable article publishing charge ("APC") for Open Access articles. Where the Submitting Author wishes to make the Work available on an Open Access basis (and intends to pay the relevant APC), the terms of reuse of such Open Access shall be governed by a Creative Commons licence – details of these licences and which Creative Commons licence will apply to this Work are set out in our licence referred to above.

Other than as permitted in any relevant BMJ Author's Self Archiving Policies, I confirm this Work has not been accepted for publication elsewhere, is not being considered for publication elsewhere and does not duplicate material already published. I confirm all authors consent to publication of this Work and authorise the granting of this licence.

For peer review only

Title: An evaluation of the uptake and delivery of the NHS Health Check Programme in England, using primary care data from 9.5 million people: A cross-sectional study

Running Title: NHS Health Check Programme

Author Block:

Riyaz S. Patel,[1, 2,]* BHF Clinical Intermediate Fellow, Sharmani Barnard,[3,]* Statistician, Katherine Thompson,[3] Head of CVD Prevention programme, Catherine Lagord,[3] Analyst, Emma Clegg,[3] Analyst, Robert Worrall, [4] Analyst, Tim Evans,[3] Analyst, Slade Carter,[3] Programme Manager, Julian Flowers,[3] Head of Public Health Data Science, Dave Roberts,[4] Head of Primary Care Information, NHS Digital, Data Extract Advisory Committee (DEAC),[#] Michaela Nuttall,[3] Clinical Adviser, Nilesh J Samani,[5] Professor of Cardiology, John Robson,[6]Reader in Primary Care , Matt Kearney,[7] GP and Deputy Managing Director UCL Partners Academic Health Science Network, John Deanfield,[1,3,]* Professor of Cardiology, Jamie Waterall [3,]* Deputy Chief Nurse

*These authors contributed equally; [#]DEAC membership included at the end of the paper

Affiliations:

1. Institute of Cardiovascular Sciences, University College London, London, UK,
2. Bart's Heart Centre, St Bartholomew's Hospital, West Smithfield, London, UK
3. Public Health England, Wellington House, 133-155 Waterloo Road, London, UK
4. NHS Digital, 1 Trevelyan Square, Boar Lane, Leeds, UK
5. Department of Cardiovascular Sciences University of Leicester and NIHR Leicester Biomedical Research Centre, Glenfield Hospital, Leicester, UK
6. Centre for Primary Care and Public Health, Queen Mary University of London, London
7. UCL Partners, 3rd Floor, 170 Tottenham Court Road, London, UK

Corresponding Author:

Dr Riyaz Patel, https://orcid.org/0000-0003-4603-2393

222 Euston Rd, Institute of Cardiovascular Sciences, University College London, London, NW1 2DA

Email: Riyaz.patel@ucl.ac.uk Telephone: +44 (0) 20 3549 5332

Word Count:

Abstract: 299; Main Text: 4439

For peer review only - http://bmjopen.bmj.com/site/about/guidelines.xhtml

For peer review only

Abstract:

Objectives: To describe the uptake and outputs of the NHS Health Check (NHSHC) programme in England.

Design: Observational study

Setting: National primary care data extracted directly by NHS Digital from 90% of General Practices (GP) in England.

Participants: Individuals aged 40-74 years, invited to or completing a NHSHC between 2012 and 2017, defined using primary care Read codes.

Intervention: The NHSHC, a structured assessment of non-communicable disease risk factors and 10-year cardiovascular disease (CVD) risk, with recommendations for behavioural change support and therapeutic interventions.

Results: During the 5-year cycle, 9,694,979 individuals were offered an NHSHC and 5,102,558 (52.6%) took up the offer. There was geographical variation in uptake between local authorities across England ranging from 25.1% to 84.7%. Invitation methods changed over time to incorporate greater digitalisation, opportunistic delivery and delivery by third party providers.

The population offered an NHSHC resembled the English population in ethnicity and deprivation characteristics. Attendees were more likely to be older and female, but were similar in terms of ethnicity or deprivation, compared to non-attendees. Among attendees risk factor prevalence reflected population survey estimates for England. Where a CVD risk score was documented, 25.9% had a 10-year CVD risk ≥10%, of which 20.3% were prescribed a statin. Advice, information and referrals were coded as delivered to over 2.5 million individuals identified to have risk factors.

Conclusion: This national analysis of the NHSHC programme using primary care data from over 9.5M individuals offered a check, reveals an uptake rate of over 50% and no significant evidence of inequity by ethnicity or deprivation. To maximise the anticipated value of the NHSHC, we suggest continued action is needed to invite more eligible people for a check, reduce geographical variation in uptake, prioritise engagement with non-attendees, and promote greater use of evidence-based interventions especially where risk is identified.

Keywords: Cardiovascular Disease Prevention; NHS Health Checks; Cardiovascular Risk; Public Health

For peer review only - http://bmjopen.bmj.com/site/about/guidelines.xhtml

Strengths and Limitations:

- A comprehensive national level snapshot of NHS Health Check (NHSHC) programme, derived from primary care records, and which underpins the recently released NHSHC data dashboard
- Academic and public health collaboration with full access to half a billion records for over 9.5M people offered an NHSHC between 2012-2017
- This first data analysis reports on elements relating to uptake, implementation, process and delivery of NHSHCs, the sociodemographic and risk factor profile of both those who did and did not attend a check and rates of advice, referrals and statin prescriptions delivered as part of the check
- The data was restricted to people with an NHSHC activity code, and thus we were unable to quantify the full eligible population to determine coverage and the gap in programme reach
- Missing data and varying volume of completeness of risk factor measures limits comparisons between attendees and non-attendees

For peer review only

Introduction

Cardiovascular disease (CVD) remains a major public health priority in England.[1] To address this the Government introduced an ambitious programme of vascular checks in 2009, for people aged 40-74, delivered by England's National Health Service (NHS).[2] NHS Health Checks (NHSHC) sought to address the key risk factors driving the health and economic burden from vascular disease,[3] with early modelling suggesting that each year NHSHCs would prevent 9,500 heart attacks and strokes, 4,000 new cases of diabetes and identify at least 25,000 people with existing undiagnosed diabetes or kidney disease before they developed complications.[2 4] Furthermore, with the same vascular risk factors increasingly recognised as contributing to other conditions like dementia, preventable cancers, and liver disease,[3] the programme has assumed an even greater importance in the prevention of non-communicable diseases.[5 6 7]

Over a decade on, the NHSHC, is now an embedded systematic and nationwide detailed risk assessment, awareness and management programme in England. Since 2013, following legislation, local authorities have a statutory obligation to make provision for all eligible people to have an NHSHC every five years.[8] However, concerns have been raised that delivery and practical implementation of such a programme presents a paradoxical risk of increasing health inequality if implemented in a way which does not systematically prioritise equity of access, outputs and outcomes. Furthermore, the absence of convincing randomised clinical trial evidence about the effectiveness of such programmes, has further prompted ongoing scrutiny and questions around its delivery, uptake, impact and cost-effectiveness.[9]

In response, the number of studies evaluating the delivery and impact of the NHSHC continue to grow but have shown variable results.[10] This may be a result of heterogeneity in programme delivery, small sample sizes, use of national data before NHSHCs were passed into law, or variation in local coding practices. In addition, some studies have drawn conclusions from analyses of the Clinical Practice Research Datalink (CPRD), or QResearch databases,[11] which although a representative and important primary care research resource, are limited by being restricted to volunteer practices utilising specific electronic health record systems with some under-representation in Northern England.[11 12]

To overcome some of these difficulties and provide a contemporaneous overview of the NHSHC programme in England, we sought to analyse the largest NHSHC national primary care dataset to be extracted to date, drawing on data for almost ten million individuals and half a billion records, specifically extracted for this purpose and one which underpins the recently released NHSHC data dashboard.[13] A series of reports will examine the delivery of the programme, prevention opportunities

For peer review only - http://bmjopen.bmj.com/site/about/guidelines.xhtml

identified and the impact of the NHSHC. The objectives of this first paper are to describe the data extract and to provide an overview of the programme, reporting on: (i) its uptake, process and delivery, (ii) the sociodemographic and risk factor profiles of attendees and non-attendees and (iii) advice, referrals and statin prescriptions following the check.

Methods

Study Setting

Public Health England (PHE) is responsible for national oversight and implementation support of the NHSHC programme. PHE worked with NHS Digital (NHSD) to develop business rules for a data extract of all NHSHC coding activity to allow England wide monitoring of the NHSHC.[14] A data extract advisory committee (DEAC) was set up to guide use of the data extract. Full details of the scope and composition of the committee are available online.[15]

Study Design

We conducted a retrospective descriptive cross-sectional study of all individuals who were offered an NHSHC, using individual-level participant data. We describe the data extraction before defining the study population. The study design and report conform to RECORD recommendations for reporting of observational studies using routinely collected data.[16]

Data Extraction & Criteria

Data was extracted from 6,524 (90%) of the 7,216 General Practices participating in the General Practice Data Extraction Service (GPES),[17] after excluding individuals who had opted out of their data being used for purposes other than direct patient care. [18]

The inclusion criteria for the data extract, was a primary care Read code for any one of the following NHSHC activities: invitation, completion, non-attendance, inappropriate, commenced or declined (prior to 1st April 2018). Full details of the Read codes used for defining NHSHC activity is available in **Supplementary Table 1**.

The data extracted for each individual included socio-demographic characteristics, risk factors for cardiovascular disease, diagnostic tests requested following the check, and interventions including advice and referrals. CVD diagnoses and medication data were also extracted from three out of the four GP clinical IT systems providers, corresponding to 60% of practices. Data extraction for all variables were restricted to time windows around the individual’s contact with the NHSHC programme

For peer review only - http://bmjopen.bmj.com/site/about/guidelines.xhtml

as specified in the business rules for extraction, listed in **Supplementary Table 2**. Data for CVD diagnoses and a broader range of medications will be presented in subsequent papers.

At the time of extraction in 2018, the business rules limited the upper age limit to 75 years for each year. As a result, due to the rolling nature of the programme, this resulted in missing data for the 70-74 age group, most of whom turned 75 during the 5-year cycle. Thus, the maximum age of patients in the extract is 69 for the financial year 2012/13, compared to 73 in 2016/17. The final extraction consisted of 12,151,896 patient records with NHSHC activity coding recorded up until 31st March 2018. Data management and data cleaning details are provided in **Supplementary Methods** and **Supplementary Table 3**.

<u>Study Population</u>

NHSHCs are offered to individuals aged 40-74 years and without any of the following conditions: hypertension, diabetes mellitus, familial hypercholesterolaemia, coronary heart disease, heart failure, atrial fibrillation, stroke or transient ischaemic attack, peripheral arterial disease, chronic kidney disease and those already on statins or known to have a 10-year CVD risk of ≥ 20%.[5]

The study population for this analysis was derived from the data extract described above for any NHSHC coded activity. From this group, individuals (1) with NHSHC activity coded outside the study window, (2) aged <40 years at the time of activity, and (3) coded by the GP as inappropriate for an NHSHC were then additionally excluded. The final study population thus included only those people offered an NHSHC (invited or completed). **Figure 1** presents the study extract and population flow chart.

<u>Definitions and Study Variables</u>

Individuals were categorised as either NHSHC attendees if they had a Read code for a completed check within the 5-year period, or a non-attendee if they did not. Uptake of the programme was defined as the proportion of the total study population who attended.

An index date was generated from the date of an individual's primary NHSHC activity to identify age and the most relevant risk factor measurements for each patient. Risk factor and clinical measurements were selected for analysis if they occurred on the index date. Otherwise we took the closest recording within pre-defined time windows set by the DEAC. Statin prescriptions that occurred on or after the index data among attendees with no data for previous statin prescription were

For peer review only - http://bmjopen.bmj.com/site/about/guidelines.xhtml

selected. A full list of variables, Read codes used to define variables, time windows and coding algorithms is available in **Supplementary Table 4**.

Further details on study variable definitions and thresholds are provided in **Supplementary Methods** and **Supplementary Tables 4-8.**

Data Presentation

Statistical tests were not used for comparison because the amount of missing data between groups varies, thereby preventing meaningful comparisons and the large size of the study population permits the identification of very small differences between groups. Instead, we highlighted the size of differences between groups and interpreted it in relation to the missing data. Where appropriate, we presented data for attendees and non-attendees. Data for uptake, invitation type and third-party provider is presented by financial year, to describe changes over time. Data on uptake is also presented by local authority for geographical comparisons. To minimise bias, we include missing data details in all tables and figures.

Patient and Public Involvement

PHE developed an information notice for patients, including an easy read version, explaining how their personal data would be used and the purpose of the research project. Membership of the Data Extract Advisory Committee overseeing the use of the NHS Health Check dataset, including the development of this study, its design and outcomes, includes a patient representative. Study results will not be disseminated to individuals whose data is used but the collective analysis presented here will be shared publicly once published.

Ethical Approval

A Direction from the Secretary of State for Health and Social Care instructed NHS Digital with the legal requirement to carry out the NHSHC data extract.[19] This study was subject to an internal review by the Research Support and Governance Office in PHE to ensure that it was fully compliant with the UK Policy Framework for Health and Social Care Research (2017) and with all other current regulatory requirements. The review also covered all ethical considerations. No ethical issues were identified and thus review by an ethics committee was not required (Personal communication between Katherine Thomson & PHE Research Support Governance Office, 2019).

For peer review only - http://bmjopen.bmj.com/site/about/guidelines.xhtml

Results

NHSHC Uptake

Overall Uptake by Year

Between 1st April 2012 and 31st March 2017, 9,694,979 individuals aged 40 to 74 years were offered an NHSHC in England. Of these 5,102,758 (52.6%) completed a check. Uptake by financial year is presented in **Table 1**. Uptake remained > 50% throughout the five years of programme delivery. The number of individuals offered a NHSHC increased from just under 1.5M in 2012/13, to 1.8M the year after, plateauing thereafter at approximately 2.1M each year after that, **Table 1**.

Geographical variation in uptake of offers

Across England, uptake rates varied by region, as presented in **Figure 2A.** The highest uptake of offers over the five-year cycle was in Hampshire (84.7%) and the lowest in Bradford (25.1%). Data for uptake by upper tier local authority (UTLA) is available in **Supplementary Table 9**. Variation in uptake in London is shown in **Figure 2B.** Central and north London local authorities had higher rates of uptake, with lower rates in the south east.

Process and Delivery

Invitation Frequency

Of the 9,694,979 individuals in the study population with codes for NHSHC activity, 7,970,396 (82.2%) had a record of at least one NHSHC invitation. **Supplementary Table 10** presents the number of recorded invitations for attendees and non-attendees (recording by each financial year is available in **Supplementary Table 11**).

Among the 5,102,758 attendees, almost a third (32.8%), had no invitation code recorded but still had a completed NHSHC recorded. The remaining two thirds (3,429,914) had an invitation recorded, with 50.5% having one invitation, and 16.7% two or more. Among these attendees coded as invited, 590,869 (17.2%) received an invitation on the same date as the NHSHC and were thus assumed to be opportunistic rather than planned. Among those with an invitation in advance of the NHSHC (82.8%; n= 2,839,045), the median number of days between recording of their first invitation and a completed NHSHC was 42 (IQR 21, 90) days.

Among non-attendees, 98.9% had a formal invitation record, with a quarter (25.5%) having two or more invitations. The remaining 1.1% of non-attendees had Read codes for declining or not attending a check, **Supplementary Table 1**.

For peer review only - http://bmjopen.bmj.com/site/about/guidelines.xhtml

Invitation Type

Among both attendees and non-attendees, the most common invitation type was a letter, however other forms of invitations, including text messaging, increased with each year of the programme. **Supplementary Figure 1** presents the type of invitation by financial year among attendees and non-attendees.

Delivery

Among all attendees within the five-year timeframe, 3.0% had a clinical code to indicate that their NHSHC was completed by a third party. This increased gradually from 1.2% in the first year to 4.1% in the final year.

Characteristics of Invitees

Socio-Demographic Characteristics

Table 2 presents the socio-demographic characteristics of the study population and the characteristics of the general population according to ONS modelled estimates. The population offered an NHSHC was representative of the general population of people aged 40-74 years in terms of sex and deprivation index although they were younger relative to the age distribution of the general population (age <55: 62.2% v 49.7%). Those who were offered an NHSHC also closely resembled the ethnic makeup of the general population for most ethnicities, except for people self-reporting as white or black Caribbean who appeared underrepresented, although 16.7% of data for ethnicity was missing.

Attendees differed from non-attendees. More attendees were female (54.7%) compared to non-attendees (47.5%; general population 50.9%). There were also notable differences by age. Most attendees were < 55 years as they constituted the largest group of eligible people, but individuals ≥55 years had higher rates of attendance after invitation. For ethnic group comparisons, a large proportion of missing data for non-attendees (27.8%) compared to attendees (6.8%) limits interpretation, but where data were available and compared to the general population, ethnic minority groups appeared to be better represented among attendees than non-attendees, **Table 2**.

Deprivation indices indicate few differences between attendees and non-attendees, except at the extreme ends of the index of multiple deprivation (IMD) spectrum, where there were slightly more attendees from the most affluent areas (Decile 10: 11.0% v 10.0%) and slightly less attendees from the most deprived areas (Decile 1: 8.2% vs 9.4%). Finally, although the numbers were small, there was

For peer review only

no evidence to indicate that people with severe mental illness, physical or cognitive disability were under-represented among attendees, **Table 2**.

Risk Factors

Overall, completeness of data for common risk factors measurements including systolic blood pressure (BP) (95.8%), smoking (95.7%), BMI (96.3%) and total cholesterol (93.6%) was high in attendees, in contrast to recording of physical activity (64.5%), blood glucose (18.2%), HbA1C (36.6%) and alcohol (38.3%). A CVD risk score was formally documented for 79.7% of attendees (**Figure 3** and **Supplementary Table 12**). Family history data was only recorded where a positive finding was present, making it difficult to estimate how much data was missing or was assessed and was negative. Completeness of most, but not all risk factors, was lower among non-attendees, with the exception of diabetes risk measurements which were similarly low in both groups.

Figure 4 shows the proportion of all individuals identified as having each CVD risk factor among attendees and non-attendees and with respect to missingness of data. Among attendees, where missingness was low, we identified 24.5% with hypertension, while 23.8% were obese and 16% were current smokers. Where a 10-year CVD risk score was documented in the primary care record (79.7% of attendees), just over a quarter (25.9%) were identified as high risk, with a score of ≥ 10%.

Advice, Referrals and Interventions

Advice, information and referral for an intervention following an NHSHC was recorded almost six million times for all attendees, and more than 2.5 million times for individuals with elevated CVD risk factors, **Table 3.** Among all attendees, 16.0% were coded to have received general lifestyle and behavioural advice, just over a fifth were given formal advice on diet, and almost a third on physical activity. Among those whose alcohol use puts them above low risk, more than a third were directed to alcohol treatment services. Almost half of all current smokers were directed to smoking cessation services and 19.6% of those who had a BMI ≥ 30 were directed to weight loss and obesity services.

Statin Prescriptions

Information on a new statin prescription, occurring on or after NHSHC completion, was available for 60.4% of all attendees (n=3,079,705, see Methods). Overall a statin was prescribed for 8.2% of these attendees. Stratifying this group by CVD risk, revealed that a statin was prescribed in 20.3% of those with a 10-year CVD risk score ≥ 10% and in 39.1% of those with a CVD risk score of ≥20%. Among the 1,910,919 individuals with a CVD risk score <10%, 3.3% received a new statin prescription, while in the

For peer review only - http://bmjopen.bmj.com/site/about/guidelines.xhtml

remaining 504,374 with no CVD risk score recorded, 11.0% were prescribed a statin. **Supplementary Table 13.**

Assuming similar rates of statin prescription nationally, we estimate that of the 5,102,785 attendees in this study, up to 418,000 may have received a new statin prescription, with over half of these (n~213,000) prescribed to those identified at the NHSHC visit as being at >10% risk of CVD events.

Discussion

In the largest nationwide study of the NHS Health Check programme, using primary care data, we find that the checks been offered to over 9.5M people during a 5-year cycle up to 2017, with 52% of people taking up the offer. While we noted geographical variation in uptake rates, and an age and sex bias for attendance, we found little evidence of inequality in who was offered or who received an NHSHC by ethnicity or deprivation indices. Where an NHSHC was delivered, risk factors were identified at a similar rate to population estimates, with advice and referrals offered over 2.5M times to those with risk factors, along with 20% of those at highest risk receiving a new statin prescription as per guidelines. These insights into the evolving process and delivery of the NHSHC programme will support efforts to further enhance the value of the programme, especially for improving uptake rates, targeting those at greatest risk and maximising the use of available NCD & CVD risk reduction interventions.

Our key finding of a 52% uptake rate is slightly higher than previous studies, reporting around 48%.[10] This may be due to the larger, more nationally representative and contemporary data to which we had access, supported by the finding that uptake rates have steadily increased since 2012. Furthermore, we also found wide geographical variation, across the country and in London, possibly due to differing coding practices or invitation methods, which could skew findings from smaller studies or explain discordance with other reports of NHSHC activity.[20] However, an important difference that precludes direct comparison with other studies reporting on NHSHC reach is that our study was restricted to people who had an NHSHC code in their GP records, indicating either an invitation or completion of a check. As such we were unable to quantify coverage of the programme, i.e. how many eligible people were offered a check. Estimates from PHE, based on Office for National Statistics data minus the estimated number of people on existing disease registers suggests an eligible population of ~15.5 million.[20] Using this number and based on 5.1M having had a check we estimate that a further 6.5M in the same 5 year cycle would need to complete an NHSHC to achieve the original programme aspiration of 75% coverage.[4 8]

For peer review only - http://bmjopen.bmj.com/site/about/guidelines.xhtml

Some NHSHC providers have raised concerns that the programme may paradoxically increase health inequality by only attracting the worried well with more affluent and white people.[21] Reassuringly the data do not show gross differences in the offering or uptake of the programme. Firstly, those who were offered a NHSHC closely resemble the population of England, as measured through census data, with no differences by sex, ethnicity or deprivation indices. They were slightly younger overall, but this is likely because eligibility for an NHSHC falls with comorbidities which are frequently age related.[5] Secondly, although missing data on ethnicity limits definitive conclusions, ethnic minorities such as those from South Asia were equally if not more represented as reported by others.[22 23] Furthermore, while there were small differences at the extremes of deprivation deciles, overall there was no gross bias towards greater attendance by increasing affluence and previous mixed findings are likely due to regional variation, [22-24] while the similar uptake rates in those with physical disability or serious mental illness also indicates the programme is equitably delivered. There was however a notable bias towards more females and older people attending for a NHSHC compared to non-attendees, a finding also observed by others.[10 11 22 23]

Of note, despite older people being more likely to attend than not attend after having an offer of a NHSHC, proportionally 57% of all attendees were <55 years, higher than reports from other national evaluations of the programme.[11] This could be because our data was limited for the age 70-74 group or that more older people are excluded having been identified with comorbidities earlier in the programme cycle when these other studies reported. However, it may also indicate that younger people are motivated to understand their CVD risk and engage with care providers to address their longer term and lifetime risk, a finding we previously observed with the use of digital risk assessment tool.[25] The potential benefits of this earlier engagement with CVD risk, will need to be evaluated over the longer term.

An important benefit of the NHSHC programme has been improvements in risk factor and behaviour data recording, which can guide patient interventions and inform regional resource priorities. For core data items such as smoking status, data completeness was as high as 96%, while for alcohol and physical activity (measures which are contractually required as part of the NHSHC but not needed to calculate a person's 10-year CVD risk) was close to 65%. This contrasts with the high degree of missing data among non-attendees for most risk factors. The exception being blood glucose and HbA1C measurements which were similarly complete at low levels for both non-attendees and attendees. This may be because these tests are only performed in attendees at high diabetes risk, combined with parallel current or historical efforts to establish and maintain a diabetes disease register outside of the NHSHC. Where risk factors, were recorded, they reveal that prevalence in attendees is close to

For peer review only - http://bmjopen.bmj.com/site/about/guidelines.xhtml

those in the wider UK population.[3 26] A 10 year risk score was documented in 79.7% of all attendees. We anticipate that in the remaining ~20%, practitioners may have estimated the score using an online or other tool not integrated into the clinical system, which may have meant the score was discussed but not recorded, although it is possible some may not have calculated it at all. Overall, where a score was recorded over a quarter of all attendees were calculated to have a 10-year CVD risk score of ≥10%, the current threshold set by NICE to consider preventative interventions such as statin prescription.[27] Indeed, we found 20% of this population was newly prescribed a statin following the NHSHC. This figure was even higher at nearly 40% for those with a 10-year CVD risk score of ≥20%, an older NICE threshold for statin prescription. This is an encouraging finding, being higher than in earlier studies and approaching the national ambition of 45% for statin use in this very high risk group.[11 28] Our data also suggest that the NHSHC encounter prompted relevant non-statin interventions with over 2.5M people with risk factors being coded as having received advice, information or referrals. We note however that these figures may be an underestimate being entirely dependent on coding practices and availability of services by region. For example, the low referral rates for the diabetes prevention programme (DPP) are partly explained by the programme launching relatively recently in 2016, but also due to variation in its availability across England and the poor recording of referrals to the programme in the primary care record as reported by others.[29]

Limitations:

Despite being the largest national evaluation of the NHSHC programme, our study has some important limitations. Firstly, our data was restricted to people with an NHSHC activity code, and thus we were unable to quantify the full eligible population to determine coverage and the gap in programme reach. Although this is an aspiration for future analyses, it will require access to GP records for much of the population, raising important data governance and handling challenges. Secondly, we had substantial missing data, especially for the non-attendees, limiting our ability to make robust conclusions about differences in characteristics and risk between these groups. Also, our data extract did not include information on 10% of practices in GPES, which could have introduced a degree of bias in our estimates if the reasons for missing data were not random and related to participation in the NHSHC programme. Thirdly, important information on those >70 years was limited due to a business rule that led to loss of older people once they turned 75 for each year of the data extract. However, the proportionally smaller number of older people eligible for an NHSHC means our results are unlikely to have been impacted significantly. Fourthly, prescription data was only available from 60% of practices. The estimate for statin prescriptions derived from the available data however is likely valid and representative. Finally, we used a Read code to identify if an NHSHC took place. This, of course does

For peer review only - http://bmjopen.bmj.com/site/about/guidelines.xhtml

not provide any indication as to the extent or quality of the conversations around risk or the suitability of information given, upon which the full impact and value of an NHSHC is likely to depend.

Clinical Implications:

This analysis provides a national level overview of the NHSHC programme, against which local authorities and health care providers can benchmark local achievements. Used with the NHS Digital dashboard, this will enable local CVD risk strategies to be developed, to increase the invitation of eligible individuals not yet invited for an NHSHC, as well as targeting those who still do not attend even after invitation.[13] Importantly, we show that a national prevention programme to tackle NCDs is possible and population health can be targeted through routine health care. It represents a systematic approach to switching the conversation from illness to preventing disease and appears to have good engagement from the public so far. From the data, we observe that in England there remains a major challenge for reducing risk factors that impact multiple long-term chronic conditions. The programme appears to have been successful at promoting advice and guideline-based interventions. Although assessing the efficacy of these interventions on individual level behaviour change is challenging, further analysis of this large dataset will explore the impact on available metrics such as diagnosis rates and clinical outcomes.

Conclusion:

In this large-scale analysis of the NHSHC programme using national primary care data, we found that in recent years over half of all people offered a check have completed one. Although there was substantial variation between local authorities in uptake rates, we found little or no evidence of inequity in invitation processes or uptake. Furthermore, the programme has identified a high burden of risk among attendees, with correspondingly encouraging levels of guideline driven advice, referrals and statin prescriptions for the primary prevention of CVD. However, to achieve fully the anticipated benefits of the NHSHC programme, we highlight a need for continued efforts to invite more of the eligible population for an NHSHC, reduce geographical variation in uptake of offers, prioritise those who are not attending and to maximise the use of evidence-based interventions to support risk reduction. Subsequent research should provide more insight into how different delivery models influence outcomes.

For peer review only - http://bmjopen.bmj.com/site/about/guidelines.xhtml

Statements

Funding

RSP (FS/14/76/30933) and JD (BHF chair) were funded by the BHF. Data extraction and analysis were funded by PHE.

Acknowledgments

We would like to thank colleagues from PHE and NHS Digital who supported this work. We would also like to thank the patient and public representatives involved with this work, for their input.

Disclosures/ Competing Interests

All authors have completed the ICMJE uniform disclosure form at www.icmje.org/coi_disclosure.pdf and declare: no support from any organisation for the submitted work; RSP has received speaker fees and honoraria from Amgen, Sanofi and Bayer and research grant funding from Regeneron, for CVD prevention and cholesterol management; no other relationships or activities that could appear to have influenced the submitted work.

Copyright:

The Corresponding Author has the right to grant on behalf of all authors and does grant on behalf of all authors, a worldwide licence to the Publishers and its licensees in perpetuity, in all forms, formats and media (whether known now or created in the future), to i) publish, reproduce, distribute, display and store the Contribution, ii) translate the Contribution into other languages, create adaptations, reprints, include within collections and create summaries, extracts and/or, abstracts of the Contribution, iii) create any other derivative work(s) based on the Contribution, iv) to exploit all subsidiary rights in the Contribution, v) the inclusion of electronic links from the Contribution to third party material where-ever it may be located; and, vi) licence any third party to do any or all of the above.

Transparency Declaration:

The guarantors (RP, SB, KT and CL) affirm that this manuscript is an honest, accurate, and transparent account of the study being reported; that no important aspects of the study have been omitted; and that any discrepancies from the study as planned have been explained.

Data Sharing Statement

For peer review only - http://bmjopen.bmj.com/site/about/guidelines.xhtml

The legal basis for the data extract was a Secretary of State for Health and Social Care Direction. With DEAC approval PHE and NHS Digital have set up a process for dealing with information requests relating to the pseudonymised primary care data used in this paper. The purpose for using this data must be for the scope of work relating to the evaluation of the NHS Health Check in line with the requirements of the Direction.

Author Contributions

All authors contributed to conception of the study, study design, overall analysis plan and critically reviewed the final manuscript. Specifically in addition, RSP, SB and KT contributed to the statistical analysis plan, review of results and drafted and revised the final paper; SB, CL, EC, TE and RW obtained and analysed all data and contributed to drafting of the final manuscript; SC, JF and DR supported data extraction for the analysis and review of the final manuscript; MN, NS, JR critically reviewed and edited the paper; MK, JD, JW conceived the study; contributed to the analysis plan and critically reviewed the final manuscript.

For peer review only

Data Extract Advisory Committee for NHS Health Check data extraction (DEAC): membership as of April 2020

John Deanfield (Co-Chair), Senior advisor to Public Health England on cardiovascular disease prevention & UCL professor of cardiology; Matt Kearney (Co-Chair), Programme Director, UCL Partners Academic Health Science Network, GP; Andrew Hughes, Heart Intelligence, National Cardiovascular Intelligence Network, PHE; Bob Ruane, Patient representative; Catherine Lagord (secretariat), Analyst, CVD prevention team, PHE; Dave Roberts, Head of primary care information, NHS Digital; Emma Brezan, Senior Public Health Manager, Royal Borough of Greenwich; Ifeoma Onyia, Consultant in public health, Halton Borough Council; Jamie Waterall, National Lead for Cardiovascular Disease Prevention, PHE; John Robson, Clinical Reader in Primary Care Research & Development Queen Mary University of London; Julian Flowers, Head of public health data science, PHE; John Newton, Director of Health Improvement, PHE; Kate Cheema, Director of Health Intelligence, British Heart Foundation; Katherine Thompson, Head of Cardiovascular Disease Prevention, PHE; Kathryn Salt, Principal data manager, primary care domain, NHS Digital, Lorraine Oldridge, National lead, National Cardiovascular Intelligence Network, PHE; Michaela Nuttall, Deputy National Lead, CVD prevention team, PHE; Mohammed Vaqar, Health and Wellbeing Officer, CVD, PHE West Midlands; Nick Wareham, Director of the MRC Epidemiology Unit, University of Cambridge; Nilesh Samani, Professor of cardiology at University of Leicester, medical director, British Heart Foundation; Paul Cundy, GP and chair of the GPC IT subcommittee; Peter Green, Clinical Chair NHS Medway Clinical Commissioning Group; Peter Kelly, Centre director, PHE North East; Phil Koczan, Royal College of General Practitioners representative, Riyaz Patel, BHF Senior Lecturer at UCL and Consultant Cardiologist at UCLH and Barts Health NHS Trusts; Rob Aldridge, Associate Professor, Institute of Health Informatics, UCL; Robert Danks, Principal Information Analyst, Primary Care Domain, NHS Digital; Rob Worrall, Senior information analyst, primary care domain, NHS Digital; Sharmani Barnard, Statistician, PHE; Tim Evans, Stroke Intelligence, National Cardiovascular Intelligence Network, PHE; Zain Chaudhry, NHS England and NHS Improvement;

For peer review only - http://bmjopen.bmj.com/site/about/guidelines.xhtml

Figure Legends

Figure 1: Study extract and study population flow chart. The study population inclusion dates (1st April 2012 to 31st March 2017) reflect a snapshot of the five-year rolling programme from April 2012, when all trusts commissioning primary care in England had implemented the programme.
*NHS Health Check activity refers to any interaction that a patient may have had with the NHS Health Check programme. This includes if a patient was invited to, commenced, completed, declined, did not attend, or was inappropriate for, the NHS Health Check. More details are provided in Supplementary Table 1

Figure 2: Variation in NHSHC uptake across (A) England and (B) London. Uptake rates shown as % of people taking up an offer of a check, between 2012/3 to 2016/17, by Upper Tier Local Authority of the individuals' usual residence

Figure 3: Completion of risk factor measurements for attendees and non-attendees (2012/13 - 2016/17). Proportion of available and missing data for each risk factor related measurements are shown here. Note these are available measurements within the time frame of the data extract (see Supplementary Methods). Family history not shown as coded only as yes with unknown negative/missing data. See also Supplementary Table 12 for the completeness values.

Figure 4: Proportion of attendees and non-attendees with common CVD risk factors. Definitions as per Supplementary Table 6 and include: High cholesterol = total cholesterol >5mmol/L or cholesterol ratio >4; High blood pressure = systolic ≥140 or diastolic pressure ≥90mmHg; Obesity = BMI≥30kg/m^2; Alcohol > low risk = AUDIT C score ≥8; Low physical activity = GPPAQ moderate inactive or inactive; Possible Diabetes = HbA1c ≥48mmol/mol or FBG>7mmol/L; Current Smoker = current smoking; High CVD Risk score = 10 year CVD risk score ≥10%. *Family history is predominantly only recorded if present so accurate information on its absence is unavailable. See also Supplementary Table 6 for more detailed information.

For peer review only - http://bmjopen.bmj.com/site/about/guidelines.xhtml

REFERENCES

1. Global Burden of Diseases Causes of Death Collaborators. Global, regional, and national age-sex-specific mortality for 282 causes of death in 195 countries and territories, 1980-2017: a systematic analysis for the Global Burden of Disease Study 2017. *Lancet* 2018;392(10159):1736-88. doi: 10.1016/S0140-6736(18)32203-7
2. Department of Health. Putting Prevention First: Vascular checks, risk assessment and management 2008 [Available from: https://www.healthcheck.nhs.uk/seecmsfile/?id=1302 accessed December 2019.
3. Global Burden of Diseases Risk Factor Collaborators. Global, regional, and national comparative risk assessment of 84 behavioural, environmental and occupational, and metabolic risks or clusters of risks for 195 countries and territories, 1990-2017: a systematic analysis for the Global Burden of Disease Study 2017. *Lancet* 2018;392(10159):1923-94. doi: 10.1016/S0140-6736(18)32225-6
4. Department of Health. Economic Modelling for Vascular Checks 2009 [Available from: www.healthcheck.nhs.uk/document.php?o=225 accessed February 2020.
5. Public Health England. NHS Health Check Best Practice Guidance 2019 [updated October 2019. Available from: https://www.healthcheck.nhs.uk/seecmsfile/?id=1474 accessed February 2020.
6. NHS. NHS Long Term Plan 2018 [updated August 2019. Available from: https://www.england.nhs.uk/long-term-plan/ accessed February 2020.
7. Department for Health and Social Care. Advancing Our Health: Prevention in the 2020's Online2019 [Available from: https://www.gov.uk/government/consultations/advancing-our-health-prevention-in-the-2020s/advancing-our-health-prevention-in-the-2020s-consultation-document accessed March 2020.
8. Department of Health and Social Care. The Local Authorities (Public Health Functions and Entry to Premises by Local Healthwatch Representatives) Regulations London2013 [Available from: http://www.legislation.gov.uk/uksi/2013/351/regulation/4/made accessed December 2019.
9. Capewell S, McCartney M, Holland W. NHS Health Checks--a naked emperor? *J Public Health (Oxf)* 2015;37(2):187-92. doi: 10.1093/pubmed/fdv063
10. Martin A, Saunders CL, Harte E, et al. Delivery and impact of the NHS Health Check in the first 8 years: a systematic review. *Br J Gen Pract* 2018;68(672):e449-e59. doi: 10.3399/bjgp18X697649
11. Robson J, Dostal I, Sheikh A, et al. The NHS Health Check in England: an evaluation of the first 4 years. *BMJ Open* 2016;6(1):e008840. doi: 10.1136/bmjopen-2015-008840
12. Herrett E, Gallagher AM, Bhaskaran K, et al. Data Resource Profile: Clinical Practice Research Datalink (CPRD). *Int J Epidemiol* 2015;44(3):827-36. doi: 10.1093/ije/dyv098
13. NHS Digital. NHS Health Check Programme: Interactive Dashboard 2019 [updated October 2019. Available from: https://digital.nhs.uk/data-and-information/data-tools-and-services/data-services/general-practice-data-hub/nhs-health-check-programme accessed February 2020.
14. NHS Digital. NHS Health Checks business rules NHS Digital 2018 [Available from: https://digital.nhs.uk/services/general-practice-gp-collections/service-information/nhs-health-checks-business-rules accessed February 2020.
15. NHS Digital. Data Extract Advisory Committee to the NHS Health Check data extract 2018 [Available from: https://www.healthcheck.nhs.uk/commissioners-and-providers/governance/data-extract-advisory-committe-deac/ accessed February 2020.
16. Benchimol EI, Smeeth L, Guttmann A, et al. The REporting of studies Conducted using Observational Routinely-collected health Data (RECORD) Statement. *PLOS Medicine* 2015;12(10):e1001885. doi: 10.1371/journal.pmed.1001885

For peer review only - http://bmjopen.bmj.com/site/about/guidelines.xhtml

17. NHS Digital. Privacy Notice: NHS Health Check for adults aged 40-74 years: NHS Digital 2019 [updated February 2019. Available from: https://digital.nhs.uk/services/general-practice-gp-collections/service-information/nhs-health-checks accessed February 2020.
18. NHS Digital. General Practice Extraction Service (GPES) 2019 [updated December 2019. Available from: https://digital.nhs.uk/services/general-practice-extraction-service accessed February 2020.
19. NHS Digital. Direction for the NHS health check for adults aged 40-74 years data extraction 2018 [updated October 2019. Available from: https://digital.nhs.uk/about-nhs-digital/corporate-information-and-documents/directions-and-data-provision-notices/public-health-england-directions/direction-for-the-nhs-health-check-for-adults-aged-40--74-years-data-extraction accessed February 2020.
20. Public Health England. Public Health Outcome Framework: NHS Health Check indicators: Public Health England; 2019 [updated December 2019. Available from: https://fingertips.phe.org.uk/profile/nhs-health-check-detailed accessed February 2020.
21. Usher-Smith. J MA, Harte. E, MacLure. C, Meads. C, Saunders. C, Griffin. S, Walter. F, Lawrence. K, Robertson. C, Mant. J,. NHS Health Check programme rapid evidence synthesis 2017 [Available from: www.healthcheck.nhs.uk/commissioners_and_providers/evidence/ accessed February 2020.
22. Attwood S, Morton K, Sutton S. Exploring equity in uptake of the NHS Health Check and a nested physical activity intervention trial. *J Public Health (Oxf)* 2016;38(3):560-68. doi: 10.1093/pubmed/fdv070
23. Dalton AR, Bottle A, Okoro C, et al. Uptake of the NHS Health Checks programme in a deprived, culturally diverse setting: cross-sectional study. *J Public Health (Oxf)* 2011;33(3):422-9. doi: 10.1093/pubmed/fdr034
24. Cochrane T, Gidlow CJ, Kumar J, et al. Cross-sectional review of the response and treatment uptake from the NHS Health Checks programme in Stoke on Trent. *J Public Health (Oxf)* 2013;35(1):92-8. doi: 10.1093/pubmed/fds088
25. Patel RS, Lagord C, Waterall J, et al. Online self-assessment of cardiovascular risk using the Joint British Societies (JBS3)-derived heart age tool: a descriptive study. *BMJ Open* 2016;6(9):e011511. doi: 10.1136/bmjopen-2016-011511
26. NHS Digital. Health Survey for England 2018 [updated December 2019. Available from: https://digital.nhs.uk/data-and-information/publications/statistical/health-survey-for-england/2018 accessed February 2020.
27. National Institute for Health and Care Excellence. Cardiovascular disease: risk assessment and reduction, including lipid modification 2014 [updated September 2016. Available from: https://www.nice.org.uk/guidance/cg181 accessed February 2020.
28. Public Health England. Health matters: preventing cardiovascular disease 2019 [Available from: https://www.gov.uk/government/publications/health-matters-preventing-cardiovascular-disease/health-matters-preventing-cardiovascular-disease#cvd-ambitions-and-secondary-prevention accessed May 2020.
29. Barron E, Clark R, Hewings R, et al. Progress of the Healthier You: NHS Diabetes Prevention Programme: referrals, uptake and participant characteristics. *Diabet Med* 2018;35(4):513-18. doi: 10.1111/dme.13562

For peer review only - http://bmjopen.bmj.com/site/about/guidelines.xhtml

Table 1- Attendance to an NHS Health Check by financial year among individuals aged 40 - 74 years in England between April 2012 and March 2017 (N=9,694,979)

Financial Year	Individuals offered an NHS health check	Individuals attending an NHS health check	Uptake of offers rate %
2012/2013	1,469,031	742,935	50.6
2013/2014	1,796,483	962,831	53.6
2014/2015	2,162,454	1,135,746	52.5
2015/2016	2,154,129	1,142,151	53.0
2016/2017	2,112,882	1,119,095	53.0
**Total**	**9,694,979**	**5,102,758**	**52.6**

For peer review only

For peer review only - http://bmjopen.bmj.com/site/about/guidelines.xhtml

Table 2: Socio-demographic characteristics of NHSHC invitees April 2012 - March 2017 compared with ONS estimated English population aged 40-74 at mid-2015

Socio-demographic characteristic	ONS mid-2015 England resident population (aged 40-74 years)	NHSHC Invitees (%)	Attendees n (%)	Non-attendees n (%)
**Sex**				
Male	11,200,690 (49.1)	4,724,015 (48.7)	2,311,604 (45.3)	2,412,411 (52.5)
Female	11,604,922 (50.9)	4,970,906 (51.3)	2,791,130 (54.7)	2,179,776 (47.5)
Unknown	-	58 (0.0)	24 (0.0)	34 (0.0)
**Age group (years)**				
40-44	3,636,454 (15.9)	2,208,213 (22.8)	984,908 (19.3)	1,223,305 (26.6)
45-49	3,889,360 (17.1)	1,986,966 (20.5)	966,356 (18.9)	1,020,610 (22.2)
50-54	3,811,000 (16.7)	1,833,267 (18.9)	958,263 (18.8)	875,004 (19.1)
55-59	3,278,322 (14.4)	1,414,091 (14.6)	783,740 (15.4)	630,351 (13.7)
60-64	2,904,721 (12.7)	1,105,914 (11.4)	669,503 (13.1)	436,411 (9.5)
65-69	3,017,135 (13.2)	910,089 (9.4)	585,653 (11.5)	324,436 (7.1)
70-74	2,268,620 (9.9)	236,439 (2.4)	154,335 (3.0)	82,104 (1.8)
**Ethnic Group**				
White	20,383,677 (89.4)	6,946,824 (71.7)	4,067,864 (79.7)	2,878,960 (62.7)
Indian	524,313 (2.3)	202,004 (2.1)	136,598 (2.7)	65,406 (1.4)
Pakistani	291,546 (1.3)	137,222 (1.4)	89,970 (1.8)	47,252 (1)
Bangladeshi	101,926 (0.4)	46,802 (0.5)	34,863 (0.7)	11,939 (0.3)
Black African	314,107 (1.4)	147,462 (1.5)	94,539 (1.9)	52,923 (1.2)
Black Caribbean	271,649 (1.2)	79,987 (0.8)	53,621 (1.1)	26,366 (0.6)
Chinese	121,129 (0.5)	44,730 (0.5)	27,360 (0.5)	17,370 (0.4)
Other Asian	302,667 (1.3)	125,853 (1.3)	79,354 (1.6)	46,499 (1)
Other Group	494,599 (2.2)	239,024 (2.5)	142,621 (2.8)	96,403 (2.1)
Not Stated		104,136 (1.1)	31,319 (0.6)	72,817 (1.6)
Missing		1,620,935 (16.7)	344,649 (6.8)	1,276,286 (27.8)
**Deprivation Index (IMD Decile)**				
Most deprived	1,914,356 (8.4)	853,547 (8.8)	420,547 (8.2)	433,000 (9.4)

For peer review only

2	1,999,183 (8.8)	896,809 (9.3)	472,647 (9.3)	424,162 (9.2)
3	2,083,743 (9.1)	904,131 (9.3)	477,140 (9.4)	426,991 (9.3)
4	2,202,902 (9.7)	921,244 (9.5)	477,516 (9.4)	443,728 (9.7)
5	2,304,663 (10.1)	974,023 (10)	509,715 (10.0)	464,308 (10.1)
6	2,402,719 (10.5)	991,135 (10.2)	517,381 (10.1)	473,754 (10.3)
7	2,443,073 (10.7)	1,044,505 (10.8)	547,909 (10.7)	496,596 (10.8)
8	2,458,761 (10.8)	1,034,751 (10.7)	547,016 (10.7)	487,735 (10.6)
9	2,491,679 (10.9)	1,045,098 (10.8)	565,872 (11.1)	479,226 (10.4)
Least deprived	2,504,533 (11.0)	1,022,539 (10.5)	563,798 (11.0)	458,741 (10.0)
Missing		7,197 (0.1)	3,217 (0.1)	3,980 (0.1)
**Patient characteristics**				
Deaf	n/a	321 (0.0)	171 (0.0)	150 (0.0)
Blind	n/a	13,405 (0.1)	7,224 (0.1)	6,181 (0.1)
Severe Mental Illness	n/a	111,878 (1.2)	59,351 (1.2)	52,527 (1.1)
Learning Disability	n/a	39,612 (0.4)	21,535 (0.4)	18,077 (0.4)
Dementia	n/a	7,521 (0.1)	3,060 (0.1)	4,461 (0.1)
Rheumatoid Arthritis	n/a	74,281 (0.8)	38,104 (0.7)	36,177 (0.8)
Total	22,805,612	9,694,979	5,102,758	4,592,221

ONS= Office for National Statistics, NHSHC = NHS Health Check, IMD = Index of multiple deprivation

For peer review only

For peer review only - http://bmjopen.bmj.com/site/about/guidelines.xhtml

Table 3 Number and proportion of attendees that were coded as received advice, information or a referral following their NHSHC among all attendees and attendees with CVD risk factors

**Intervention type**	**All Attendees n (%)**	**Attendees with the CVD risk factor above threshold for intervention n (%)**
Alcohol Consumption	792,761 (15.5)	46,611 (38.4)
Diet	1,189,986 (23.3)	766,521 (25.1)
Physical Activity	1,501,103 (29.4)	434,326 (39.3)
General Lifestyle/ Behaviours	814,611 (16.0)	211,571 (20.1)
Smoking Cessation	865,913 (17)	467,119 (57.3)
Weight Loss and Obesity	821,414 (16.1)	599,380 (19.6)
Diabetes Prevention Programme (DPP)	4,551 (0.1)	3,348 (0.9)
Total	*2,501,565 (49.0)*	*565,047 (53.7)*

Thresholds defined in Supplementary Table 8, DPP = diabetes prevention programme

For peer review only

For peer review only - http://bmjopen.bmj.com/site/about/guidelines.xhtml

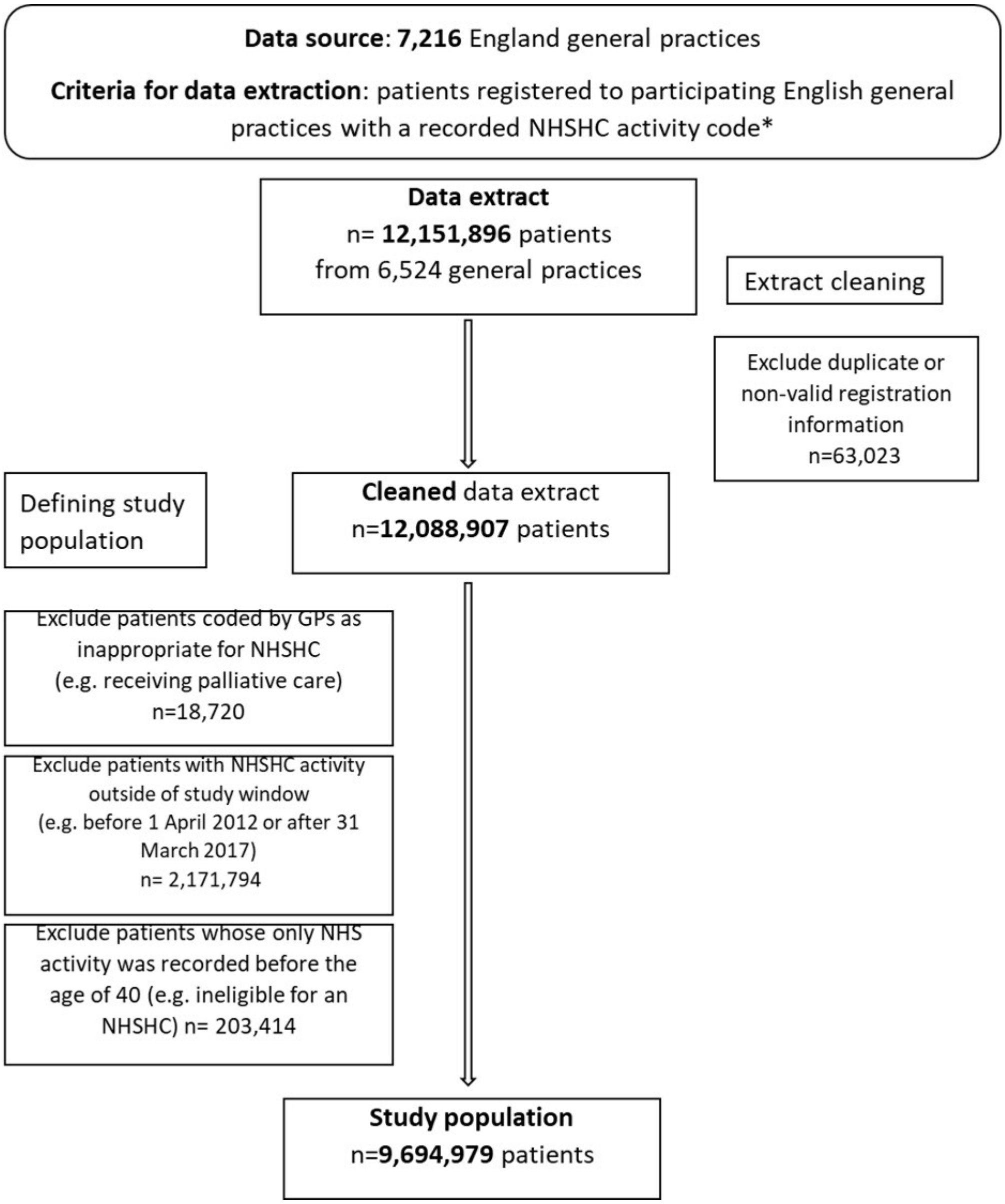

Figure 1: Study extract and study population flow chart

159x190mm (149 x 149 DPI)

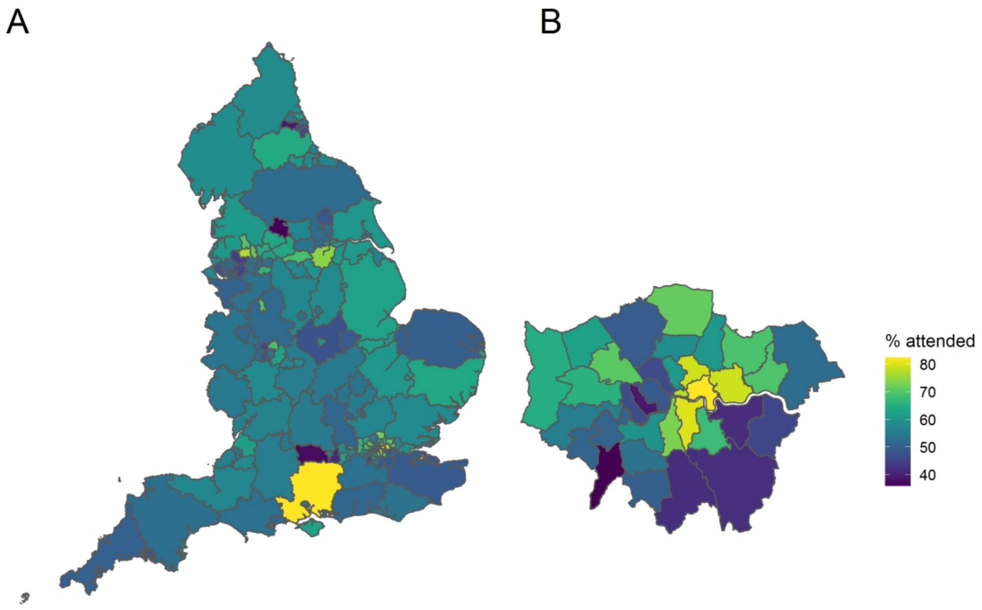

Figure 2: Variation in NHSHC uptake across (A) England and (B) London

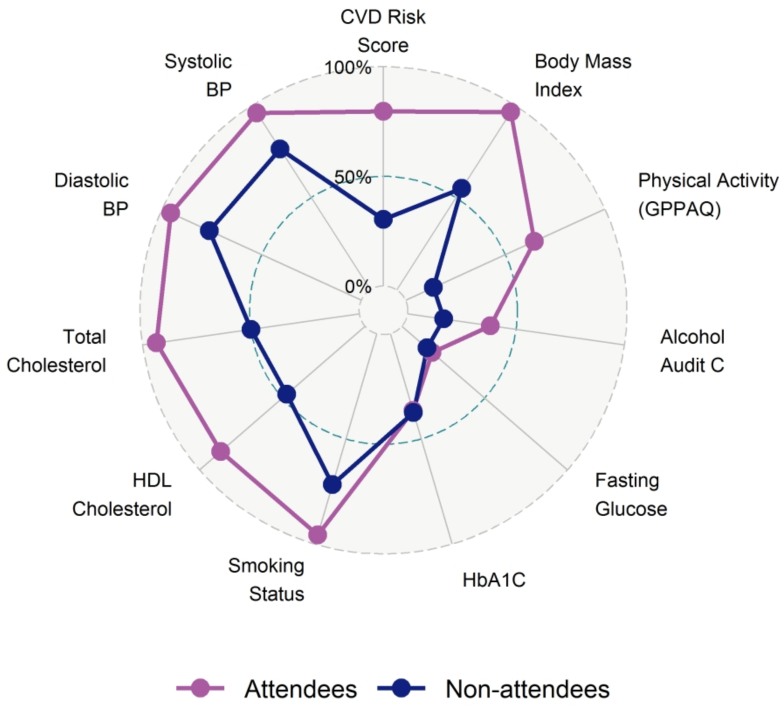

Figure 3: Completion of risk factor measurements for attendees and non-attendees (2012/13 - 2016/17)

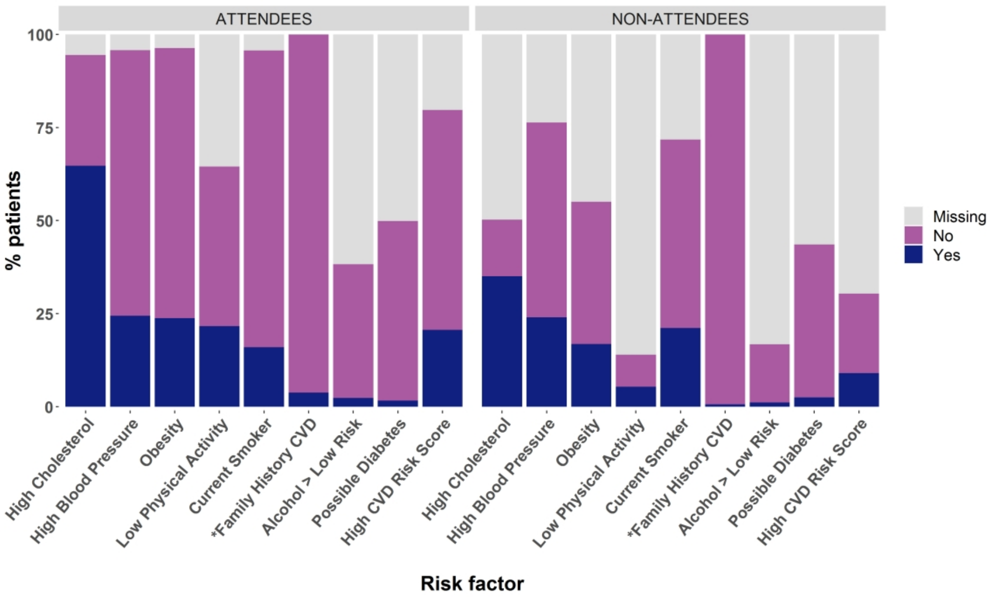

Figure 4: Proportion of attendees and non-attendees with common CVD risk factors

Supplementary Materials

An evaluation of the uptake and delivery of the NHS Health Check Programme in England, using primary care data from 9.5 million people: A cross-sectional study

Contents

Supplementary Methods 2

Supplementary Figures 4

Supplementary Figure 1 - Invitation type for first invitation record by year of invitation among attendees and non-attendees 4

Supplementary Tables 5

Supplementary Table 1: Read codes for NHS Health Check activity codes and prioritisation rules for definition of primary contact with programme 5

Supplementary Table 2: Data extraction rules 6

Supplementary Table 3: Plausible ranges for risk factor measurements 7

Supplementary Table 4: Order of priority for selecting metrics in time window around patient's index date 8

Supplementary Table 5: Derived Ethnic Group Categories 16

Supplementary Table 6: Categories for risk factors - Risk factors by binary cut points 17

Supplementary Table 7: Rules for conflicting risk factors measurements 18

Supplementary Table 8: Intervention risk thresholds for action 18

Supplementary Table 9: Data for attendance by UTLA 19

Supplementary Table 10: Number of invitations recorded for attendees and non-attendees 22

Supplementary Table 11: Invitations by financial year 23

Supplementary Table 12: Completeness of risk factor measurement 23

Supplementary Table 13: Statin prescription rates 24

For peer review only - http://bmjopen.bmj.com/site/about/guidelines.xhtml

Supplementary Methods

Data Management and Cleaning

The data extract was stored within a Structured Query Language (SQL) database and processed using queries within SQL Server Management Studio. Duplicate patient records were removed. Implausible values were re-coded as missing values. Plausible ranges for risk factors, Supplementary Table 3, were defined by DEAC.

Definitions and Study Variables

Individuals were categorised as either NHSHC attendees if they had a Read code for a completed check within the 5-year period, or a non-attendee if they did not. Further details are provided in Supplementary Table 1. Uptake of the programme was defined as the proportion of the total study population who attended.

An index date was generated from the date of an individual's primary NHSHC activity to identify age and the most relevant risk factor measurements for each patient. Risk factor and clinical measurements were selected for analysis if they occurred on the index date, otherwise we took the closest recording within pre-defined time windows set by the DEAC. A full list of variables, Read codes used to define variables, time windows and coding algorithms is available in Supplementary Table 4.

An individual's age in years was estimated based on year of birth and index date and presented in five-year intervals. We derived an ethnic group variable with the aim of generating fewer categories while still representing important ethnic groups for CVD (Supplementary Table 5). We also included Index of Multiple Deprivation (IMD) (2015) national deciles matched at Lower Super Output Area (LSOA) level based on the patient's postcode of residence at the time of data extraction.[1] ONS April 2019 upper tier local authority (UTLA) boundaries were used.[2] Gender was reported as coded in the extract (Male; Female). Learning difficulty, serious mental illness (SMI), blindness, deafness, rheumatoid arthritis and dementia (present/absent) are reported as binary variables.

We present the following risk factors as binary variables, using cut-points defined in consultation with DEAC, Supplementary Table 6; obesity (BMI>30kg/m^2), blood pressure (derived from systolic (>=140mmHg) or diastolic blood pressure (>=90mmHg), cholesterol (total cholesterol >5mmol/L or cholesterol ratio >4), blood glucose (fasting plasma glucose >=7mmol/L or HbA1C>=48mmol/mol), smoking (current), physical activity (general practice physical activity questionnaire = moderately

For peer review only - http://bmjopen.bmj.com/site/about/guidelines.xhtml

inactive or inactive), alcohol intake and behaviour (Audit C score >=8), CVD risk score (10 year risk >=10%) and family history of CVD before 60 years. Rules for conflicting measures for the same patient on the same day are available in Supplementary Table 7.

Among attendees, we considered invitations in the 365 days prior to the index date. Time to attendance was derived from the number of days between first recorded invitation and the index date. Invitation type for attendees was grouped into three categories: advanced invitation (invitation recorded prior to date of NHSHC), opportunistic invitation (invitation recorded same date as NHSHC) and missing invitation (invitation not recorded but NHSHC completed). Among non-attendees for whom the primary contact was an invitation, we considered invitations in the 365 days after the index date. The provider delivering the NHSHC (GP staff; third party) was reported as a binary variable.

Among attendees, we present data for delivery of advice, information or referral for diet, alcohol, physical activity, smoking, weight loss and general lifestyle, referrals for diabetes prevention and prescriptions for statins (present/absent) as binary variables. Statin prescribing data was made available by three out of four GP clinical IT system providers, and subsequently a Read code was attached to 60.4% of attendees in the dataset. We present data for any statin prescription on or after the date of NHSHC activity, as individuals with current statin prescriptions would not be eligible for an invitation to the NHSHC. We also present these data among attendees with a risk profile indicating that intervention was appropriate. We defined appropriate thresholds for action of intervention through consultation with the DEAC advisory board. These are available in Supplementary Table 8.

REFERENCES

1. Office for National Statistics. English indices of deprivation 2015 2015 [Available from: https://www.gov.uk/government/statistics/english-indices-of-deprivation-2015.
2. Office for National Statistics. Counties and Unitary Authorities (April 2019) Boundaries EW BFC 2019 [updated November 2019. Available from: https://geoportal.statistics.gov.uk/datasets/counties-and-unitary-authorities-april-2019-boundaries-ew-bfc accessed December 2019.

For peer review only - http://bmjopen.bmj.com/site/about/guidelines.xhtml

Supplementary Figures

Supplementary Figure 1 - Invitation type for first invitation record by year of invitation among attendees and non-attendees

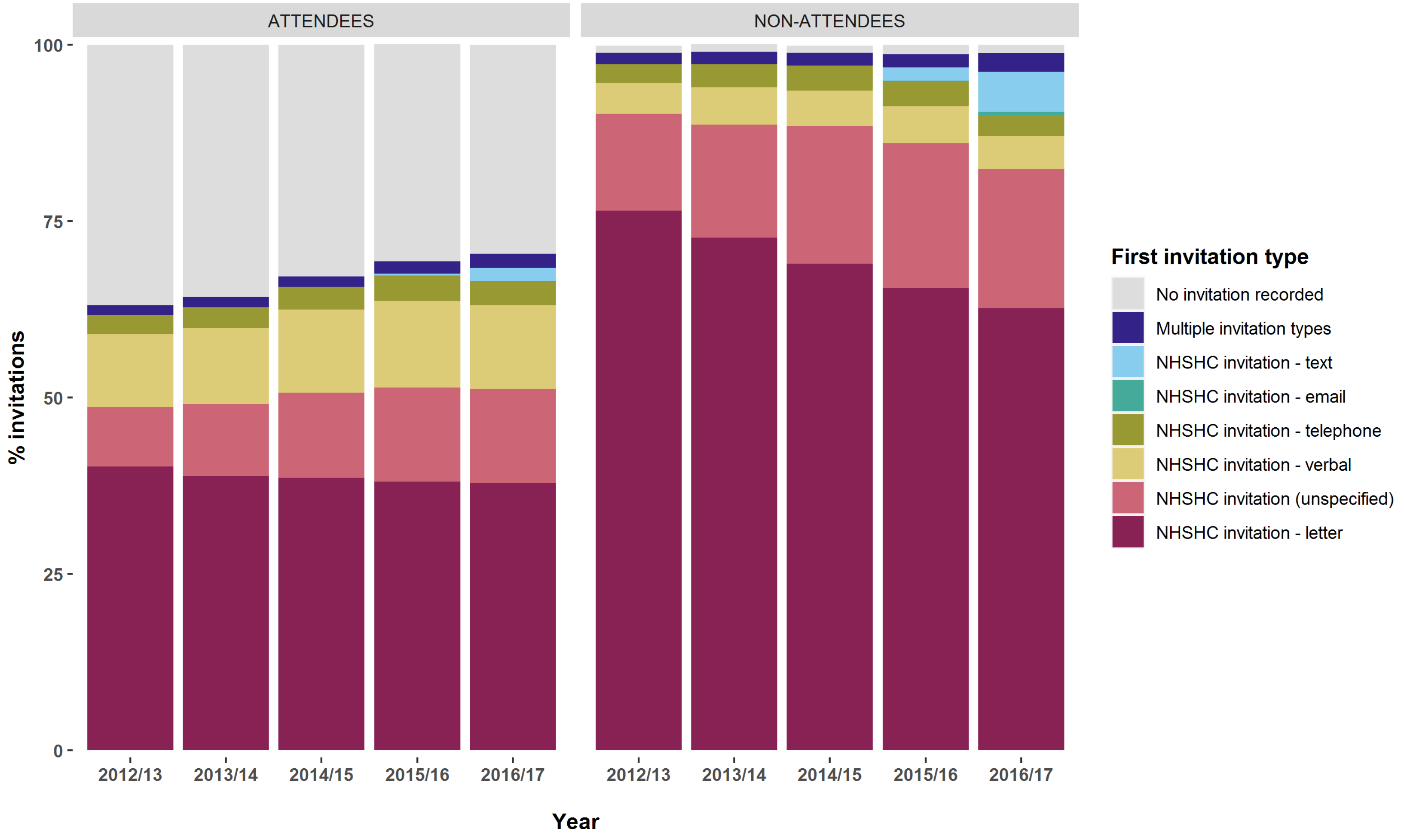

For peer review only - http://bmjopen.bmj.com/site/about/guidelines.xhtml

Supplementary Tables

Supplementary Table 1: Read codes for NHS Health Check activity codes and prioritisation rules for definition of primary contact with programme

Order	Clinical NHSHC activity code	Read V2 clinical codes (date introduced)	CTV3 clinical codes (date introduced)	Reported grouping	Criteria
1	Inappropriate	9NSH. (01/10/2013)	Xaaac (01/10/2013)	Excluded from study	Patient has a code recorded as being inappropriate for an NHS Health Check in the data extract
2	Completed	8BAg. (01/04/2010)<br><br>8BAg0 (01/10/2012)	XaRBQ (01/04/2010)<br><br>XaZPq (01/10/2012)	Attendee	Patient has a completed NHS Health Check code recorded in the 5-year period<br><br>**Index date**: date of patient's first completed check code
3	Declined	8IAx. (01/04/2011)	XaX8h (01/04/2011)	Non-attendee	Patient has a declined NHS Health Check code recorded in the 5-year period<br><br>**Index date:** date of patient's first declined code
4	Did not attend	9NiS. (01/04/2010)	XaRAA (01/04/2010)	Non-attendee	Patient has an NHS Health Check not attended code recorded in the 5-year period<br><br>**Index date:** date of patient's first non-attendance code
5	Commenced	8CV9. (01/04/2016)	Xaeab (01/04/2016)	Non-attendee	Patient has a commenced NHS Health Check code recorded in the 5-year period (and no completed/did not attend/declined code recorded in the following 8 weeks)<br><br>**Index date:** date of patient's first commenced code
6	Invitation	9mC.., 9mC0., 9mC1., 9mC2., 9mC3., 9mC4., (01/04/2010)<br><br>9mC5., 9mC6. (01/10/2015)	XaRBR, XaR9z, XaRBS, XaRBT, XaRBU, XaRBV (01/04/2010)<br><br>Xad0C, Xad0D, (01/10/2015)	Non-attendee	Patient has an invitation to attend an NHS Health Check code recorded in the 5-year period (and no follow up (non-invitation) code recorded within the following 6 months)<br><br>**Index date:** date of patient's first invitation code

For peer review only - http://bmjopen.bmj.com/site/about/guidelines.xhtml

Supplementary Table 2: Data extraction rules

Event of interest (NHS Health check offer, completed, DNA, declined …)

GPES EXTRACT

Category data item	Details data item	before event	after event
Patients demographics & equity measures	Carer status	latest code before event	1 month
	Learning disabilities, serious mental illness, blindness, deafness, dementia	include only first recorded code (if not 'resolved')	
	Deprivation (calculated), age (calculated), gender, ehtnicity, first language	latest available code	
CVD risk factor	Smoking status, height, weight, BMI, waist circum., alcohol use, physical activity, CVD risk score	all codes 2 years before event	all codes after event
Tests results	Blood pressure, pulse, cholesterol & HDL, glucose/ HbA1c, eGFR, ACR	all codes 2 years before event	all codes after event
CVD diagnosis (**)	Family history CVD, CHD, CKD, diabetes type 2, TIA, stroke, AF, Heart failure, hypertension, familial hypercholesterolemia, hypercholesterolemia, hyperlipidaemia, PAD. (+ non-diabetic hyperglycaemia, impaired glucose regulation, impaired fasting glycaemia, impaired glucose tolerance) (+ rheumatoid arthritis)	all codes (if not resolved)	
Referrals	Referral to NHS DPP (start 2016)	all codes	
	Advice, signposting, referral to lifestyle services, referral for other CVD assessment (diabetes, hypertension, …)	latest code before event	all codes within 1 year of event
Prescribed CVD medication	Lipid regulation, anti-hypertensive, anti-coagulant, diabetes treatment, AF treatment	all codes 2 years before event	all codes within 1 year of event

Supplementary Table 3: Plausible ranges for risk factor measurements

Risk factor	Plausible measurement range (inclusive unless stated)
Alcohol risk score (AUDIT; AUDITC; FAST)	0 – 40
Blood pressure - systolic	70 – 300 mmHg
Blood pressure - diastolic	20 – 150 mmHg
BMI	12 – 90 kg/m^2
Cholesterol – total	1 – 40 (exclusive)
Cholesterol – HDL	0.5 – 5
Cholesterol – ratio	0.2 – 80
Fasting Plasma Glucose (FPG)	0 (exclusive) – 100
HbA1c	20 – 195 mmol/mol
Height	100 – 230 cm
CVD risk score	0 – 100
Weight	20 – 250 kg

For peer review only

Supplementary Table 4: Order of priority for selecting metrics in time window around patient's index date

Metric	First priority	Second priority	Third priority	Derivation / other prioritisation rules	Clinical codes (Read V2)	Clinical codes (CTV3)
**Patient characteristics**						
Ethnic group	Ethnic group recorded in patient's GPES profile at time of data extraction (31/3/2018)	Most recent ethnic group recorded via a clinical code (looking over whole data extract)	n/a	n/a	9S...% , 9T...% , 9t...% , 9i...%	XaBEN%
Blindness	On index date	Anytime before index date (most proximal to index date used)	n/a	n/a	6689. , 6688. , 668D. , 668C.	6689.% , XaW0l , XaCGX% , XaLMz
Deafness	On index date	Anytime before index date (most proximal to index date used)	n/a	n/a	F599. , F591B , F591E , F59A. , F5919	XaRE4 , XaZuB , XaZuE , XaaLf , XaRE5 , Xa0PN
Dementia	On index date	Anytime before index date (most proximal to index date used)	n/a	n/a	Eu02.% , E00..% , Eu01.% , E02y1 , E012.% , Eu00.% , E041. , Eu041 , F110.- F112. , F116. , F118. , F21y2 , A410. , A411.%	X002w% (excluding X003E , X003F , X001T) , Eu02.% , XE1Xt , E00z. , E02y1
Learning Disability	On index date	Anytime before index date (most proximal to index date used)	n/a	n/a	E3...% , Eu7..% , Eu814 , Eu815 , Eu816 , Eu817 , Eu81z , 918e. , Eu818	E3...% , XaQZ4 , XaQZ3 , XaKYb , XaREt , XaREu , Eu81z , XaaiS , Xabk1
Severe Mental Illness	On index date	Anytime before index date (most proximal to index date used)	n/a	n/a	E10..% , E110.% , E111.% , E1124 , E1134 , E114.- E117z , E11y.% (excluding E11y2) , E11z. , E11z0 , E11zz , E12..% , E13..% (excluding E135.) , E2122 , Eu2..% , Eu30.%	X00S6% (excluding Xa9B0% , E14..%) , X00SL , X00SM% , X00SJ% , XSGon , E11z. , E11z0 , E11zz , XE1ZZ , XE1Ze , XaX54 , XaX53 , E130. , E1124 , E1134

For peer review only - http://bmjopen.bmj.com/site/about/guidelines.xhtml

					, Eu31.% , Eu323 , Eu328 , Eu333 , Eu32A , Eu329	
**CVD risk factors**						
Family history of CVD	On index date	Anytime before index date (most proximal to index date used)	Anytime after index date (most proximal to index date used)	n/a	12C.. , 12C2. , 12C3. , 12C4. , 12C5. , 12CA. , 12CB. , 12CC. , 12CD. , 12CE. , 12CF. , 12CG. , 12CH. , 12CI. , 12CL. , 12CM. , 12CN. , 12CP. , 12CV. , 12CW. , 12CZ.	XaP9K , XaP9M , ZV174 , XE24Z , XaLQq , Xa6aj% , XM1Jg , XM1Jw% , XaP9K , XaP9M
Rheumatoid arthritis	On index date	Anytime before index date (most proximal to index date used)	**Attendees:** n/a<br><br>**Non-attendees:** Anytime after index date (most proximal to index date used)	n/a	N040.% , N041. , N042.% (excluding N0420) , N047. , N04X. , N04y0 , N04y2 , Nyu11 , Nyu12 , Nyu1G , Nyu10 , G5yA. , G5y8.	N040.% , XE1DU , X705I , G5y8.
Alcohol AUDIT/AUDIT-C/FAST	On index date	Most proximal score to index date for each of AUDIT, AUDIT-C and FAST used.<br><br>**Attendees:** Up to 365 days before index date<br><br>**Non-attendees:** Anytime before index date	Most proximal score to index date for each of AUDIT, AUDIT-C and FAST used.<br><br>**Attendees:** Up to 90 days after index date<br><br>**Non-attendees:** Anytime after index date	No AUDIT-C/FAST/AUDIT score available: risk factor is **missing**<br><br>AUDIT-C or FAST assessment is positive, but no AUDIT score available: risk factor is **missing**<br><br>AUDIT-C (and/or) FAST assessment is negative: risk factor is **low risk**<br><br>AUDIT score available and greater than or equal to 8: risk factor is **high risk**	38D4. (AUDIT-C), 388u. (FAST), 38D3. (AUDIT)	XaORP (AUDIT-C), XaNO9 (FAST), XM0aD (AUDIT)

For peer review only - http://bmjopen.bmj.com/site/about/guidelines.xhtml

Blood pressure	On index date	Systolic and diastolic BP recordings recorded most proximal to index date used.<br><br>**Attendees:** Up to 365 days before index date<br><br>**Non-attendees:** Anytime before index date	Systolic and diastolic BP recordings recorded most proximal to index date used.<br><br>**Attendees:** Up to 90 days after index date<br><br>**Non-attendees:** Anytime after index date	On examination (O/E) readings considered only.<br><br>Systolic BP or Diastolic BP is unavailable: risk factor is **missing**	246..% (excluding 2460. , 2468. , 246H. , 246I. , 246K. , 246L. , 246M. , 246h. , 246i. , 246j. , 246k. , 246n.% , 246o.%)	X773t% (excluding XaI9f , XaI9g , XaZvo , XaZxj , X779b , X779R , X779T , X779W , XaYai , XaYg8 , XaYg9 , Xabhx , Xac5K , Xac5L , Xaedn%) , 246..% (excluding 2460. , 2468. , XaCFN , XaCFO)
Blood glucose	On index date	HbA1c and Fasting Plasma Glucose recorded most proximal to index date considered.<br><br>**Attendees:** Up to 365 days before index date<br><br>**Non-attendees:** Anytime before index date	HbA1c and Fasting Plasma Glucose recorded most proximal to index date considered.<br><br>**Attendees:** Up to 90 days after index date<br><br>**Non-attendees:** Anytime after index date		HbA1c:<br>42W5. , 42W50 , 42W51<br><br>Fasting Plasma Glucose:<br>44g1.	HbA1c:<br>XaPbt , Xaezd , Xaeze<br><br>Fasting Plasma Glucose:<br>44g1.
Body mass index	On index date	Most proximal to index date used.<br><br>**Attendees:** Up to 365 days before index date<br><br>**Non-attendees:** Anytime before index date	Most proximal to index date used.<br><br>**Attendees:** Up to 90 days after index date<br><br>**Non-attendees:** Anytime after index date	If BMI is unavailable but height and weight are, BMI is calculated (BMI = kg/m^2)<br><br>Height and weight are not used if BMI is available	BMI:<br>22K..% (excluding 22K9.% , 22KA.)<br><br>Weight:<br>22A..% (excluding 22A7.-22A9.) , 9NSa. , 8IAH.<br><br>Height:<br>229..% (excluding 2296.) , 9NSZ. , 8IHM.	BMI:<br>22K..% (excluding XaVwA% , X76CN , XaZMj) , Xa7wG%<br><br>Weight:<br>22A..% , 22AA. , X76C3 , XaesG , XaQ7T<br><br>Height:

For peer review only - http://bmjopen.bmj.com/site/about/guidelines.xhtml

						229..% (excluding 2296.) , XaesF , Xaef4
Cholesterol (ratio)	On index date	Most proximal to index date used.<br><br>**Attendees:** Up to 365 days before index date<br><br>**Non-attendees:** Anytime before index date	Most proximal to index date used.<br><br>**Attendees:** Up to 90 days after index date<br><br>**Non-attendees:** Anytime after index date	If cholesterol ratio is unavailable but total and HDL cholesterol are, the cholesterol ratio is calculated (ratio = total/HDL)<br><br>Total and HDL cholesterol are not used if cholesterol ratio is available	Cholesterol:<br>44O5. , 44PH. , 44P5. , 44PF. , 44PJ. , 44P.. , 44OE. , 44P1. , 44P2. , 44P3. , 44P4. , 44PK. , 44PZ. , 44l2. , 44lF. , 44lG. , 662a.<br><br>HDL cholesterol:<br>44P5. , 44PB. , 44PC. , 44d3. , 44d2.	Cholesterol:<br>XaFs9 , XSK14 , 44P5. , 44PF , 44PJ. , XalRd , XE2eD% , 44P1. , 44P2. , 44P3. , 44P4. , 44PH. , XaERR , XaEUq , XaEUr , X772L<br><br>HDL cholesterol:<br>X772M , 44P5. , 44PB. , 44PC. , XaEVr , 44d3. , 44d2.
Physical activity (GPPAQ)	On index date	Most proximal to index date used.<br><br>**Attendees:** Up to 365 days before index date<br><br>**Non-attendees:** Anytime before index date	Most proximal to index date used.<br><br>**Attendees:** Up to 90 days after index date<br><br>**Non-attendees:** Anytime after index date	n/a	138b. , 138a. , 138Y. , 138X. , 38Dh.	XaPPE , XaPPD , XaPPB , XaPP8 , XaXX5
CVD risk score	On index date	QRISK/QRISK2 and Framingham risk score recorded most proximal to index date used.<br><br>**Attendees:** Up to 365 days before index date	QRISK/QRISK2 and Framingham risk score recorded most proximal to index date used.<br><br>**Attendees:** Up to 90 days after index date	QRISK or QRISK2 score recorded most proximal to index date is used if available.<br><br>If QRISK and QRISK2 unavailable, Framingham score is used.	QRISK/QRISK2:<br>8IEL., 8IEV., 38DF., 38DP.<br><br>Framingham:<br>38DR.	QRISK/QRISK2:<br>XaYzy, XaZdA, XaPBq, XaQVY<br><br>Framingham:<br>XaQaG

For peer review only

For peer review only - http://bmjopen.bmj.com/site/about/guidelines.xhtml

		**Non-attendees:** Anytime before index date	**Non-attendees:** Anytime after index date			
Smoking status	On index date	Most proximal to index date used.<br><br>**Attendees:** Up to 365 days before index date<br><br>**Non-attendees:** Anytime before index date	Most proximal to index date used.<br><br>**Attendees:** Up to 90 days after index date<br><br>**Non-attendees:** Anytime after index date	Lookup used to map smoking status to binary categories: Non-smoker; Current smoker	Non-smoker: 1371, 137A., 137l., 137N., 137O., 137S.,<br><br>Current smoker: 137.., 137C., 137e., 137h., 137m., 137P., 137Q., 137R., 137V., 137X., 137Y.,	Non-smoker: 1371, 1377, 1378, 1379, 137B., 137F., 137K., 137T., Ub0p1, Ub1na, Xa1bv, XaQ8V, XE0oj, XE0ok, XE0ol, XE0om, XE0on, XE0op, XE0oh<br><br>Current smoker: 1372, 1373, 1374, 1375, 1376, 137D., 137G., 137J., 137Z., Ub1tI, Ub1tJ, Ub1tK, Ub1tR, Ub1tS, Ub1tU, Ub1tW, XaIIu, XaIkW, XaIkX, XaIkY, XaItg, XaJX2, XaLQh, XaWNE, XaZIE, XE0oq, XE0or
**Interventions – attendees only**						
Advice, informatio n, referral – ALCOHOL	On index date	Up to 365 days after index date	n/a	n/a	Advice, information and any brief intervention given on alcohol usage: 67H0. , 67A5. , 8CAM. , 8CAM0 , 8CAv. , 8CE1. , 9k1A. , 8IAF. , 8IAt. , 9k11. , 9k14. , ZV6D6 , 6792. , 8CdK.<br><br>Referral regarding alcohol usage:	Advice, information and any brief intervention given on alcohol usage: XaJlr , Xa1dA , 67A5. , XaFvp , XaXan , XaPmB , 8CE1. , XaPPv , XaPty , XaX4S , XaKAC , XaKAo , ZV6D6 , 6792. , Xac6H<br><br>Referral regarding alcohol usage:

For peer review only - http://bmjopen.bmj.com/site/about/guidelines.xhtml

					8HkG. , 8H7p. , 8HHe.	XaYWV , XaIPn , XaKUg , XaPna , XaORR
Advice, informatio n, referral – DIET	On index date	Up to 365 days after index date	n/a	n/a	Advice, signposting or information on diet: 67H7. , 8CA4. , 8CA40 , 6799.<br><br>Referral regarding diet: 8H76. , 8H760 , 8HHE.	Advice, signposting or information on diet: XaQaU , 8CA4. , XaXTD , Xa2jQ , XE0i1 , Xa2hD , 6799.<br><br>Referral regarding diet: XaBSz , XaAhZ , XaAha , XaJSp , XaAdX , XaAdY , XaAdZ
Advice, informatio n, referral – LIFESTYLE	On index date	Up to 365 days after index date	n/a	n/a	67H..% , 8Hlu.	XaEFY% , Xaam2
Advice, informatio n, referral – PHYSICAL ACTIVITY	On index date	Up to 365 days after index date	n/a	n/a	Advice, signposting or information on physical activity: 67H2. , 8CA5. , 9Oq3. , 6798. , 8CA52 , 8Cd4. , 8IAv. , 8HBN.<br><br>Referral regarding physical activity: 8H7q. , 8H7q0 , 8HHc. , 8HkX. , 8BAH.	Advice, signposting or information on physical activity: XaJIt , Xa1dN , 8CA5. , XM18T , XaPjx , 6798. , XabFV , XaREx , XaX5H , XaREy<br><br>Referral regarding physical activity: XaIPu , XaR5C , XaKRq , XaREh , XaCmH
Advice, informatio n, referral	On index date	Up to 365 days after index date	n/a	n/a	Support and refer Stop Smoking Service/Advisor:	Support and refer Stop Smoking Service/Advisor:

For peer review only - http://bmjopen.bmj.com/site/about/guidelines.xhtml

– SMOKING					8CAL. , 8HTK. , 8HkQ. , 8H7i. , 8IAj. , 8IEK. , 9N2k. , 13p50 , 9Ndf. , 9Ndg. , 8T08. , 8IEo.<br><br>Advice, signposting or information on smoking: 67H1. , 8CAL. , 67A3. , 8CAg. , 6791. , 8IAj. , 8CdB.	Ua1Nz , XaFw9 , XaQT5 , XaItC , XaIye , XaW0h , XaX5W , XaX5X , XaRFh , XaREz , XaaDy , XaaDx<br><br>Advice, signposting or information on smoking: XaJIs , Ua1Nz , 67A3. , Ua1O0 , XaLD4 , 6791. , XaRFh , XaXnG
Advice, information, referral – WEIGHT	On index date	Up to 365 days after index date	n/a	n/a	Advice, signposting or information on weight management: 67I9. , 8CA40 , 8Cd7. , 66CQ. , 679P. , 8CdC. , 8IAu.<br><br>Referral regarding weight management: 8HHH. , 8HHH1 , 8HHH0 , 8H4n.	Advice, signposting or information on weight management: XaADJ , Xa1dF , XaX5F , XaX5k , XaKHd , XaXnI , XaX5G<br><br>Referral regarding weight management: XaJSu , XaZKe , XaXZ9 , XaZKi
Diabetes Prevention Programme referral	On index date	Up to 365 days after index date	n/a	n/a	679m4, 679m0, 679m1, 679m2	XaeDH, XaeCw, XaeCz, XaeD0
Statin prescriptions	On index date	Up to 365 days after index date	n/a	n/a	bxi..% , bxg..% , bxe..% , bxk..% , bxd..%<br><br>DM+D codes (EMIS): 134489001, 319996000, 319997009, 320000009,	bxi..% , x01R2% , x01R3% , bxk..% , bxd..%

For peer review only - http://bmjopen.bmj.com/site/about/guidelines.xhtml

					320006003, 320012008, 320013003, 320014009, 320029006, 320030001, 320031002, 408036003, 408037007, 409108001, 4896711000001108	

For peer review only

Supplementary Table 5: Derived Ethnic Group Categories

Ethnic group	Subgroups (with ONS codes)
White	A = White British
	B = Irish
	C = Any other White background
	T = White: Gypsy or Irish Traveller
Indian	H = Indian
Pakistani	J = Pakistani
Bangladeshi	K = Bangladeshi
Black African	N = African
Black Caribbean	M = Caribbean
Chinese	R = Chinese
Other Asian	L = Any other Asian background
Other Ethnic Group	D = White and Black Caribbean
	E = White and Black African
	F = White and Asian
	G = Any other mixed background
	P = Any other Black background
	S = Any other ethnic group
	W = Other ethnic group: Arab
Unknown	X = Unknown/No information
	Z = Not stated

For peer review only

For peer review only - http://bmjopen.bmj.com/site/about/guidelines.xhtml

Supplementary Table 6: Categories for risk factors - Risk factors by binary cut points

Risk factors by binary risk cut-offs

Risk factor	High risk threshold/ cutpoint	Risk category	Attendees n (%)	Non-attendees n(%)	Total
Alcohol > Low Risk	Full AUDIT score 8 or more	Missing	3,150,667 (61.7)	3,823,634 (83.3)	6,974,301
		Low risk	1,830,799 (35.9)	714,947 (15.6)	2,545,746
		High risk	121,292 (2.4)	53,640 (1.2)	174,932
Possible Diabetes	HbA1C ≥ 48 or FPG ≥ 7	Missing	2,558,719 (50.1)	2,590,405 (56.4)	5,149,124
		Low risk	2,460,489 (48.2)	1,885,332 (41.1)	4,345,821
		High risk	83,550 (1.6)	116,484 (2.5)	200,034
High Blood Pressure	Systolic BP ≥ 140 or Diastolic BP ≥ 90	Missing	217,714 (4.3)	1,086,797 (23.7)	1,304,511
		Low risk	3,636,511 (71.3)	2,404,097 (52.4)	6,040,608
		High risk	1,248,533 (24.5)	1,101,327 (24)	2,349,860
Obesity	BMI ≥ 30	Missing	187,402 (3.7)	2,064,936 (45)	2,252,338
		Low risk	3,700,522 (72.5)	1,755,019 (38.2)	5,455,541
		High risk	1,214,834 (23.8)	772,266 (16.8)	1,987,100
High Cholesterol	Total cholesterol >5mmol/L or Ratio > 4	Missing	282,100 (5.5)	2,286,595 (49.8)	2,568,695
		Low risk	1,519,485 (29.8)	696,458 (15.2)	2,215,943
		High risk	3,301,173 (64.7)	1,609,168 (35.0)	4,910,341
CVD risk score	10 or more	Missing	1,036,820 (20.3)	3,197,683 (69.6)	4,234,503
		Low risk	3,014,556 (59.1)	979,685 (21.3)	3,994,241
		High risk	1,051,382 (20.6)	414,853 (9)	1,466,235
Family history of CVD	Clinical code present for a CVD event before 60 years old in a first degree relative	No	4,910,543 (96.2)	4,561,766 (99.3)	9,472,309
		Yes	192,215 (3.8)	30,455 (0.7)	222,670
Physical Activity	GPPAQ "moderately inactive" or "inactive"	Missing	1,812,161 (35.5)	3,952,015 (86.1)	5,764,176
		Low risk	2,184,515 (42.8)	392,263 (8.5)	2,576,778
		High risk	1,106,082 (21.7)	247,943 (5.4)	1,354,025
Smoking	Current smoker	Missing	221,351 (4.3)	1,296,474 (28.2)	1,517,825
		Low risk	4,066,412 (79.7)	2,325,196 (50.6)	6,391,608
		High risk	814,995 (16)	970,551 (21.1)	1,785,546

For peer review only - http://bmjopen.bmj.com/site/about/guidelines.xhtml

Supplementary Table 7: Rules for conflicting risk factors measurements

Rules for processing conflicting risk factor measurements for the same patient on the same day

Risk factor	Rule applied
Smoking status; Physical activity status (from GPPAQ)	Records deleted if descriptive statuses are conflicting (e.g. “smoker” and “non-smoker” recorded on the same day)
Blood pressure	Record with lowest systolic measurement taken
BMI; height; weight; QRISK/QRISK2 score; Framingham score; total cholesterol; HDL cholesterol; Cholesterol ratio; HbA1c; FPG	Measurements recoded as missing (unclear which is correct)

Supplementary Table 8: Intervention risk thresholds for action

Intervention type	Advice or Information given	High risk threshold for action
**Advice, information or referral**	Alcohol usage	Alcohol: FULL AUDIT 8 or more
	Diet	Overweight (BMI ≥ 25)
	Physical activity	GPPAQ “moderately inactive” or “inactive”
	Lifestyle/Counselling	CVD risk score 10 or more
	Smoking cessation	Current smoker
	Weight management	Overweight (BMI ≥ 25)
**Diabetes referral**	Diabetes Prevention Programme (DPP) referral	Blood glucose: RAISED risk HbA1C ≥ 42 and < 48 or FPG ≥ 5.5 and < 7
**Statin prescription**	Statins prescribed	CVD risk score 10 or more

For peer review only - http://bmjopen.bmj.com/site/about/guidelines.xhtml

Supplementary Table 9: Data for attendance by UTLA

Number of NHS Health Check invitees and attendees with attendance rate by Upper Tier Local Authority of patient’s residence

UTLA Code	UTLA	Invitees	Attendees	Attendance rate	Lower 95% CI	Upper 95% CI
E10000014	Hampshire	179,937	152,318	84.7	84.5	84.8
E09000030	Tower Hamlets	42,098	34,660	82.3	82.0	82.7
E09000028	Southwark	41,938	33,536	80.0	79.6	80.3
E09000025	Newham	51,556	40,706	79.0	78.6	79.3
E09000012	Hackney	37,636	29,713	78.9	78.5	79.4
E08000001	Bolton	64,013	49,792	77.8	77.5	78.1
E09000001	City of London	1,176	910	77.4	74.9	79.7
E08000017	Doncaster	19,869	14,736	74.2	73.6	74.8
E06000053	Isles of Scilly	482	353	73.2	69.1	77.0
E09000022	Lambeth	35,757	26,172	73.2	72.7	73.7
E09000010	Enfield	38,337	27,370	71.4	70.9	71.8
E09000005	Brent	68,977	48,573	70.4	70.1	70.8
E08000002	Bury	31,309	21,979	70.2	69.7	70.7
E09000002	Barking and Dagenham	36,578	25,402	69.4	69.0	69.9
E09000026	Redbridge	51,865	35,942	69.3	68.9	69.7
E06000021	Stoke-on-Trent	55,178	37,866	68.6	68.2	69.0
E06000008	Blackburn with Darwen	17,852	12,192	68.3	67.6	69.0
E08000030	Walsall	49,943	33,947	68.0	67.6	68.4
E09000023	Lewisham	26,396	17,838	67.6	67.0	68.1
E08000016	Barnsley	51,420	34,550	67.2	66.8	67.6
E09000009	Ealing	61,109	40,012	65.5	65.1	65.9
E06000039	Slough	16,191	10,600	65.5	64.7	66.2
E09000017	Hillingdon	45,539	29,447	64.7	64.2	65.1
E08000007	Stockport	44,540	28,763	64.6	64.1	65.0
E08000005	Rochdale	36,853	22,967	62.3	61.8	62.8
E09000015	Harrow	29,691	18,476	62.2	61.7	62.8
E06000047	County Durham	120,544	73,877	61.3	61.0	61.6
E09000019	Islington	38,209	23,415	61.3	60.8	61.8
E08000033	Calderdale	41,631	25,247	60.6	60.2	61.1
E09000031	Waltham Forest	50,680	30,720	60.6	60.2	61.0
E08000034	Kirklees	97,779	59,189	60.5	60.2	60.8
E10000029	Suffolk	147,142	89,051	60.5	60.3	60.8
E09000032	Wandsworth	57,469	34,442	59.9	59.5	60.3
E08000025	Birmingham	178,771	106,909	59.8	59.6	60.0
E06000036	Bracknell Forest	19,697	11,778	59.8	59.1	60.5
E10000019	Lincolnshire	200,192	119,037	59.5	59.2	59.7
E06000046	Isle of Wight	24,068	14,251	59.2	58.6	59.8
E08000004	Oldham	34,227	20,184	59.0	58.4	59.5
E06000031	Peterborough	44,281	26,027	58.8	58.3	59.2
E06000025	South Gloucestershire	59,350	34,683	58.4	58.0	58.8

For peer review only

For peer review only - http://bmjopen.bmj.com/site/about/guidelines.xhtml

E09000014	Haringey	29,867	17,448	58.4	57.9	59.0
E08000022	North Tyneside	40,154	23,434	58.4	57.9	58.8
E06000013	North Lincolnshire	24,121	13,870	57.5	56.9	58.1
E10000017	Lancashire	218,451	125,262	57.3	57.1	57.5
E06000005	Darlington	27,163	15,546	57.2	56.6	57.8
E06000011	East Riding of Yorkshire	12,161	6,894	56.7	55.8	57.6
E10000003	Cambridgeshire	116,035	65,679	56.6	56.3	56.9
E08000018	Rotherham	7,953	4,476	56.3	55.2	57.4
E06000016	Leicester	40,169	22,547	56.1	55.6	56.6
E06000034	Thurrock	32,083	17,982	56.0	55.5	56.6
E09000018	Hounslow	44,165	24,579	55.7	55.2	56.1
E10000006	Cumbria	120,237	65,183	54.2	53.9	54.5
E06000040	Windsor and Maidenhead	21,114	11,418	54.1	53.4	54.7
E06000057	Northumberland	75,940	40,859	53.8	53.4	54.2
E10000034	Worcestershire	141,667	76,000	53.6	53.4	53.9
E10000012	Essex	331,942	178,015	53.6	53.5	53.8
E10000024	Nottinghamshire	198,187	106,221	53.6	53.4	53.8
E09000024	Merton	43,144	23,114	53.6	53.1	54.0
E06000022	Bath and North East Somerset	44,466	23,810	53.5	53.1	54.0
E06000004	Stockton-on-Tees	35,341	18,857	53.4	52.8	53.9
E08000014	Sefton	48,044	25,630	53.3	52.9	53.8
E08000026	Coventry	64,356	34,306	53.3	52.9	53.7
E06000002	Middlesbrough	23,037	12,243	53.1	52.5	53.8
E08000019	Sheffield	80,302	42,628	53.1	52.7	53.4
E10000007	Derbyshire	197,165	104,520	53.0	52.8	53.2
E08000035	Leeds	174,645	92,288	52.8	52.6	53.1
E06000003	Redcar and Cleveland	25,185	13,304	52.8	52.2	53.4
E08000015	Wirral	80,558	42,456	52.7	52.4	53.0
E10000027	Somerset	75,851	39,814	52.5	52.1	52.8
E10000015	Hertfordshire	200,153	104,948	52.4	52.2	52.7
E09000016	Havering	42,627	22,305	52.3	51.9	52.8
E06000012	North East Lincolnshire	38,004	19,816	52.1	51.6	52.6
E08000029	Solihull	32,476	16,930	52.1	51.6	52.7
E10000013	Gloucestershire	137,245	71,077	51.8	51.5	52.1
E06000045	Southampton	33,058	17,102	51.7	51.2	52.3
E06000038	Reading	8,400	4,338	51.6	50.6	52.7
E06000027	Torbay	31,524	16,268	51.6	51.1	52.2
E06000024	North Somerset	40,162	20,498	51.0	50.5	51.5
E06000001	Hartlepool	12,989	6,616	50.9	50.1	51.8
E09000027	Richmond upon Thames	33,597	17,021	50.7	50.1	51.2
E06000033	Southend-on-Sea	48,006	24,182	50.4	49.9	50.8
E06000054	Wiltshire	114,656	57,526	50.2	49.9	50.5
E10000031	Warwickshire	102,623	51,428	50.1	49.8	50.4
E09000029	Sutton	24,049	11,959	49.7	49.1	50.4

For peer review only

E10000025	Oxfordshire	175,246	87,139	49.7	49.5	50.0
E06000056	Central Bedfordshire	73,732	36,607	49.6	49.3	50.0
E08000021	Newcastle upon Tyne	32,888	16,287	49.5	49.0	50.1
E10000021	Northamptonshire	155,686	76,979	49.4	49.2	49.7
E09000003	Barnet	52,312	25,849	49.4	49.0	49.8
E08000006	Salford	34,274	16,934	49.4	48.9	49.9
E06000019	Herefordshire, County of	37,499	18,421	49.1	48.6	49.6
E06000018	Nottingham	52,693	25,880	49.1	48.7	49.5
E06000043	Brighton and Hove	33,275	16,336	49.1	48.6	49.6
E06000030	Swindon	18,496	9,078	49.1	48.4	49.8
E06000023	Bristol, City of	58,017	28,467	49.1	48.7	49.5
E09000033	Westminster	48,724	23,723	48.7	48.2	49.1
E06000051	Shropshire	67,337	32,700	48.6	48.2	48.9
E08000028	Sandwell	39,552	19,164	48.5	48.0	48.9
E06000042	Milton Keynes	63,247	30,510	48.2	47.9	48.6
E08000036	Wakefield	61,543	29,680	48.2	47.8	48.6
E06000010	Kingston upon Hull, City of	17,074	8,219	48.1	47.4	48.9
E06000055	Bedford	31,728	15,205	47.9	47.4	48.5
E06000049	Cheshire East	52,794	25,264	47.9	47.4	48.3
E10000011	East Sussex	118,596	56,747	47.8	47.6	48.1
E08000009	Trafford	38,971	18,629	47.8	47.3	48.3
E06000044	Portsmouth	25,966	12,359	47.6	47.0	48.2
E06000059	Dorset	51,066	24,250	47.5	47.1	47.9
E08000023	South Tyneside	33,636	15,962	47.5	46.9	48.0
E10000030	Surrey	74,960	35,532	47.4	47.0	47.8
E06000015	Derby	62,407	29,315	47.0	46.6	47.4
E06000032	Luton	48,454	22,742	46.9	46.5	47.4
E08000008	Tameside	42,845	20,077	46.9	46.4	47.3
E10000008	Devon	105,836	49,495	46.8	46.5	47.1
E09000013	Hammersmith and Fulham	43,237	20,205	46.7	46.3	47.2
E09000007	Camden	44,662	20,798	46.6	46.1	47.0
E10000023	North Yorkshire	160,704	74,128	46.1	45.9	46.4
E09000004	Bexley	41,045	18,789	45.8	45.3	46.3
E08000003	Manchester	36,987	16,930	45.8	45.3	46.3
E10000028	Staffordshire	99,238	45,042	45.4	45.1	45.7
E08000013	St. Helens	35,045	15,868	45.3	44.8	45.8
E08000011	Knowsley	31,100	14,066	45.2	44.7	45.8
E06000058	Bournemouth, Christchurch and Poole	43,888	19,839	45.2	44.7	45.7
E06000020	Telford and Wrekin	34,384	15,444	44.9	44.4	45.4
E06000009	Blackpool	28,193	12,621	44.8	44.2	45.3
Unknown	Unknown	7,197	3,217	44.7	43.6	45.9
E10000002	Buckinghamshire	136,674	61,016	44.6	44.4	44.9

For peer review only - http://bmjopen.bmj.com/site/about/guidelines.xhtml

E10000032	West Sussex	90,033	40,022	44.5	44.1	44.8
E06000006	Halton	26,863	11,753	43.8	43.2	44.3
E06000052	Cornwall	48,099	20,877	43.4	43.0	43.8
E06000050	Cheshire West and Chester	40,408	17,537	43.4	42.9	43.9
E06000035	Medway	60,300	26,064	43.2	42.8	43.6
E10000020	Norfolk	161,582	69,173	42.8	42.6	43.1
E06000017	Rutland	6,741	2,862	42.5	41.3	43.6
E09000006	Bromley	75,672	31,841	42.1	41.7	42.4
E10000016	Kent	347,229	145,984	42.0	41.9	42.2
E09000008	Croydon	29,612	12,399	41.9	41.3	42.4
E09000011	Greenwich	32,488	13,547	41.7	41.2	42.2
E06000014	York	20,330	8,385	41.2	40.6	41.9
E08000027	Dudley	78,489	32,316	41.2	40.8	41.5
E06000026	Plymouth	28,855	11,707	40.6	40.0	41.1
E08000012	Liverpool	99,029	40,074	40.5	40.2	40.8
E10000018	Leicestershire	172,437	69,666	40.4	40.2	40.6
E08000024	Sunderland	47,131	18,370	39.0	38.5	39.4
E09000020	Kensington and Chelsea	35,607	13,811	38.8	38.3	39.3
E06000007	Warrington	48,004	18,287	38.1	37.7	38.5
E08000031	Wolverhampton	32,226	12,091	37.5	37.0	38.0
E08000010	Wigan	53,620	19,638	36.6	36.2	37.0
E09000021	Kingston upon Thames	32,087	11,529	35.9	35.4	36.5
E06000041	Wokingham	5,010	1,621	32.4	31.1	33.7
E08000037	Gateshead	49,663	14,497	29.2	28.8	29.6
E06000037	West Berkshire	16,235	4,376	27.0	26.3	27.6
E08000032	Bradford	82,669	20,791	25.1	24.9	25.4

Supplementary Table 10: Number of invitations recorded for attendees and non-attendees

**Number of invitations**	**Attendees n(%)**	**Non-attendees n(%)**
0	1,672,844 (32.8)	51,739 (1.1)
1	2,577,581 (50.5)	3,369,517 (73.4)
2	677,783 (13.3)	783,472 (17.1)
> 2	174,550 (3.4)	387,493 (8.4)
**TOTAL**	**5,102,758 (100.0)**	**4,592,221 (100.0)**

For peer review only - http://bmjopen.bmj.com/site/about/guidelines.xhtml

Supplementary Table 11: Invitations by financial year

Proportion of attendees and non-attendees with an invitation recorded

Year	Attendees with invitation	% attendees	Non-attendees with invitation	% non-attendees
2012/13	468,766	63.1	718,527	99.0
2013/14	619,559	64.3	824,429	98.9
2014/15	763,444	67.2	1,016,155	99.0
2015/16	790,731	69.2	999,178	98.7
2016/17	787,414	70.4	982,193	98.8
**TOTAL**	**3,429,914**	**67.2**	**4,540,482**	**98.9**

Supplementary Table 12: Completeness of risk factor measurement

Percentage of NHSHC attendees and non-attendees with recorded risk factor measurements (restricted to 15-month window around index date for attendees and unrestricted for non-attendees)

Group	CVD risk score	Body Mass Index	Physical Activity (GPPAQ)	Alcohol (Audit C)	Fasting glucose	HbA1C	Smoking Status	Cholesterol (HDL)	Cholesterol (total)	Diastolic BP	Systolic BP
Attendees	79.7%	96.3%	64.5%	38.3%	18.2%	36.6%	95.7%	87.2%	93.6%	95.7%	95.8%
Non-attendees	30.4%	55.0%	13.9%	16.7%	15.1%	37.5%	71.8%	47.3%	50.0%	76.3%	76.3%

For peer review only - http://bmjopen.bmj.com/site/about/guidelines.xhtml

Supplementary Table 13: Statin prescription rates

New statin (any dose) prescriptions among the subset (60.4%) of NHSHC attendees in whom medication data was available

Group	Attendees (n)	Prescribed a statin (n)	Proportion (%)
CVD score <10%	1,910,919	63,227	3.3
10-19.9%	532,046	83,279	15.7
≥20%	132,366	51,691	39.1
No CVD score	504,374	55,630	11.0
**Overall total**	**3,079,705**	**253,827**	**8.2**

For peer review only

The RECORD statement – checklist of items, extended from the STROBE statement, that should be reported in observational studies using routinely collected health data.

	Item No.	STROBE items	Location in manuscript where items are reported	RECORD items	Location in manuscript where items are reported
**Title and abstract**					
	1	(a) Indicate the study's design with a commonly used term in the title or the abstract (b) Provide in the abstract an informative and balanced summary of what was done and what was found	a) title<br>b) abstract	RECORD 1.1: The type of data used should be specified in the title or abstract. When possible, the name of the databases used should be included.<br><br>RECORD 1.2: If applicable, the geographic region and timeframe within which the study took place should be reported in the title or abstract.<br><br>RECORD 1.3: If linkage between databases was conducted for the study, this should be clearly stated in the title or abstract.	1.1 Title<br>1.2 Title<br>1.3 n/a
**Introduction**					
Background rationale	2	Explain the scientific background and rationale for the investigation being reported	Introduction		
Objectives	3	State specific objectives, including any prespecified hypotheses	Introduction		
**Methods**					
Study Design	4	Present key elements of study design early in the paper	Study design		
Setting	5	Describe the setting, locations, and relevant dates, including periods of recruitment, exposure, follow-up, and data collection	Study setting		

For peer review only

Participants	6	*(a) Cohort study* - Give the eligibility criteria, and the sources and methods of selection of participants. Describe methods of follow-up<br>*Case-control study* - Give the eligibility criteria, and the sources and methods of case ascertainment and control selection. Give the rationale for the choice of cases and controls<br>*Cross-sectional study* - Give the eligibility criteria, and the sources and methods of selection of participants<br><br>*(b) Cohort study* - For matched studies, give matching criteria and number of exposed and unexposed<br>*Case-control study* - For matched studies, give matching criteria and the number of controls per case	Cross-sectional Study population	RECORD 6.1: The methods of study population selection (such as codes or algorithms used to identify subjects) should be listed in detail. If this is not possible, an explanation should be provided.<br><br>RECORD 6.2: Any validation studies of the codes or algorithms used to select the population should be referenced. If validation was conducted for this study and not published elsewhere, detailed methods and results should be provided.<br><br>RECORD 6.3: If the study involved linkage of databases, consider use of a flow diagram or other graphical display to demonstrate the data linkage process, including the number of individuals with linked data at each stage.	6.1 Figure 1 & Supplement<br>6.2 Because the extract consists only of those with NHSHC codes, we are unable to carry out validation studies. Instead we present completeness of data.<br>6.3 N/A
Variables	7	Clearly define all outcomes, exposures, predictors, potential confounders, and effect modifiers. Give diagnostic criteria, if applicable.	Methods. Variables	RECORD 7.1: A complete list of codes and algorithms used to classify exposures, outcomes, confounders, and effect modifiers should be provided. If these cannot be reported, an explanation should be provided.	7.1 Supplement
Data sources/ measurement	8	For each variable of interest, give sources of data and details of methods of assessment (measurement).<br>Describe comparability of assessment methods if there is more than one group	Methods- variables and Supplement		

For peer review only

Bias	9	Describe any efforts to address potential sources of bias	Methods- data presentation		
Study size	10	Explain how the study size was arrived at	Methods Figure 1		
Quantitative variables	11	Explain how quantitative variables were handled in the analyses. If applicable, describe which groupings were chosen, and why	Methods- Variables		
Statistical methods	12	(a) Describe all statistical methods, including those used to control for confounding<br>(b) Describe any methods used to examine subgroups and interactions<br>(c) Explain how missing data were addressed<br>(d) *Cohort study* - If applicable, explain how loss to follow-up was addressed<br>*Case-control study* - If applicable, explain how matching of cases and controls was addressed<br>*Cross-sectional study* - If applicable, describe analytical methods taking account of sampling strategy<br>(e) Describe any sensitivity analyses	Methods- data presentation		
Data access and cleaning methods		..		RECORD 12.1: Authors should describe the extent to which the investigators had access to the database population used to create the study population.	12.1 methods- study setting<br>12.2 methods – data management and cleaning & Figure 1, Supplement

For peer review only

				RECORD 12.2: Authors should provide information on the data cleaning methods used in the study.	
Linkage		..		RECORD 12.3: State whether the study included person-level, institutional-level, or other data linkage across two or more databases. The methods of linkage and methods of linkage quality evaluation should be provided.	12.3 – Methods-Study design individual level data<br>n/a on linkage
**Results**					
Participants	13	(a) Report the numbers of individuals at each stage of the study (*e.g.*, numbers potentially eligible, examined for eligibility, confirmed eligible, included in the study, completing follow-up, and analysed)<br>(b) Give reasons for non-participation at each stage.<br>(c) Consider use of a flow diagram	a) Figure 1<br>& Overall uptake by year<br>b) figure 1<br>c) Figure 1	RECORD 13.1: Describe in detail the selection of the persons included in the study (*i.e.,* study population selection) including filtering based on data quality, data availability and linkage. The selection of included persons can be described in the text and/or by means of the study flow diagram.	Figure 1
Descriptive data	14	(a) Give characteristics of study participants (*e.g.*, demographic, clinical, social) and information on exposures and potential confounders<br>(b) Indicate the number of participants with missing data for each variable of interest<br>(c) *Cohort study* - summarise follow-up time (*e.g.*, average and total amount)	a) Table 1<br>b) Table 1		
Outcome data	15	*Cohort study* - Report numbers of outcome events or summary measures over time<br>*Case-control study* - Report numbers in each exposure	No outcome reported – described data for attendees and non-attendees		

		category, or summary measures of exposure *Cross-sectional study* - Report numbers of outcome events or summary measures			
Main results	16	(a) Give unadjusted estimates and, if applicable, confounder-adjusted estimates and their precision (e.g., 95% confidence interval). Make clear which confounders were adjusted for and why they were included (b) Report category boundaries when continuous variables were categorized (c) If relevant, consider translating estimates of relative risk into absolute risk for a meaningful time period	a) n/a b) Supplement c) n/a		
Other analyses	17	Report other analyses done—e.g., analyses of subgroups and interactions, and sensitivity analyses	n/a		
**Discussion**					
Key results	18	Summarise key results with reference to study objectives	Discussion		
Limitations	19	Discuss limitations of the study, taking into account sources of potential bias or imprecision. Discuss both direction and magnitude of any potential bias	Limitations	RECORD 19.1: Discuss the implications of using data that were not created or collected to answer the specific research question(s). Include discussion of misclassification bias, unmeasured confounding, missing data, and changing eligibility over time, as they pertain to the study being reported.	
Interpretation	20	Give a cautious overall interpretation of results considering objectives,	Discussion, Conclusion		

For peer review only

		limitations, multiplicity of analyses, results from similar studies, and other relevant evidence			
Generalisability	21	Discuss the generalisability (external validity) of the study results	n/a		
**Other Information**					
Funding	22	Give the source of funding and the role of the funders for the present study and, if applicable, for the original study on which the present article is based	Funding		
Accessibility of protocol, raw data, and programming code		..		RECORD 22.1: Authors should provide information on how to access any supplemental information such as the study protocol, raw data, or programming code.	Code on GITHUB

*Reference: Benchimol EI, Smeeth L, Guttmann A, Harron K, Moher D, Petersen I, Sørensen HT, von Elm E, Langan SM, the RECORD Working Committee. The REporting of studies Conducted using Observational Routinely-collected health Data (RECORD) Statement. *PLoS Medicine* 2015; in press.

*Checklist is protected under Creative Commons Attribution (CC BY) license.

For peer review only
